# In vitro and in silico parameters for precise cgMLST typing of *Listeria monocytogenes*

**DOI:** 10.1186/s12864-022-08437-4

**Published:** 2022-03-26

**Authors:** Federica Palma, Iolanda Mangone, Anna Janowicz, Alexandra Moura, Alexandra Chiaverini, Marina Torresi, Giuliano Garofolo, Alexis Criscuolo, Sylvain Brisse, Adriano Di Pasquale, Cesare Cammà, Nicolas Radomski

**Affiliations:** 1 Institut Pasteur, Université de Paris, Biological Resources Center of Institut Pasteur, 75015 Paris, France; 2 Istituto Zooprofilattico Sperimentale dell’Abruzzo e del Molise “Giuseppe Caporale” (IZSAM), National Reference Centre (NRC) for Whole Genome Sequencing of microbial pathogens: data-base and bioinformatics analysis (GENPAT), via Campo Boario, 64100 Teramo, TE Italy; 3 Istituto Zooprofilattico Sperimentale dell’Abruzzo e del Molise “Giuseppe Caporale” (IZSAM), Bacteriology Unit, via Campo Boario, 64100 Teramo, TE Italy; 4 Institut Pasteur, National Reference Center and WHO Collaborating Center Listeria, 75015 Paris, France; 5 Institut Pasteur, Université de Paris, Inserm U1117, Biology of Infection Unit, 75015 Paris, France; 6 Istituto Zooprofilattico Sperimentale dell’Abruzzo e del Molise “Giuseppe Caporale” (IZSAM), National Reference Labororatory (LNR) for Listeria monocytogenes, via Campo Boario, 64100 Teramo, TE Italy; 7 Institut Pasteur, Université de Paris, Bioinformatics and Biostatistics Hub, 75015 Paris, France; 8 Institut Pasteur, Université de Paris, Biodiversity and Epidemiology of Bacterial Pathogens, 75015 Paris, France

**Keywords:** cgMLST, Comparability of workflows, *Listeria monocytogenes*, Principal component analysis, Generalized linear model

## Abstract

**Background:**

Whole genome sequencing analyzed by core genome multi-locus sequence typing (cgMLST) is widely used in surveillance of the pathogenic bacteria *Listeria monocytogenes*. Given the heterogeneity of available bioinformatics tools to define cgMLST alleles, our aim was to identify parameters influencing the precision of cgMLST profiles.

**Methods:**

We used three *L. monocytogenes* reference genomes from different phylogenetic lineages and assessed the impact of in vitro (i.e. tested genomes, successive platings, replicates of DNA extraction and sequencing) and in silico parameters (i.e. targeted depth of coverage, depth of coverage, breadth of coverage, assembly metrics, cgMLST workflows, cgMLST completeness) on cgMLST precision made of 1748 core loci. Six cgMLST workflows were tested, comprising assembly-based (BIGSdb, INNUENDO, GENPAT, SeqSphere and BioNumerics) and assembly-free (i.e. kmer-based MentaLiST) allele callers. Principal component analyses and generalized linear models were used to identify the most impactful parameters on cgMLST precision.

**Results:**

The isolate’s genetic background, cgMLST workflows, cgMLST completeness, as well as depth and breadth of coverage were the parameters that impacted most on cgMLST precision (i.e. identical alleles against reference circular genomes). All workflows performed well at ≥40X of depth of coverage, with high loci detection (> 99.54% for all, except for BioNumerics with 97.78%) and showed consistent cluster definitions using the reference cut-off of ≤7 allele differences.

**Conclusions:**

This highlights that bioinformatics workflows dedicated to cgMLST allele calling are largely robust when paired-end reads are of high quality and when the sequencing depth is ≥40X.

**Supplementary Information:**

The online version contains supplementary material available at 10.1186/s12864-022-08437-4.

## Introduction

A key component of the surveillance of microbial pathogens is the recognition of closely related strains, so that clusters of infection cases can be identified, and further investigations (e.g., identification of the source of contamination) and control measures taken [1]. Multi-locus sequence typing (MLST) was developed in 1998 and provided high reproducibility in the characterization of isolates, enabling to identify the same clones within bacterial populations [2]. However, it lacks discrimination at the strain level [3–5]. With the advances in whole genome sequencing (WGS) [6–9], core genome MLST (cgMLST) tools and schemes have been proposed for several bacterial pathogens, expanding the advantages of MLST at the genomic scale and providing a high level of bacterial strain discrimination. cgMLST relies on defining alleles for thousands of gene loci, translating sequence variation into numerical profiles, which are computationally easier and faster to handle and analyze, as compared with genome-based sequence alignments [10, 11].

Different commercial and open-source solutions have been proposed for cgMLST, differing in the type of input data (i.e. reads and/or assemblies), in the allele definition strategies (i.e. algorithms based on nucleotide alignments, protein-coding genes predictions, or kmer counting) and in settings used to generate cgMLST profiles [12–20]. Multi-center ring trials focusing on reproducibility and comparability of cgMLST-based bacterial typing and clustering showed discrepancies due to non-harmonized bioinformatic workflows that may affect the precision of WGS-based surveillance and outbreaks investigation [21, 83].

Distinct core genome-based MLST schemes have been proposed for high resolution typing of the foodborne pathogen *Listeria monocytogenes*, ranging from 1013 to 1827 loci [12, 23–25], including an open-source reference cgMLST scheme of 1748 gene loci that is used worldwide [26, 28, 28, 30, 31, 31] and curated in the open-source Bacterial Isolate Genome Sequence database (BIGSdb) [26].

Several parameters such as genetic background of tested strains [32], successive platings [33, 38], replicates of DNA extraction and sequencing [35], targeted depth of coverage [24, 28, 36], estimated depth and breadth of coverage [13, 38, 42] and assembly quality [39], may impact alleles called, compromising cgMLST profiles reproducibility and the definition of outbreak clusters.

We therefore aimed to identify in vitro and in silico parameters impacting the precision of cgMLST profiles from six cgMLST complete workflows while assessing clustering concordance using the cut-off of 7 alleles mismatches [24]. Our study represents a substantial extension in terms of number of assessed allele callers and parameters of the study recently published by Lüth et al. (2021) [46].

## Results

**Fig. 1:**
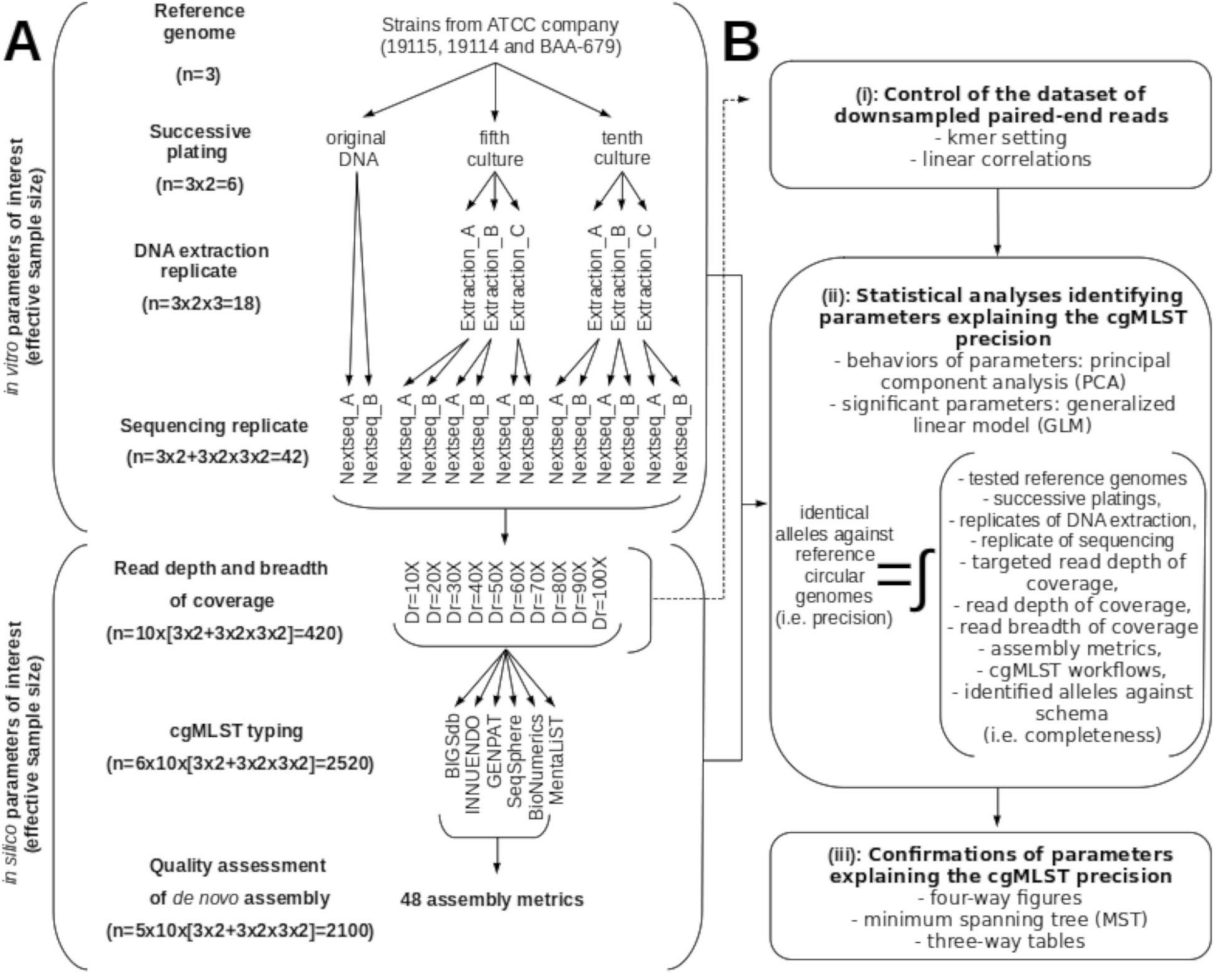
Experimental plan (A) and data analyses (B) aiming at controlling a dataset of downsampled paired-end reads (i: *n* = 420 paired-end reads), as well as identifying statistically (ii) and confirming significant parameters (iii) explaining the *Listeria monocytogenes* cgMLST precision (identical alleles against circular reference genomes: *n* = 2520 cgMLST typing) among in vitro (i.e. tested reference genomes, successive platings, as well as replicates of DNA extraction and sequencing) and in silico (i.e. targeted depth of coverage, read depth and breadth of coverage, assembly metrics, cgMLST workflows, identified alleles against schema) parameters (*n* = 57 parameters of interest)

**Fig. 2:**
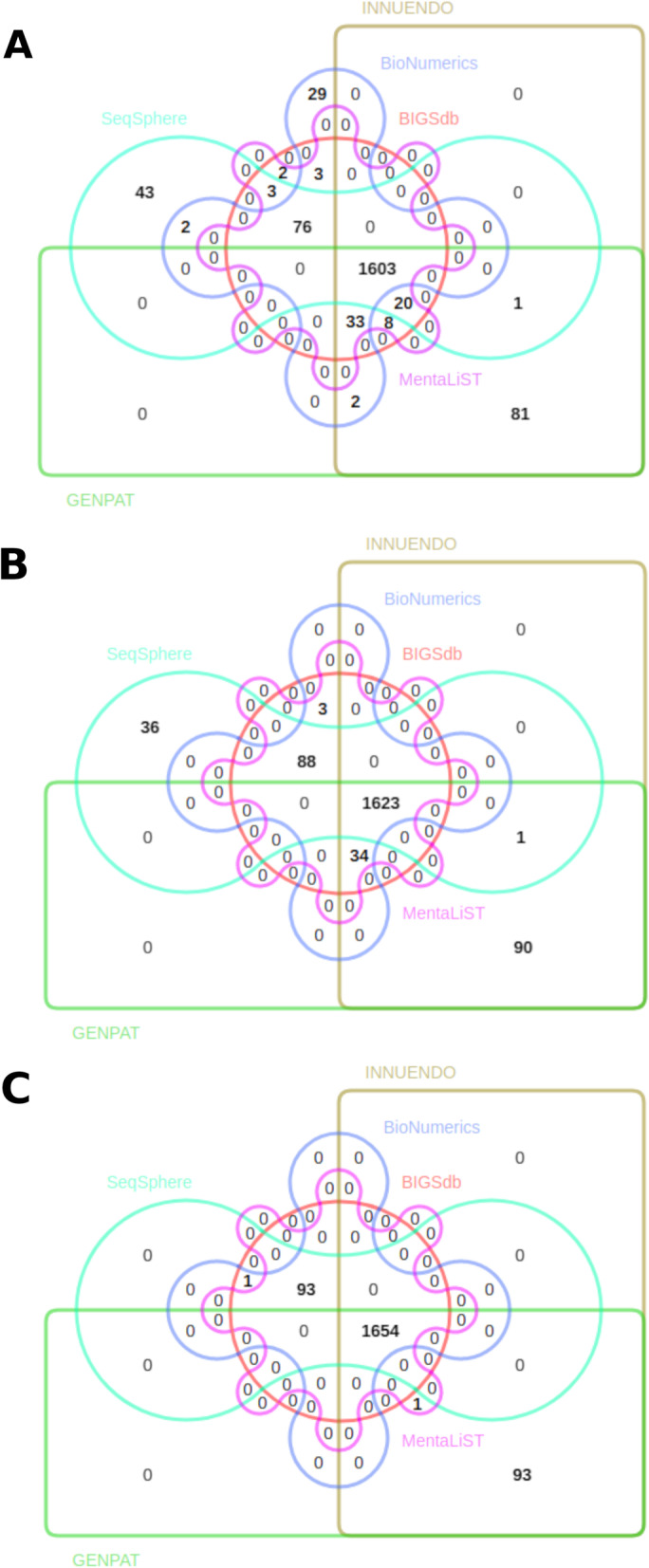
Edward’s Venn diagrams representing the identical alleles between the cgMLST workflows BIGSdb, INNUENDO, GENPAT, SeqSphere, BioNumerics and MentaLiST for the *Listeria monocytogenes* reference circular genomes ATCC19114 (A), ATCC19115 (B) and ATCCBAA679 (C)

The experimental plan set-up for this study (Fig. 1A) allowed us to build an accurate dataset of paired-end reads controlling the depth of coverage (Fig. 1B-i), statistically identify parameters explaining the cgMLST precision among a large set of in vitro and in silico parameters (Fig. 1B-ii), and illustrate graphically those parameters explaining the cgMLST precision (Fig. 1B-iii). Here, we focused on the precision (i.e. identical alleles against reference circular genomes (IAAR)) and completeness (i.e. identified alleles against schema (IAAS)) of cgMLST profiles, rather than accuracy, because allele differences were observed when comparing cgMLST profiles of reference circular genomes from compared cgMLST workflows (i.e. BIGSdb, INNUENDO, GENPAT, SeqSphere, BioNumerics and MentaLiST) (Fig. 2).

### Benchmarking dataset of downsampled reads

**Fig. 3:**
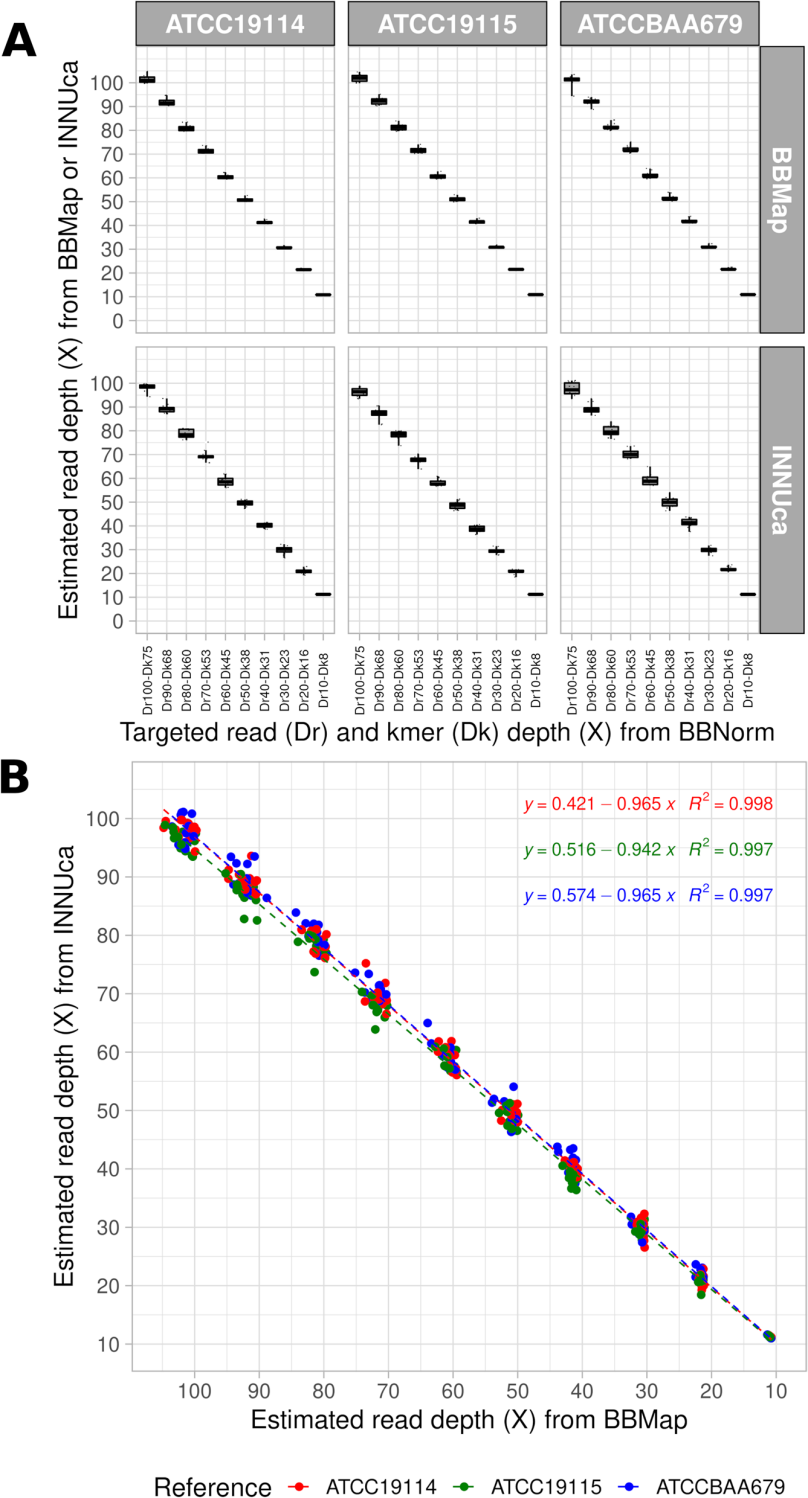
Boxplot-based distributions of targeted read (Dr) and kmer (Dk) depth (X) from BBNorm downsampling (read length *R =* 150 and kmer size K = 30) of *Listeria monocytogenes* paired-end reads from reference genomes ATCC19114, ATCC19115 and ATCCBAA679 (*n* = 420) according to estimated read depth (X) from BBMap (version February 13, 2020) or INNUca (version 4.2.2) with constant high read breadth of coverage (99.34 ± 0.07%) (A) and linear correlations between read depth of downsampled paired-end reads (*n =* 420) estimated with BBMap or INNUca for each reference genome (B)

Paired-end reads used for downsampling (*n* = 42) contained enough reads (3.77 ± 0.71 × 10^6^) to prepare a dataset of downsampled paired-end reads to process with the selected cgMLST workflows. This dataset presented the highest expected read depth of coverage (i.e. 100X), as well as high and stable average Phred quality scores (34.64 ± 0.07) and percentages of Phred quality scores higher than 30 (93.00 ± 1.29%). No single nucleotide variant (SNV) was detected during Confindr-based exogenous DNA contamination screening [41] in the dataset used for downsampling. Regardless of the targeted read depth of coverage (Dr) defined according to kmer depth (Dk) with BBNorm (ranging from 10X to 100X) [42], the breadth of coverage of the downsampled paired-end reads estimated with BBMap (*n* = 420) [42] was very high (> 99.3%) for each of the tested reference genomes (Table 1, Fig. 3A and Additional file 1). The accuracy of this downsampled reads was corroborated by the concordance (i.e. linear dependencies with slopes close to one) observed between the read depth of coverage estimated with BBMap [42] and INNUca [49] (R^2^ > 99.7%; Pearson test: *p* < 2 × 10^− 16^) for the three reference genomes of interest (Fig. 3B).

**Table 1:** Mean and standard deviation of read depth of coverage estimated from BBMap (version February 13, 2020) or INNUca (version 4.2.2) with constant high read breadth of coverage (99.34 ± 0.07%) according to targeted read (Dr) and kmer (Dk) depth (X) from BBNorm downsampling (read length *R =* 150 and kmer size K = 30) of *Listeria monocytogenes* paired-end reads from tested reference genomes ATCC19114, ATCC19115 and ATCCBAA679 (*n =* 420)

Targeted depth of coverage	ATCC19114		ATCC19115		ATCCBAA679	
	BBMap	INNUca	BBMap	INNUca	BBMap	INNUca
**Dr100-Dk75**	101.6 ± 1.6	98.2 ± 1.5	101.9 ± 1.4	96.2 ± 1.8	101.0 ± 2.1	97.6 ± 2.6
**Dr90-Dk68**	91.9 ± 1.5	89.3 ± 1.7	92.3 ± 1.4	87.2 ± 2.3	92.0 ± 1.3	89.2 ± 2.1
**Dr80-Dk60**	80.9 ± 1.3	78.7 ± 1.8	81.3 ± 1.2	78.3 ± 1.7	81.4 ± 1.1	79.9 ± 2.2
**Dr70-Dk53**	71.4 ± 1.1	69.5 ± 2.0	71.7 ± 1.1	67.8 ± 1.6	72.0 ± 1.3	70.4 ± 1.7
**Dr60-Dk45**	60.5 ± 0.9	58.7 ± 2.0	60.7 ± 0.9	58.1 ± 1.5	61.1 ± 1.2	59.3 ± 2.2
**Dr50-Dk38**	50.9 ± 0.8	49.5 ± 1.2	51.1 ± 0.8	48.6 ± 1.6	51.5 ± 1.1	49.9 ± 2.0
**Dr40-Dk31**	41.3 ± 0.6	40.1 ± 1.0	41.5 ± 0.6	38.7 ± 1.3	41.9 ± 0.9	41.3 ± 1.7
**Dr30-Dk23**	30.7 ± 0.4	29.9 ± 1.6	30.8 ± 0.4	29.5 ± 1.1	31.1 ± 0.6	29.8 ± 1.1
**Dr20-Dk16**	21.5 ± 0.2	20.9 ± 0.9	21.5 ± 0.2	20.7 ± 0.9	21.7 ± 0.4	21.8 ± 0.8
**Dr10-Dk8**	10.9 ± 0.1	11.3 ± 0.1	10.9 ± 0.1	11.2 ± 0.1	11.0 ± 0.2	11.2 ± 0.2

### Principal component analysis

**Fig. 4:**
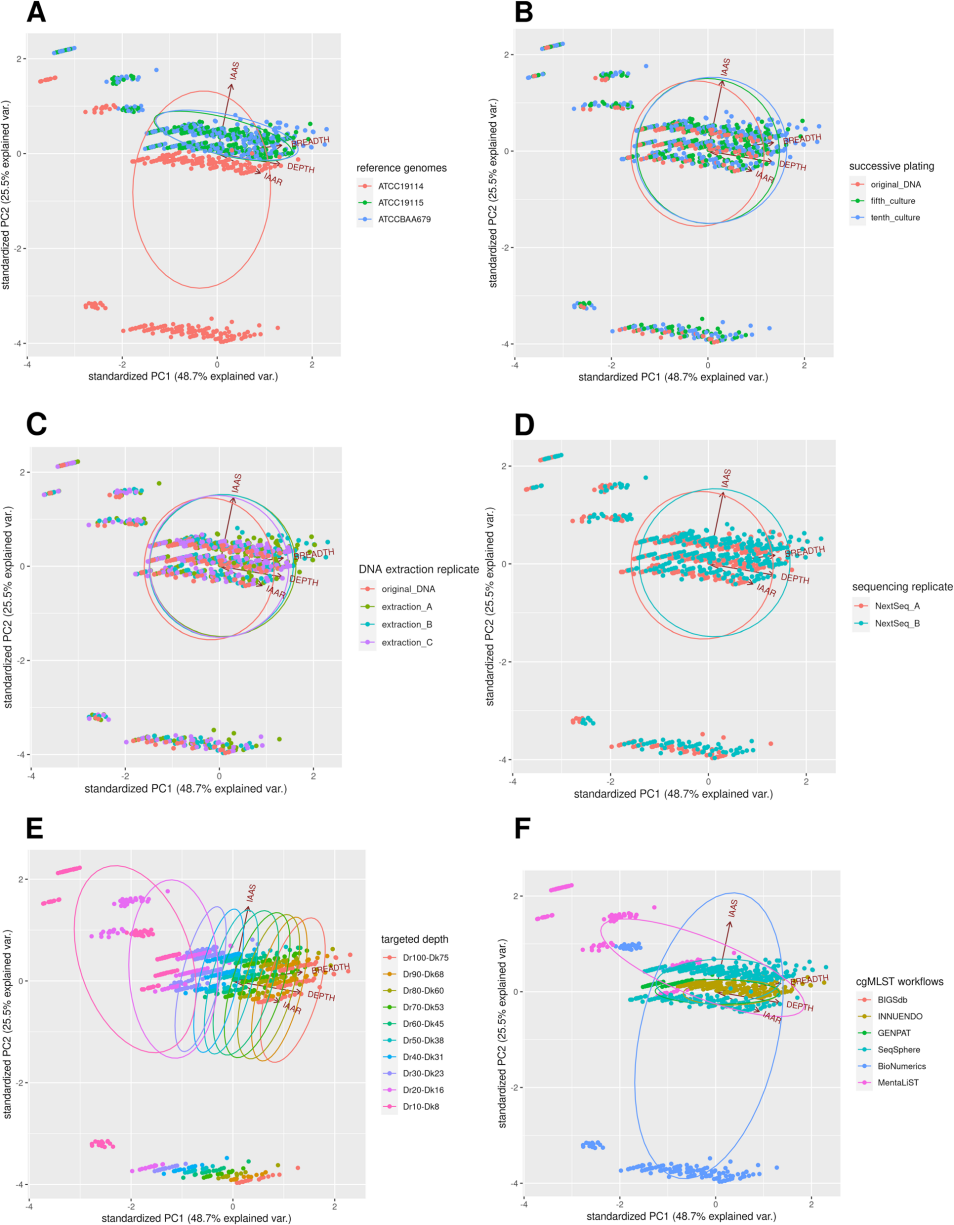
Principals component analyses (PCAs) of the numerical parameters IAAR, IAAS, DEPTH and BREADTH (defined in the section abbreviations) according to the categorical parameters “reference genome” (A), “successive platings” (B), “DNA extraction replicate” (C), “sequencing replicate” (D), “targeted depth” (E), “ cgMLST workflows” (F) including BIGSdb (*n =* 420), INNUENDO (n = 336), GENPAT (*n =* 420), SeqSphere (*n =* 420), BioNumerics (*n =* 420) and MentaLiST (*n =* 420) applied to downsampled paired-end reads from 3 reference genomes of *Listeria monocytogenes* (i.e. ATCC19114, ATCC19115 and ATCCBAA679). The PCA outcomes from the workflows BIGSdb and SeqSphere are overlapped

Principal component analyses (PCAs) were built according to investigated categorical parameters, namely tested genomes (A) successive platings (B), replicates of DNA extraction (C) and sequencing (D), targeted depth (E) and cgMLST workflows (F) (Fig. 4 and Additional file 4). PCAs showed that the investigated in vitro parameters (i.e. successive platings, DNA and sequencing replicates), did not impact the precision (i.e. IAAR) and completeness (i.e. IAAS) of cgMLST profiles (Fig. 4B-Fig. 4D; Additional file 4B-Additional file 4D). In contrast, the tested reference genomes (Fig. 4A), targeted depth (Fig. 4E) and cgMLST workflows (Fig. 4F) may influence the precision and completeness of cgMLST profiles. More precisely, high targeted read (Dr) and kmer (Dk) depth (DrDk) were associated with high IAAR values (Fig. 4E), depth and breadth of coverage, as well as LA, N50 and NA50 for the assembly-based workflows (Additional file 4E-Additional file 4F). Otherwise, low values of DrDk (Fig. 4E) were mainly associated with the workflows BioNumerics and MentaLiST (Fig. 4F; Additional file 4F). Low values of IAAS were associated with the reference genome ATCC19114 (Fig. 4A; Additional file 4A). Overall for assembly-based workflows, the decrease of cgMLST precision (i.e. IAAR) was associated with high values of MA, GC, TL1000 and TL10000 or high values of L50, LA50, C1000 and C10000 (Additional file 4).

### Generalized linear model

Generalized linear models (GLMs) were performed including all cgMLST workflows (A) or focusing on BIGSdb (B), INNUENDO (C), GENPAT (D), SeqSphere (E) and BioNumerics (F) (Additional file 5). The assembly metrics were not linearly correlated to cgMLST precision through GLMs (*p* > 1.0 × 10^− 3^) (Additional file 5). The GLM (Table 2) globally showed that IAAR (i.e. precision) was significantly explained by the workflow MentaLiST (*p* = 2.0 × 10^− 16^), breadth (*p* = 5.3 × 10^− 12^) and depth (*p* = 1.5 × 10^− 12^) of coverage, tested reference genome ATCCBAA679 (p = 2.0 × 10^− 16^), as well as amount of any base (N) per 100 kb (N100) (*p =* 2.0 × 10^− 16^) and IAAS (i.e. completeness) (*p* = 3.7 × 10^− 6^) for assembly-based cgMLST workflows (Additional file 5A). Looking at these workflows individually, the GLMs showed that IAAR was significantly explained by N100 (*p =* 2.0 × 10^− 16^) for BioNumerics (Additional file 5F), while poorly correlated linearly with the other parameters for BIGSdb (Additional file 5B: *p* > 9.1 × 10^− 1^), INNUENDO (Additional file 5C: *p* > 9.8 × 10^− 1^), GENPAT (Additional file 5D: *p* > 9.4 × 10^− 1^) and SeqSphere (Additional file 5E: *p* > 9.3 × 10^− 1^).

**Table 2:** Coefficients (Coef.) of the generalized linear model (GLMs with quasi Poisson distribution and with overdispersion) comparing the parameters “identical alleles against circular reference genomes” (IAAR) with the parameters of interest “tested reference genomes” (REFERENCE), “successive platings” (PLATING) (B), “DNA extraction replicate” (DNA), “sequencing replicate” (SEQUENCING), read depth (DEPTH), read breadth (BREADTH), identified alleles against schema (IAAS) and cgMLST workflows (WORKFLOW) including BIGSdb (*n =* 420), INNUENDO (*n =* 336), GENPAT (*n =* 420), SeqSphere (*n =* 420), BioNumerics (*n =* 420) and MentaLiST (*n =* 420) applied to downsampled paired-end reads from 3 tested reference genomes of *Listeria monocytogenes* (i.e. ATCC19114, ATCC19115 and ATCCBAA679). Few parameters are not defined because of singularities

Parameters	Coef. estimate	Coef. standard error	Coef. t value	Coef. ***P***-value (>|t|)
WORKFLOW: MentaLiST	-1.9E-01	1.1E-02	-1.7E+ 01	2.0E-16
BREADTH	5.6E-01	8.1E-02	6.9E+ 00	5.3E-12
DEPTH	8.1E-04	1.9E-04	4.3E+ 00	1.5E-05
REFERENCE: ATCCBAA679	−3.8E-02	1.1E-02	−3.6E+ 00	3.5E-04
SEQUENCING: NextSeq_B	−2.3E-02	7.0E-03	−3.2E+ 00	1.3E-03
REFERENCE: ATCC19115	−3.0E-02	1.0E-02	−3.0E+ 00	2.9E-03
WORKFLOW: BioNumerics	−2.9E-02	1.2E-02	−2.4E+ 00	1.7E-02
PLATING: tenth_culture	−2.4E-02	1.1E-02	−2.1E+ 00	3.3E-02
WORKFLOW: INNUENDO	−2.0E-02	1.1E-02	−1.8E+ 00	7.5E-02
PLATING: fifth_culture	−1.8E-02	1.1E-02	−1.6E+ 00	1.1E-01
IAAS	6.3E-04	5.1E-04	1.2E+ 00	2.2E-01
DNA: extraction_A	−7.4E-03	8.4E-03	−8.9E-01	3.7E-01
DNA: extraction_B	−4.5E-03	8.3E-03	−5.4E-01	5.9E-01
WORKFLOW: GENPAT	1.7E-04	1.1E-02	1.6E-02	9.9E-01
WORKFLOW: SeqSphere	4.5E-05	1.1E-02	4.3E-03	1.0E+ 00
**Model Intercept**	**−4.9E+ 01**	**8.1E+ 00**	**− 6.1E+ 00**	**1.1E-09**

### Graphically confirmations

**Fig. 5:**
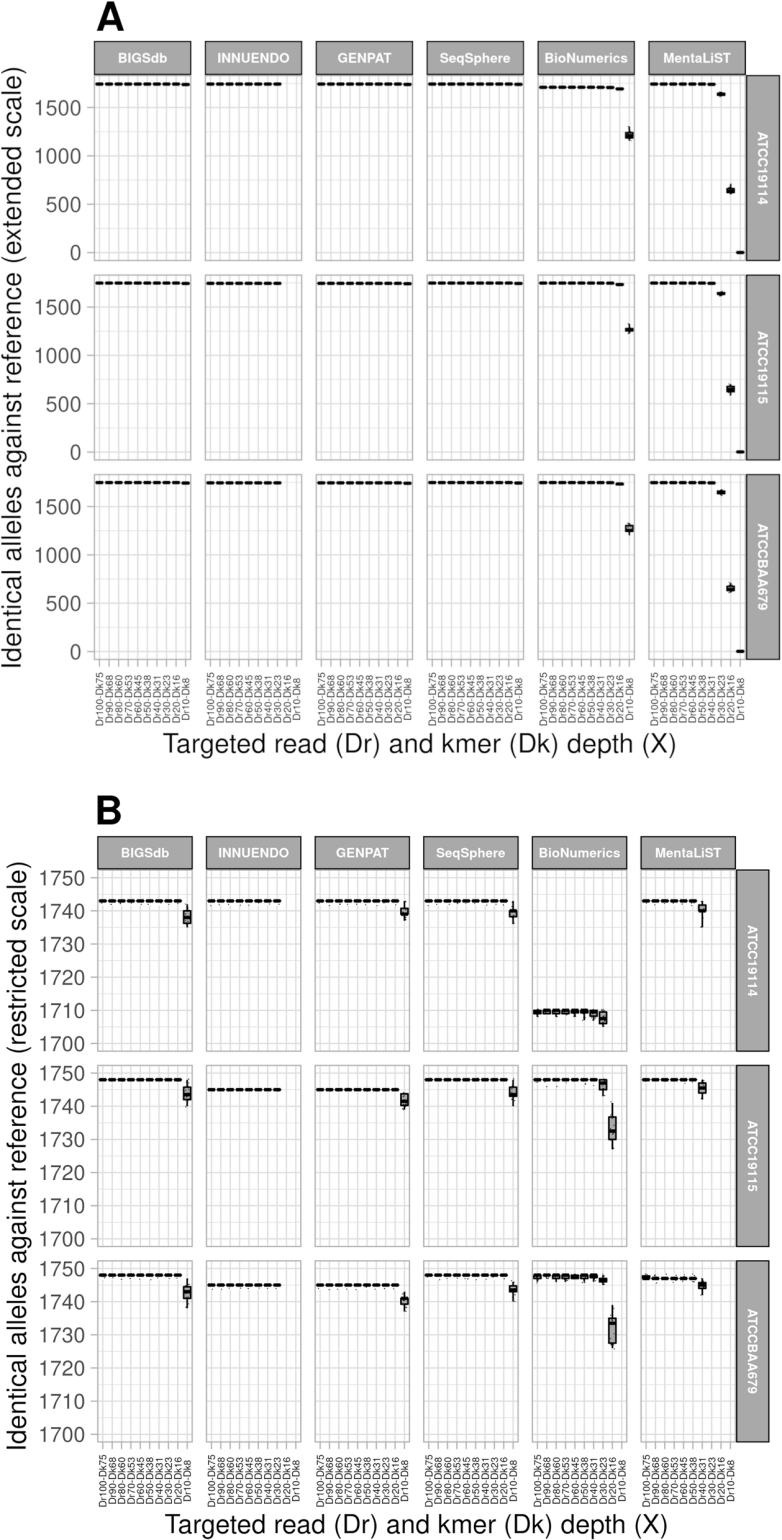
Box-plots representing the impact of downsampled paired-end reads (i.e. 2x150bp) of *Listeria monocytogenes* on identical alleles against reference at extended (A) or restricted (B) scales, according to reference genomes (i.e. ATCC19114, ATCC19115 and ATCCBAA679) and cgMLST workflows including BIGSdb (*n =* 420), INNUENDO (*n =* 336), GENPAT (*n =* 420), SeqSphere (*n =* 420), BioNumerics (*n =* 420) and MentaLiST (*n =* 420). The targeted read depth (Dr: 10X, 20X, 30X, 40X, 50X, 60X, 70X, 80X, 90X and 100X) were prepared according to kmer depth (Dk): 8X, 15X, 23X, 30X, 38X, 45X, 52X, 60X, 67X, 75X) setting of BBNorm (read length *R =* 150 and kmer size K = 30). Because of internal firewall, the INNUca assembler integrated into the cgMLST workflow INNUENDO cannot not perform assemblies of paired-end reads with read depth of coverage of 20X (*n =* 42) and 10X (*n =* 42)

**Fig. 6:**
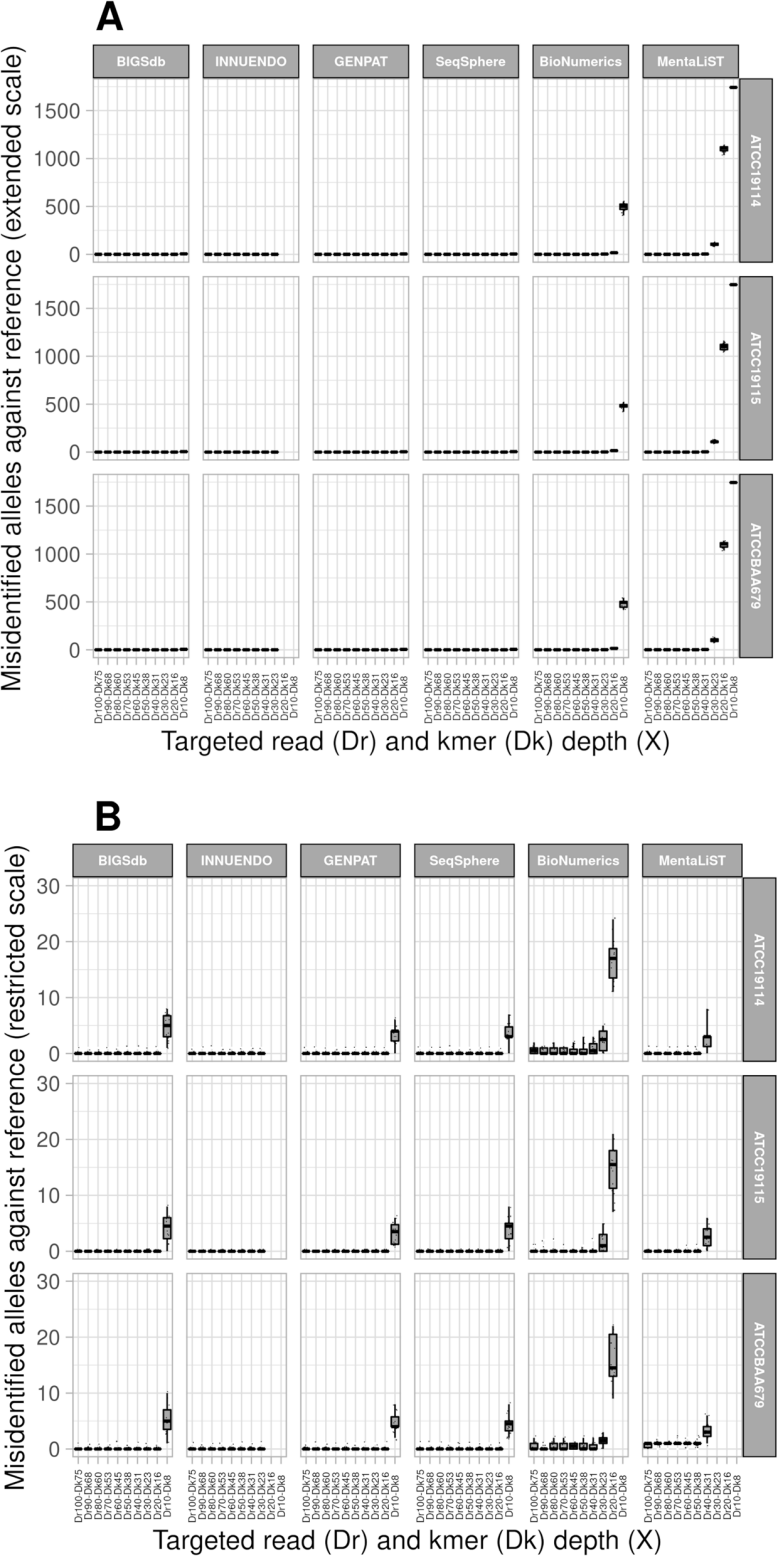
Box-plots representing the impact of downsampled paired-end reads (i.e. 2x150bp) of *Listeria monocytogenes* on misidentified alleles against reference at extended (A) or restricted (B) scales, according to reference genomes (i.e. ATCC19114, ATCC19115 and ATCCBAA679) and cgMLST workflows including BIGSdb (*n =* 420), INNUENDO (*n =* 336), GENPAT (*n =* 420), SeqSphere (*n =* 420), BioNumerics (*n =* 420) and MentaLiST (*n =* 420). The targeted read depth (Dr: 10X, 20X, 30X, 40X, 50X, 60X, 70X, 80X, 90X and 100X) were prepared according to kmer depth (Dk): 8X, 15X, 23X, 30X, 38X, 45X, 52X, 60X, 67X, 75X) setting of BBNorm (read length *R =* 150 and kmer size K = 30). Because of internal firewall, the INNUca assembler integrated into the cgMLST workflow INNUENDO cannot not perform assemblies of paired-end reads with read depth of coverage of 20X (*n =* 42) and 10X (*n =* 42)

The graphical representation in four-way figures were built including IAAS (A, B, C, D) or IAAR at extended (E, F, G, H) or restricted (I, J, K, L) scales, according to reference genomes (A, E, I), successive platings (B, F, J), DNA extraction replicate (C, G, K) and sequencing replicate (C, H, L) (Additional file 6). These four-way figures clearly showed that IAAS (i.e. completeness) were impacted by tested reference genomes and cgMLST workflows (Additional file 6A) but not by in vitro parameters (Additional file 6B-Additional file 6D). In fact, BioNumerics profiles showed the higher number of unidentified alleles (38 over 1748 loci) for ATCC19114 compared to the other workflows (5 over 1748) (Additional file 7). For ATCC19115 and ATCCBAA679, INNUENDO and GENPAT showed 3 unidentified alleles over 1748 loci (Additional file 7), while the other workflows identified all the loci of the schema. IAAR (i.e. precision) is impacted by DrDk, cgMLST workflows and tested reference genomes (Fig. 5). More precisely, IAAR of BioNumerics and MentaLiST sharply dropped down at depth of coverage of ~30X (up to 1686) and ~ 40X (up to 1614), respectively. While INNUENDO showed almost 100% of identical allele calls at ≥30X (as it filters out reads at ≤25X), BIGSdb, GENPAT and SeqSphere called almost 100% of IAAR at lower depth of coverage (≥20X) (Fig. 5). At this depth of coverage (~ 20X) the number of misidentified alleles against reference (MIAAR) of BioNumerics and MentaLiST was remarkably higher (i.e. > 7) compared to BIGSdb, GENPAT and SeqSphere that showed similar misidentified alleles against reference (MIAAR) only at ~10X coverage (Fig. 6). As reported in Table 3, all workflows reached ~ 100% precision at ≥40X depth of coverage excepted BioNumerics with ~ 98%.

**Table 3:** cgMLST precision (i.e mean percentage ± standard deviation) of the workflows BIGSdb (*n =* 420), INNUENDO (*n =* 336), GENPAT (*n =* 420), SeqSphere (*n =* 420), BioNumerics (*n =* 420) and MentaLiST (*n =* 420) according to targeted read (Dr) and kmer (Dk) depth (X) from BBNorm downsampling (read length *R =* 150 and kmer size K = 30) of *Listeria monocytogenes* paired-end reads from reference genomes ATCC19114, ATCC19115 and ATCCBAA679. The cgMLST schema harbors 1748 loci. NA means not applicable: Because of internal firewall, the INNUca assembler integrated into the cgMLST workflow INNUENDO cannot not perform assemblies of paired-end reads with read depth of coverage of 20X (*n =* 42) and 10X (*n =* 42)

Reference	Targeted depth of coverage	BIGSdb	INNUENDO	GENPAT	SeqSphere	BioNumerics	MentaLiST
**ATCC19114**	**Dr100-Dk75**	99.71 ± 0.02	99.71 ± 0.02	99.71 ± 0.02	99.71 ± 0.02	97.79 ± 0.04	99.71 ± 0.02
	**Dr90-Dk68**	99.71 ± 0.02	99.71 ± 0.02	99.71 ± 0.02	99.71 ± 0.02	97.80 ± 0.03	99.71 ± 0.02
	**Dr80-Dk60**	99.71 ± 0.02	99.71 ± 0.02	99.71 ± 0.02	99.71 ± 0.02	97.80 ± 0.04	99.71 ± 0.02
	**Dr70-Dk53**	99.71 ± 0.02	99.71 ± 0.02	99.71 ± 0.02	99.71 ± 0.02	97.80 ± 0.03	99.71 ± 0.02
	**Dr60-Dk45**	99.71 ± 0.02	99.71 ± 0.02	99.71 ± 0.02	99.71 ± 0.02	97.80 ± 0.04	99.71 ± 0.02
	**Dr50-Dk38**	99.71 ± 0.02	99.71 ± 0.02	99.71 ± 0.02	99.71 ± 0.02	97.79 ± 0.06	99.70 ± 0.02
	**Dr40-Dk31**	99.71 ± 0.02	99.71 ± 0.02	99.71 ± 0.02	99.71 ± 0.02	97.78 ± 0.06	99.54 ± 0.12
	**Dr30-Dk23**	99.71 ± 0.02	99.71 ± 0.02	99.71 ± 0.02	99.71 ± 0.02	97.69 ± 0.10	93.77 ± 0.82
	**Dr20-Dk16**	99.71 ± 0.02	NA	99.71 ± 0.02	99.71 ± 0.02	96.87 ± 0.22	36.67 ± 1.70
	**Dr10-Dk8**	99.44 ± 0.13	NA	99.51 ± 0.02	99.51 ± 0.09	69.63 ± 2.34	0.07 ± 0.07
**ATCC19115**	**Dr100-Dk75**	100 ± 0.00	99.83 ± 0.00	99.83 ± 0.00	100 ± 0.00	99.99 ± 0.02	100 ± 0.00
	**Dr90-Dk68**	100 ± 0.00	99.83 ± 0.00	99.83 ± 0.00	100 ± 0.00	99.99 ± 0.03	100 ± 0.00
	**Dr80-Dk60**	100 ± 0.00	99.83 ± 0.00	99.83 ± 0.00	100 ± 0.00	99.99 ± 0.03	100 ± 0.00
	**Dr70-Dk53**	100 ± 0.00	99.83 ± 0.00	99.83 ± 0.00	100 ± 0.00	100 ± 0.02	100 ± 0.00
	**Dr60-Dk45**	100 ± 0.00	99.83 ± 0.00	99.83 ± 0.00	100 ± 0.00	100 ± 0.00	100 ± 0.02
	**Dr50-Dk38**	100 ± 0.00	99.83 ± 0.00	99.83 ± 0.00	100 ± 0.00	99.99 ± 0.02	100 ± 0.02
	**Dr40-Dk31**	100 ± 0.00	99.83 ± 0.00	99.83 ± 0.00	100 ± 0.00	99.99 ± 0.03	99.85 ± 0.11
	**Dr30-Dk23**	100 ± 0.00	99.83 ± 0.00	99.83 ± 0.00	100 ± 0.00	99.91 ± 0.09	93.76 ± 0.84
	**Dr20-Dk16**	100 ± 0.00	NA	99.83 ± 0.00	100 ± 0.00	99.15 ± 0.26	37.04 ± 2.01
	**Dr10-Dk8**	99.77 ± 0.14	NA	99.64 ± 0.11	99.78 ± 0.13	72.41 ± 1.40	0.09 ± 0.05
**ATCCBAA679**	**Dr100-Dk75**	100 ± 0.02	99.82 ± 0.02	99.82 ± 0.02	100 ± 0.02	99.98 ± 0.04	99.96 ± 0.03
	**Dr90-Dk68**	100 ± 0.02	99.82 ± 0.02	99.82 ± 0.02	100 ± 0.02	99.99 ± 0.02	99.95 ± 0.03
	**Dr80-Dk60**	100 ± 0.02	99.82 ± 0.02	99.82 ± 0.02	100 ± 0.02	99.98 ± 0.04	99.95 ± 0.02
	**Dr70-Dk53**	100 ± 0.02	99.82 ± 0.02	99.82 ± 0.02	100 ± 0.02	99.97 ± 0.04	99.94 ± 0.03
	**Dr60-Dk45**	100 ± 0.02	99.82 ± 0.02	99.82 ± 0.02	99.99 ± 0.02	99.97 ± 0.03	99.95 ± 0.03
	**Dr50-Dk38**	100 ± 0.02	99.82 ± 0.02	99.82 ± 0.02	100 ± 0.02	99.97 ± 0.04	99.94 ± 0.04
	**Dr40-Dk31**	100 ± 0.02	99.82 ± 0.02	99.82 ± 0.02	100 ± 0.02	99.98 ± 0.04	99.81 ± 0.08
	**Dr30-Dk23**	100 ± 0.02	99.82 ± 0.02	99.82 ± 0.02	100 ± 0.02	99.92 ± 0.05	94.19 ± 1.02
	**Dr20-Dk16**	100 ± 0.02	NA	99.82 ± 0.02	100 ± 0.02	99.09 ± 0.25	37.18 ± 1.77
	**Dr10-Dk8**	99.69 ± 0.14	NA	99.56 ± 0.10	99.75 ± 0.10	72.49 ± 2.33	0.13 ± 0.06

### Clustering of cgMLST profiles

**Fig. 7:**
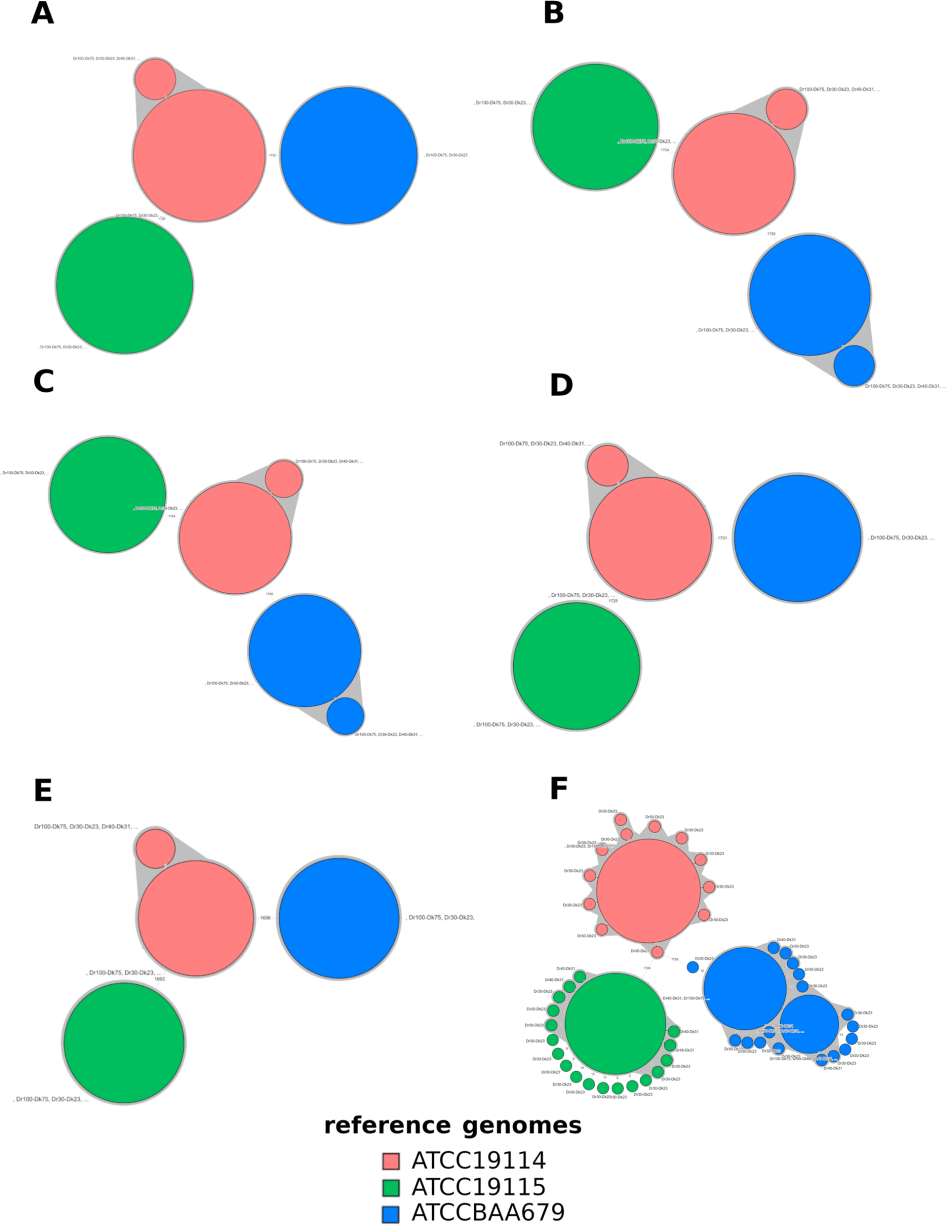
Minimum spanning trees (MSTs) representing the impact on clustering of cgMLST workflows BIGSdb (A: n = 339), INNUENDO (B: *n =* 339), GENPAT (C: *n =* 339), SeqSphere (D: *n =* 339), BioNumerics (E: *n =* 339) and MentaLiST (F: *n =* 339), of *Listeria monocytogenes* reference genomes (i.e. ATCC19114, ATCC19115 and ATCCBAA679) and targeted depth of coverage (Dr: 30X, 40X, 50X, 60X, 70X, 80X, 90X and 100X) prepared according to kmer depth (Dk): 8X, 15X, 23X, 30X, 38X, 45X, 52X, 60X, 67X, 75X) setting of BBNorm (read length *R =* 150 and kmer size K = 30) from downsampled paired-end reads (i.e. 2x150bp). The MSTs were built with BioNumerics ignoring missing data. The MST clusters of at least two genomes, one node and allele differences ≤7, were highlighted in grey

Minimum spanning tree (MST)-based clustering showed that the minimum depth of coverage of 40X consistently grouped the cgMLST profiles from each reference genomes into clusters with up to 7 pairwise allele differences (Fig. 7A-Fig. 7F). Below 40X, cluster discrepancies were identified for each cgMLST workflows (Additional file 8A-Additional file 8F). The major increase of pairwise allele differences according to decreasing of targeted depth was observed with MentaLiST (Additional file 8F), and to a lesser extent with BIGSdb (Additional file 8A), INNUENDO (Additional file 8B), GENPAT (Additional file 8C), SeqSphere (Additional file 8D) and BioNumerics (Additional file 8E). The effect of downsampling on MST-clustering was observed at 10X depth of coverage for all workflows (Additional file 8A-Additional file 8F) excepted MentaLiST that poorly clustered profiles from reads downsampled at ≤40X.

## Discussion

Internationally accepted validation of cgMLST typing workflows contributes to enhance routine surveillance of bacterial pathogens [21] by promoting the application of standards and benchmarking data sets [17]. Here, we focused on cgMLST precision and completeness between workflows rather than overall accuracy as the latter would refer to the ability to call the “right” alleles based on commonly assumed reference alleles. The comparison between different cgMLST workflows based on accuracy is hampered by the absence of a common strategy for definition of alleles, due to cgMLST approaches (i.e. assembly-based [12, 14–17] or -free [13, 18, 19], or combination of both [20]), as well as implemented algorithmic steps and related parameters (e.g. BLAST-based or -free algorithms, BLASTN or BLASTP, detection of open reading frames (ORFs) before BLAST step, coverage and identity of aligned sequences [12–20]).

### Allele differences between cgMLST workflows

In the present study, we did not assess the cgMLST precision with schemes presenting missing alleles because it would have decreased the completeness and precision of all cgMLST workflows, while minimizing differences of completeness and precision observed between these workflows. The allele differences observed between cgMLST workflows (Fig. 2) are induced by algorithmic differences of the definition of alleles and reflect the impossibility of direct comparisons of cgMLST profiles generated by different workflows (i.e. accuracy), delaying the multi-centers surveillance of strain variants. In the present study, the cgMLST allele calling of six workflows was assessed, using the 1748-loci *L. monocytogenes* schema [24]. All workflows successfully detected ~ 100% loci of the schema in the reference circular genomes with up to ~ 95% of common alleles showing exact match with alleles from the schema (Fig. 2). Overall, the main differences resulting from different profiles were either alleles uniquely found in a workflow, up to ~ 2% for SeqSphere or BioNumerics, or due to a different allele calling strategy, up to ~ 5% for INNUENDO and GENPAT (i.e. chewBBACA allele caller) (Fig. 2). While BIGSdb found an exact match in the reference schema for each allele as expected (because the schema was built based on this workflow), other cgMLST workflows (e.g. MentaLiST, INNUENDO and GENPAT) inferred new alleles (not presented in the reference schema) based on the implemented algorithms. These divergences hamper the comparison of profiles generated using different workflows, even when using a common scheme aiming at supporting interoperability of genomic data [44].

### In vitro parameters and cgMLST precision

Our overall results showed that in vitro parameters such as successive platings, replicates of DNA extraction and sequencing did not impact cgMLST precision, demonstrating that these wet-lab steps are very reproducible (*p* > 1.0 × 10^− 3^). Indeed, the improvements during several years of documentation, validation, quality check and quality monitoring of wet-lab steps, from growth of isolates to sequencing through DNA extraction and library preparation, allowed to obtain nowadays a stable and repeatable wet-lab process [45].

### In silico parameters and cgMLST precision

In contrast to the absence of effect from wet-lab parameters, the depth and breadth of coverages, as well as cgMLST workflows, tested reference strains and completeness (i.e. IAAS), were the main factors explaining cgMLST precision (i.e. IAAR), based on PCAs, GLMs and graphical confirmations. Indeed, the incapability to call alleles against schema (i.e. IAAS) impacts directly the number of identical alleles against reference genomes (i.e. IAAR), and consequently cgMLST precision (IAAR linearly correlated with IAAS; *p* = 3.7 × 10^− 6^). This underlines the necessity to keep cgMLST schemes regularly updated through synchronized systems (e.g. BIGSdb-*Lm* [24, 46] and chewieNS [47]). Recently proposed Hash-based nomenclature servers may circumvent the need of schema synchronization, and likely facilitate interlaboratories data comparability and sharing when confidentiality concerns apply (chewieSnake [48]). In terms of precision, we would expect chewieSnake having the same outputs than the workflow chewBBACA as both allele callers are based on the chewBBACA suite. However, such decentralized and nomenclature-free approach requires further developments to be integrated in global surveillance systems where common language and genotypes naming are essential. Indeed, when using the reference threshold of 7 pairwise allele differences, commonly used for WGS-based surveillance of *L. monocytogenes* to define clusters of isolates likely sharing an epidemiological link, the negative effect of incomparable profiles from different workflows became negligible, with all workflows leading to the same clusters when read depth of coverage was ≥40X (Fig. 7 and Additional file 8). These findings are consistent with previous studies on viruses [49] and bacteria [50], that did not observe improvement of the breadth of coverage above specific values of depth of coverage. Few studies recommended minimal depth of coverage for precise cgMLST typing of *L. monocytogenes* (40X with BIGSdb) [24, 28, 36], *Yersinia* (50X with BIGSdb) [42], *Mycoplasma* (47X with SeqSphere) [38], *Campylobacter*, *Chlamydia*, *Neisseria* and *Streptococcus* (20X with STing) [13]. For the first time in the present study, we recommend 40X as a suitable read depth of coverage for the highest cgMLST precision across 6 different assembly-based and -free workflows. This recommendation of minimal depth of coverage for precise cgMLST typing has been defined based on Illumina short reads (i.e. NextSeq) sequencing. Other short reads (IonTorrent) and long reads (PacBio SMRT and Oxford Nanopore) sequencing technologies may require higher depth of coverage than Illumina to reach similar quality of base calling, independently of GC-content and repeated region biases which are inherent in sequencing technologies based on short reads [51–53].

### Performances of assembly-based and -free cgMLST workflows

Among the 6 compared cgMLST workflows, BIGSdb, INNUENDO, GENPAT and SeqSphere did not show obvious effect of depth of coverage on precision contrary to BioNumerics and MentaLiST (Table 3). In particular, BIGSdb and SeqSphere performed well also at very low coverage values. This is probably due to refinement steps of assembly pipelines used for the workflow BIGSdb (i.e. fq2dna) and SeqSphere (i.e. average quality > 30 with a window of 20 bases), as well as similar allele definitions between BIGSdb and SeqSphere (i.e. BLASTN; nucleotide identity > 70%; coverage > 70%). In contrast, BioNumerics and MentaLiST were poorly precise for depth of coverage ≤30X and 40X (Fig. 6B) according to PCAs (Fig. 4) and GLMs (Table 2). Differences of precision between the cgMLST workflows are consequently induced by their respective de novo assemblers and/or allele callers. Even though MentaLiST requires more reads to achieve adequate precision compared to the assembly-based workflows, its precision is slightly impacted by tested reference genomes for high read depth of coverage (i.e. ≤ 30X and 40X) (Fig. 6B). This result highlights that MentaLiST precision is overall less impacted by in vitro and in silico parameters compared to assembly-based workflows, whose precision also depend on de novo assembly. Further comparisons with other assembly-free cgMLST workflows would confirm the supposed absence of strain effect on precision observed with MentaLiST [54]. However, MentaLiST outperformed other workflows in terms of percentage of correct allele predictions for cgMLST in a recent benchmarking of different assembly-free approaches [13]. Here we observed that both assembly-free and -based cgMLST workflows reach ~ 100% of identical allele predicted in the processed reads with coverage ≥40X compared to reference circular genomes.

### Performances of assembly-based cgMLST workflows

The decrease of cgMLST precision from assembly-based workflows may reflect the fragmentation of de novo assembly potentially induced by the GC bias [55] and/or and repetitive regions [56] (Additional file 4). This was particularly evident for BioNumerics workflow where the decreasing of cgMLST precision (i.e. IAAR) was linearly correlated with high amount of N100 through GLMs (*p* = 2.0 × 10^− 16^). This is probably induced by the absence of assembly refinement steps and/or an old version of SPAdes implemented in BioNumerics, in comparison with the other workflows (Table 4) [57]. In this study, no linear correlations between cgMLST precision and GC%, or cgMLST precision and duplication ratio were identified. Nevertheless, significant differences were observed (Wilcoxon rank sum tests: *p* < 2.2 × 10^− 16^) between GC% of references genomes draft assemblies (38.081 ± 0.007% for ATCC19114, 37.879 ± 0.006% for ATCC19115 and 37.865 ± 0.006% for ATCCBAA679), while duplication ratios were not significantly different (Wilcoxon rank sum tests: *p* > 1.5 × 10^− 2^) between these references genomes draft assemblies (1.0001 ± 0.0003 for ATCC19114, 1.00018 ± 0.0003 for ATCC19115 and 1.0000 ± 0.0008 for ATCCBAA679). Other statistical approaches would be necessary to test non-linear correlations [63, 64] between cgMLST precision and assembly metrics.

**Table 4:** License type, as well as de novo assembly and allele calling pipelines recommended by developers of cgMLST workflows compared in the present study to assess precision of *Listeria monocytogenes* cgMLST typing. N/A stands for not applicable

cgMLST workflow (version)	License type	Recommended assembly pipeline (version)	Recommended allele calling pipeline (strategy or version)	Reference
BIGSdb (N/A)	open source	AlienTrimmer (2.0)-, Musket (1.1)-and SPAdes (3.15.0)-based fq2dna (21.06)	BLASTN-based BIGSdb (alignment)	[24]
INNUENDO (N/A)	open source	Trimmomatic (0.36)-, Pilon (1.18)- and SPAdes (3.9.0)- based INNUca (4.2.2)	Prodigal- (ORF discovery) and BLASTP-based (alignment) chewBBACA (2.6.0)	[14, 49]
GENPAT (N/A)	open source	Trimmomatic (0.36)- and SPAdes (3.11.1)-based pipeline	Prodigal- (ORF discovery) and BLASTP-based (alignment) chewBBACA (2.6.0)	[14, 57, 58]
SeqSphere (6.0.2)	commercial	FastQC (0.11.7)- and SPAdes (3.11.1)-based pipeline	BLASTN-based SeqSphere (alignment)	[12, 59, 67]
Bionumerics (7.6.3)	commercial	SPAdes (3.7.1)-based pipeline	BLASTN-based assembly-based and -free algorithms (alignments)	[17, 20, 61, 62]
MentaLiST (1.0.0)	open source	N/A (i.e. assembly free)	stringMLST principle-based MentaLiST (kmer counting)	[13, 19]

### Future analytical prospects

The analytical approach (Fig. 1) here applied to *L. monocytogenes* can be easily fine-tuned for the analysis other bacterial species and taxa, assuming that a species-specific cgMLST scheme is established.

In the present study, the read depth of coverage was identified as one of the most impactful parameters on cgMLST precision. We thus proposed a minimal read depth of coverage of 40X for precise cgMLST typing and consistent MST clustering. We did not assess an upper limit of read depth but we showed that increasing the sequencing depth up to 100X did not effectively improve cgMLST precision. Sequencing at very high depth of coverage may promote errors on the assembly graph and confuse error correction algorithms, in addition to increase the computational burden [65]. Further studies may be needed to assess precision at higher coverage, yet 100X is enough high for *L. monocytogenes* cgMLST typing. Indeed, bacterial genomes sequences deposited in public databases (e.g. RefSeq, independently of the considered assembly surveillance project) are mostly generated at ≤100X sequencing depth (range: 30-150X) [66].

Our main goal here was to provide guidance concerning the “standalone” solutions that can be adopted today for assembly and allele calling following developers’ recommendations. Our results suggests that the assembly pipelines may impact the cgMLST precision to a greater extent than the allele calling pipelines. This hypothesis should be further confirmed assessing the impact of allele callers on cgMLST precision pipeline. However, results from Lüth et al. (2021) showed a ~ 100% correlation between matrices of cgMLST profile distances providing identical *L. monocytogenes* assemblies to different allele callers (e.g. Ridom SeqSphere versus chewBBACA) [46].

To foster interoperability between the tested cgMLST solutions, the impact of different allele calling settings on cgMLST precision and nonidentical calls (i.e. missing data, partial alleles and new alleles) should also be investigated. In view of the main differences between the cgMLST allele calling algorithms, such studies should assess settings, such as BLASTN nucleotide identity, BLAST coverage, word size (i.e. BIGSdb, SeqSphere, BioNumerics), allele size threshold, minimum BLASTP score ratio (i.e. chewBBACA implemented in GENPAT and INNUENDO), mutation threshold and kmer threshold (i.e. MentaLiST).

The definition of new alleles is not centralized between allele calling pipelines. This inevitably leads to a drift of allele identifiers in the scheme adopted by each system and consequently hinders profiles’ comparability and communication on *L. monocytogenes* genotypes across laboratories. A common effort of developers, curators and users of such cgMLST systems will allow the implementation of novel functionalities (e.g. application programming interfaces, nomenclature mapping) to ensure that an universal language is adopted by the scientific community.

## Conclusion

cgMLST precision was mainly impacted by the tested reference strains, cgMLST workflows, cgMLST completeness, as well as depth and breadth of coverage. Successive platings, DNA extraction and sequencing replicates did not show an impact on cgMLST precision. Overall loci detection was > 99% for assembly-free and assembly-based workflows and had no impact on cluster definitions, for read depth of coverage ≥40X. This study highlights the importance of high sequencing depth to ensure reproducibility of profiles in genomic surveillance and outbreak investigations.

## Material and methods

After a review about the cgMLST principles and approaches, the experimental plan, cgMLST workflows of interest, statistical analyses and confirmations of relevant parameters are presented successively.

### Review about cgMLST principles and approaches

The MLST method aims at assigning arbitrary numbers to each allele of a small set of DNA fragments from different loci (typically < 10 gene fragments with ~ 500 bp) presenting up- and downstream conserved sites for hybridization of forward and reverse oligonucleotides during PCR amplifications of housekeeping genes of interest [2]. The combination of these MLST allele numbers from a single strain allows assignment of a MLST sequence type (ST) already shared between laboratories or a new one [67]. The cgMLST is an extension of the MLST principle allowing screening of alleles from several hundreds of core genes. More precisely, after steps related to potential read trimming (usually with Trimmomatic [58]) and mandatory de novo assembly (usually with SPAdes [57]), the assembly-based cgMLST workflows include (i.e. chewBBACA [14]) or not (i.e. SeqSphere^+^ [12], MLSTar [15], BIGSdb-Pasteur [16], BioNumerics [17]) a step to detect open reading frames (ORFs) from drafts de novo assembly (i.e. Prodigal [68] implemented in chewBBACA [14]). Then, these assembly-based cgMLST workflows aligne alleles from schema to sequences from drafts de novo assembly (ORFs or not) based on the BLASTN (i.e. SeqSphere [12], MLSTar [15], BIGSdb-Pasteur [16], BioNumerics [17]) or BLASTP (i.e. chewBBACA [14]) algorithms [69], as well as different parameters related coverage and identity of aligned sequences. In addition, recently published assembly-free cgMLST workflows process reads independently of de novo assembly based on heuristic kmer mapping (i.e. KMA [18]) or counting and voting of kmers (MentaLiST [19] and STing [13]). Some cgMLST workflows may combine de novo assembly-free and -based allele calling (e.g. BioNumerics [20]). This review drove the selection of the 6 workflows of interest and related settings recommended by developers (BIGSdb, INNUENDO, GENPAT, SeqSphere, BioNumerics and MentaLiST), in order to cover the different genomics-based cgMLST typing approaches (Table 4).

### Experimental plan

The experimental plan was built to take into account a large range of in vitro and in silico parameters potentially explaining the cgMLST precision (i.e. identical alleles against reference circular genomes × 100 / 1748). The in vitro parameters include the tested reference genomes, successive platings, as well as replicates of DNA extraction and sequencing. The in silico parameters include the targeted read/kmer depth of coverage, read depth of coverage, read breadth of coverage, assembly metrics, cgMLST workflows and identified alleles against schema. For the sake of clarity, acronyms of this large set of parameters were defined in the section abbreviations.

### In vitro parameters of interest

Three *L. monocytogenes* strains and three original genomic DNA (gDNA) were obtained from the American Type Culture Collection Global Bioresource Center (ATCC: https://www.atcc.org): ATCC 19114 (cgMLST type L3-SL69-ST201-CT996, serotype 4a), ATCC 19115 (L1-SL2-ST145-CT375, serotype 4b) and ATCC BAA-679 (L2-SL9-ST35-CT637, serotype 1/2a), which corresponds to the reference EGD-e strain. The original gDNA of each of the three ATCC strains was sequenced in two different batches (i.e. *n* = 3 × 2 = 6 paired-end reads) (Fig. 1). The three ATCC strains were grown 5 and 10 times through successive plating (i.e. 4 and 9 platings, respectively), leading to two subcultures for each strain. Each of the subcultures was extracted three times, and each extract was then sequenced in two different batches (*n =* 3 × 2 × 3 × 2 = 36 paired-end reads) (Fig. 1). For bacterial culture, DNA was extracted using previously described procedures [70]. All gDNA samples were quantified by Qubit dsDNA HS Assay Kit using the Qubit fluorometer 2.0 (Thermo Fisher Scientific, Waltham, Massachusetts, United States). gDNA quality was estimated based on the Eppendorf BioSpectrometer® fluorescence (Eppendorf, Hamburg, Germany), whereas gDNA integrity was assessed using the Agilent 4200 TapeStation system (Agilent Technologies, Santa Clara, CA, United States). The sequencing libraries were prepared with 30 μl of Illumina DNA Prep kit and 100–500 ng of input gDNA. These libraries were sequenced with a NextSeq500 sequencer (Illumina). In total, a set of 42 paired-end reads (*n =* 3 × 2 + 3 × 2 × 3 × 2 = 42 paired-end reads) were produced to assess the impacts on cgMLST precision of in vitro parameters of interest: tested reference genomes, successive platings, as well as replicates of DNA extraction and sequencing (Fig. 1).

### In silico parameters of interest

The in silico parameters of interest include the targeted read/kmer depth of coverage, read depth of coverage, read breadth of coverage, assembly metrics, cgMLST workflows and identified alleles against schema. The number of reads, average Phred quality scores and percentages of Phred quality scores higher than 30 were checked for each 42 paired-end reads with FastQC (version 0.11.5) [79]. In addition, the absence of exogenous DNA contamination was confirmed with ConFindr (version 0.7.4) [41]. After quality assessment, downsampling of paired-end reads was performed with BBNorm (version February 13, 2020) in parallel with the estimation of depth and breadth of coverages of reads through BBMap-based mapping (version February 13, 2020) [42]. BBNorm-based downsampling was performed from paired-end reads (i.e. duplicated DNA samples of each 3 tested reference genomes) at 10 different kmer depth of coverage (Dk: 8X, 16X, 24X, 32X, 40X, 48X, 56X, 64X, 72X and 80X) fixing read length (*R =* 150) and kmer size (K = 30). Then, the corresponding read depth of coverage (Dr) measured with BBMap allowed estimation of the correlation with kmer depth of coverage (Dr = 1.3502 x Dk - 0.2923; R2 = 99.98%; *n =* 60) based on the ‘stats’ R library [72]. After this standard curve building, the setting of kmer depth of coverage (Dk: 8X, 15X, 23X, 30X, 38X, 45X, 52X, 60X, 67X, 75X) during another BBNorm-based downsampling (i.e. argument ‘target’) allowed preparation of 420 paired-end reads with different read depth of coverage (Dr: 10X, 20X, 30X, 40X, 50X, 60X, 70X, 80X, 90X and 100X). Finally, the high read breadth of coverage and expected read depth of coverage (Dr) of the 420 prepared paired-end reads were double checked independently with BBMap [42], and the INNUca (version 4.2.2) [49] internal module based on Bowtie2 (version 2.2.9) [73] and Samtools (version 1.3.1) [74]. In total, 420 paired-end reads were produced to assess the impacts on cgMLST precision of in silico parameters (Fig. 1). Following de novo assembly steps recommended by developers detailed below, the 420 paired-end reads were processed through the six cgMLST workflows of interest (*n =* 6 × 10 x [3 × 2 + 3 × 2 × 3 × 2] = 2520 cgMLST results) (Fig. 1). Then, de novo assembly metrics of the assembly-based cgMLST workflows were assessed with Quast (version 5.0.2) [75] and combined with MultiQC (version 1.9) [76] (*n* = 5 × 10 x [3 × 2 + 3 × 2 × 3 × 2] = 2100 quality results assessing 48 assembly metrics) (Fig. 1).

### cgMLST workflows of interest

Six different cgMLST workflows were tested: BIGSdb [24], INNUENDO [14, 49], GENPAT [14, 57, 58], SeqSphere [12, 59, 67], BioNumerics [17, 20, 61, 62] and MentaLiST [19] (Fig. 1). The open-source workflows (MentaLiST, INNUENDO and GENPAT), based on Docker images (version 19.03.4) (https://www.docker.com/) which are hosted in the in-house GENPAT system (IZSAM, Italy), and commercial workflows (BioNumerics and SeqSphere) were executed in IZSAM (Italy). The workflow GENPAT corresponds to the in-house cgMLST workflow implemented in the GENPAT system (IZSAM, Italy). The open-source workflow BIGSdb was executed using the genomic taxonomy platform of Institut Pasteur (France; https://bigsdb.pasteur.fr/). All cgMLST workflows included in the present study were assessed based on the same set of loci and alleles, using the *L. monocytogenes* schema of 1748 cgMLST loci [24] downloaded from BIGSdb-*Lm* [24, 46] on 8th March 2021.

#### BIGSdb

Paired-end reads were de novo assembled using fq2dna version 21.06 (https://gitlab.pasteur.fr/GIPhy/fq2dna; strategy B; default settings). The corresponding fq2dna pipeline consists of trimming and clipping of low-quality reads and adapters with AlienTrimmer (version 2.0) [77], sequencing error correction with Musket (version 1.1) [78], paired-end read merging with FLASh (version 1.2.11) [79], coverage homogenization with ROCK (version 1.9.3; https://gitlab.pasteur.fr/vlegrand/ROCK) [81, 82, 90], and de novo assembly with SPAdes (version 3.15.0) [57]. In brief, the paired-end reads were first pre-processed through deduplication, clipping, trimming (Phred score threshold: 15, minimum read length: 50 bp) and error correction. Second, two distinct sequence datasets were created for each paired-reads by merging or not the pre-processed paired-end reads. Third, the coverage depth of the two read datasets (i.e. merged or not) was homogenized to 60X (i.e. digital normalization procedure), and each of the two resulting subsets of paired-end reads was used to infer a de novo genome assembly. The most precise between the two assemblies was selected by maximizing the number of genes completely contained within assembled contigs (E-size) [83]. Finally, the selected assembly was used together with its corresponding paired-end reads to infer a genome coverage profile (GCP) (i.e. distribution of the number of assembled bases per sequencing depth value) [84]. Based on the coverage profile, sufficiently long (> 1000 bp) and significantly covered scaffold sequences were finally selected. Contigs smaller than 300 bp were ignored. Draft assemblies were uploaded in a dedicated project in BIGSdb-*Lm* (https://bigsdb.pasteur.fr/listeria) powered by the BIGSdb software (version 1.31.0) [46]. cgMLST allele calling [46] was performed therein based on the BLASTN algorithm [69], with minimum of 70% of nucleotide identity and 70% of coverage and word size of 10. The missing data (0) and mismatches (empty set) from BIGSdb were considered as nonidentical calls in the present study. For the record, the mismatches (empty set) correspond to potential new alleles which are quality-checked by the Institute Pasteur curator before designation of new identifiers.

#### INNUENDO

As proposed by the cross-sectoral platform for the integration of genomics in the surveillance of food-borne pathogens (INNUENDO), the cgMLST workflow INNUENDO was based on de novo assembly and allele calling using INNUca (version 4.2.2) [49] and chewBBACA (version 2.6.0; default setting) [14] pipelines, respectively. More precisely, the INNUca assembler performs successively read control with FastQC (version 0.11.5) [79], trimming with Trimmomatic (version 0.36; clipping 3:30:10:6; sliding window 5:20; leading 3; trailing 3; minimum length 55) [58], coverage estimation with the internal module based on Bowtie2 (version 2.2.9) [73] and Samtools (version 1.3.1) [74], de novo assembly with SPAdes (version 3.9.0, careful; only assembler: coverage cutoff 2; k 21,33,55,67 and 77) [57], pearl-based filtering of contigs presenting at least 200 bp, kmer coverage of 2 and CG content between 5.0 and 95.0% (version 0.9.10), and correction of draft assembly with Pilon (version 1.18) [85], as well as MLST assessment based on MLST (version 2.4) [16]. The default parameters of chewBBACA (including allele size threshold = 0.2, BLASTP score ratio ≥ 0.6 and the recommended prodigal training file Listeria_monocytogenes.trn: https://chewbbaca.online/stats [69]) were applied in the present study considering exact match with known alleles (encoded EXC) as identical calls, as well as new inferred allele (INF), locus not found (LNF), possible locus on the tip of contigs (PLOT), non-informative paralogous hits (NIPH), alleles larger (ALM) and smaller (ASM) than mode, as nonidentical calls.

#### GENPAT

The GENPAT workflow is constituted of the NGSmanager de novo assembly pipeline implemented in GENPAT and chewBBACA allele caller with identical setting and version described above (see INNUENDO) [14]. More precisely, the NGSmanager assembly pipeline performs read trimming with Trimmomatic (version 0.36; clipping 2:30:10; leading 25; trailing 25 sliding window 20:25 minimal length 36) [58], de novo assembly with SPAdes (version 3.11.1; only assembler; careful; −k 21, 33, 55 and 77) [57], and filtering of contigs lower than 200 bp with a homemade Python script AssemblyFilter.py (i.e. version 2.7.8). The chewBBACA-based definitions of identical and nonidentical calls of the GENPAT workflow were identical to those described above (see INNUENDO).

#### SeqSphere

A new task template was created in Ridom SeqSphere+ (version 6.0.2), so-called SeqSphere in the present study, by importing allele library constructed using *L. monocytogenes* 1748 loci schema of cgMLST alleles [25] downloaded from BIGSdb-Pasteur, as described above. The first allele of each target was indicated as a reference sequence (ref-seq) and ref-seq alignment gap penalty was set to default. In the default “Target QC Procedure”, the warnings were issued for alleles with breadth of coverage < 75% and read depth of coverage <5X, as well as in cases of frameshift detected in translatable target and consensus length varying by more than 6 triplets compared to the ref-seq. Moreover, ambiguities were not allowed in the target sequences. The target scan procedure was set according to the guidelines for *L. monocytogenes* cgMLST typing from the Institute Pasteur (https://bigsdb.pasteur.fr/listeria/cgMLST_guidelines.pdf) with the minimum required allele identity and minimum percentage aligned to re-seq of 70% [24]. The best matching allele was forced when multiple gene matches were identified. In order to assess the full workflow of Ridom SeqSphere+, the sequencing reads were assembled de novo using the integrated assembly pipeline. Briefly, the paired sequencing reads were quality-trimmed with FastQC (version 0.11.7) at 5′ and 3′ end until average quality was 30 in a window of 20 bases [79]. The trimmed reads were assembled with SPAdes using default settings (−-careful option enabled) [57]. The assembled scaffolds were scanned for the presence of targeted genes and the alleles were assigned using the established parameters. The unidentified (? (not found)) and new alleles (? (new)) from SeqSphere+ were considered as nonidentical calls in the present study.

#### BioNumerics

BioNumerics (Applied Maths NV: bioMérieux company, Sint-Martens-Latem, Belgium) offers a fully automated workflow for cgMLST, the so-called WGS tools plugin (version 7.6.3). By default, the WGS tools plugin (i.e. AWS environment) proposes assembly-based (i.e. BLASTN algorithm from de novo assembly [69]) and/or -free workflows (i.e. kmer-based detection of alleles from unassembled reads) [34, 46, 61]. The BioNumerics assembly-based workflow can detect new alleles in addition to allele calling, while the assembly-free workflow cannot identify new alleles (https://www.applied-maths.com/news/bionumerics-version-763-released). By default, the BioNumerics outputs of the free-assembly workflow correspond to cgMLST alleles identically identified by assembly-based and -free workflows, in addition to alleles identified only through assembly-free workflow. Consequently, the output of the assembly-based workflow alone (BioNumericsAB), or in combination with the assembly-free workflow (BioNumericsAF), were firstly compared to each other in the present study in order to compare secondly the most precise one to the other cgMLST workflow of interest. More precisely, the reads were assembled using SPAdes (version 3.7.1) implemented in BioNumerics (version 7.6.2) without specifying any parameter, then the sequences obtained were scanned with the “assembly-based calls” and “assembly-free calls” algorithms successively. The minimum similarity to call new alleles (i.e. 80%), kmer size (35 bases), minimum coverage (3X), minimum forward coverage (1X) and minimum reverse coverage (1X) were set following BioNumerics recommendations. The unidentified alleles from BioNumerics (labeled with a question mark ‘?’) were considered as nonidentical calls in the present study.

Even though few differences of identical alleles against reference circular genomes (IAAR) were observed at extended scales between the workflows BioNumericsAB and BioNumericsAF (Additional file 9A and Additional file 9B), the workflow BioNumericsAF identified significantly (Wilcoxon signed rank tests: *p* < 1 × 10^− 6^) more IAAR that the workflow BioNumericsAB for each targeted depth of coverage (Additional file 9C and Additional file 9D). Consequently, the BioNumerics workflow combining assembly-based and-free approaches was retained to be compared to the other cgMLST workflow. In the interests of simplification, this BioNumerics workflow combining assembly-based and-free approaches (i.e. BioNumericsAF) will be named BioNumerics workflow in the present study.

#### MentaLiST

Working directly with the raw paired-end reads, MentaLiST does not require prior genome assembly (i.e. de novo assembly or reference genome mapping) [19]. In brief, the workflow MentaLiST (version 1.0.0) implements the principle of kmer counting [54] and data compression to decrease dataset sizes and execution duration based on the construction of a coloured de Bruijn graph [87]. After assessment of all kmers present on the schema of alleles for each locus stored as a kmer hash map, all alleles that contain kmers from reads of a given sample will receive one vote, and the called alleles are those with the most votes for each locus [19]. The argument “--fasta” of MentaLiST was used to perform cgMLST of the three ATCC reference assemblies used in the present study. The default parameters of MentaLiST were applied in the present study considering multiple possible alleles (+) and partially covered alleles (−) as identical calls, as well as missing loci (0 or 0?) and new allele (N) as nonidentical calls.

### Statistical analyses

The differences of alleles between cgMLST workflows applied to reference circular genomes were represented through Edward’s Venn diagrams [88] built with jvenn (http://jvenn.toulouse.inra.fr/app/example.html) [89]. The results from paired-end read downsampling (Additional file 1), cgMLST typing (Additional file 2) and the parameters of interest (Fig. 1) were compiled into a single dataframe (Additional file 3) to perform statistical analyses. With the objective to explain the precision of cgMLST workflows, the amount of identical alleles against reference genomes (i.e. the parameter to explain, also called the response variable) was compared to several in vitro and in silico parameters of interest (i.e. the parameters potentially explaining the response variable, also called the explanatory variables) based on two independent statistical analyses, namely PCA and GLM. The PCA and GLM were selected because of their abilities to manage together categorical and numerical parameters. The in vitro parameters of interest include 4 categorical parameters (i.e. tested reference genomes, successive platings, as well as replicates of DNA extraction and sequencing). The in silico ones include 2 categorical (i.e. cgMLST workflows and targeted read/kmer depth of coverage) and 51 numerical parameters (i.e. read depth of coverage, read breadth of coverage, 48 assembly metrics and number of identified alleles against schema) (Additional file 3). The R-scripts dedicated to statistical analyses are available in GitHub (https://github.com/Nicolas-Radomski/DownsampledReads and https://github.com/Nicolas-Radomski/cgMLSTcomparison).

#### Principal component analyses

The exploratory PCAs aimed at increasing interpretability and minimizing information loss at the same time, by reducing the dimensional of the large dataset of numerical parameters through projection of data points on the first few principal components [90]. Two different PCAs were performed in the present study. The first PCA assessed the behavior of the response variable (i.e. the parameter to explain: IAAR) together with the explanatory variables corresponding to in silico numerical parameters of interest estimated through all assembly-based and assembly-free cgMLST workflows (i.e. the parameters potentially explaining the response variable: DEPTH, BREADTH and IAAS). The PCA was repeated excluding the assembly-free workflow MentaLiST (i.e. DEPTH, BREADTH, assembly metrics and IAAS) to additionally evaluate the impact of 48 assembly metrics on cgMLST precision for a total of 52 numerical parameters (i.e. DEPTH, BREADTH, 48 assembly metrics, IAAS and IAAR). For readability of the illustrations, these numerical parameters were grouped together according to PCA outcomes and only one parameter from each group was represented (Additional file 4). These PCAs were systematically performed in comparison to the in vitro and in silico categorical parameters of interest (i.e. tested reference genomes, successive platings, as well as replicates of DNA extraction and sequencing, targeted read/kmer depth of coverage and cgMLST workflows). These PCAs were performed with the ggplot2-based biplot R library [91] called “ggbiplot” (https://github.com/vqv/ggbiplot) requiring R libraries “usethis” and “devtools” [72].

#### Generalized linear models

Extending the concept of the linear regression model, the GLMs integrate link functions around the linear combinations of the explanatory variables in order to bypass the restriction to linearity from the linear models [92]. As described above concerning the PCAs, two different GLMs were performed in the present study. The first GLM aimed at explaining the response variable (i.e. the parameter to explain: IAAR) by explanatory variables corresponding to in vitro and in silico parameters of interest (i.e. numerical and categorical) estimated through all assembly-based and assembly-free cgMLST workflows (i.e. the parameters potentially explaining the response variable: tested reference genomes (REFERENCE), successive platings (PLATING), DNA extraction replicates (DNA), sequencing replicates (SEQUENCING), read depth of coverage (DEPTH), read breadth of coverage (BREADTH) and IAAS). Following the same design, the second GLM aimed at explaining the response variable by explanatory variables from assembly-based cgMLST workflows (i.e. the parameters potentially explaining the response variable: REFERENCE, PLATING, DNA, SEQUENCING, DEPTH, BREADTH, assembly metrics and IAAS). Before to perform these GLMs, the distributions of the response variable were assessed through statistical tests Shapiro-Wilk (Gaussian distribution), Chi-square (uniform distribution), two side Poisson (two side Poisson distribution), one side Poisson with upper hypothesis (one side Poisson distribution with upper hypothesis) and one side Poisson with lower hypothesis (one side Poisson distribution with upper hypothesis) implemented in the R library “stats” [72].

Including or excluding MentaLiST from the cgMLST comparison, the IAAR did not follow Gaussian (Shapiro-Wilk, *p* < 2.2 × 10^− 16^), uniform (Chi-square, p*p* < 2.2 × 10^− 16^), two side Poisson (two side Poisson, *p* < 2.2 × 10^− 16^) or one side Poisson with upper hypothesis (one side Poisson with upper hypothesis, *p* < 2.2 × 10^− 16^) distributions, in the favor of one side Poisson with lower hypothesis (one side Poisson with upper hypothesis, *p* = 1). The presence (including MentaLiST) and absence (excluding MentaLiST) of GLM overdispersions, implemented in the R library “AER” [93], allowed retainment of quasiPoisson- (dispersion test, *p* < 2.2 × 10–16^–16^ and alpha > 1) and Poisson- (dispersion test, *p =* 1 and alpha ≈ 1) distributions for GLMs, respectively, for the R function “glm” from the R library “stats” [72].

### Confirmations of parameters explaining the cgMLST precision

In order to confirm results from PCA- and GLMs-based statistical analyses, the parameters explaining the cgMLST precision (i.e. IAAR × 100 / 1748) were presented trough four-way figures, MST-based clustering and three-way tables.

#### Four-way figures

The IAAS (Additional file 6A-Additional file 6D) and IAAR (Additional file 6E-Additional file 6H) were presented according to the parameters of interest focusing on the parameters explaining the cgMLST precision through four-way figures with the R library “ggplot2” [91]. These four-way figures were prepared with y-axis presenting broad (i.e. extended scale) or narrow (i.e. restricted scale) range of units.

#### MST-based clustering

The cgMLST clustering was represented through MSTs according to parameters explaining the cgMLST precision. The cgMLST profiles from each workflow (Additional file 2) were used to build MSTs using Bionumerics software (version 7.6.3). Missing alleles calls were ignored in the MST differences calculations. The MST clusters containing at least two genomes and allele differences ≤7, were highlighted in grey.

#### Three-way tables

The cgMLST precision (i.e. IAAR × 100 / 1748) was presented according to parameters explaining it through three-way tables with the R functions ‘subset’ and ‘dcast’ from the R library “base” and “reshape2”, respectively [72].

## Supplementary Information

**Additional file 1 **Read depth (X) and breadth (%) coverages estimated with BBMap (version February 13, 2020) or INNUca (version 4.2.2) of *Listeria monocytogenes* paired-end reads from reference genomes ATCC19114, ATCC19115 and ATCCBAA679 (*n =* 420) downsampled at different targeted read (Dr: 10X, 20X, 30X, 40X, 50X, 60X, 70X, 80X, 90X and 100X) and kmer (Dk: 8X, 15X, 23X, 30X, 38X, 45X, 52X, 60X, 67X, 75X) depth (X) with BBNorm (read length *R =* 150 and kmer size K = 30). (TSV 65 kb)

**Additional file 2 **Standardized matrices of the cgMLST workflows BIGSdb (*n* = 423), INNUENDO (*n* = 339), GENPAT (*n =* 423), SeqSphere (*n =* 423), BioNumericsAB (*n =* 423), BioNumericsAF (*n =* 423) and MentaLiST (*n =* 423) applied to downsampled paired-end reads from 3 reference genomes of *Listeria monocytogenes* (i.e. ATCC19114, ATCC19115 and ATCCBAA679). The terms ATCC19114, ATCC19115 and ATCCBAA679 from the field FILE correspond to cgMLST profiles of the corresponded circular de novo assemblies from ATCC company. The empty sets represent mismatches. Because of internal firewall, the INNUca assembler integrated into the cgMLST workflow INNUENDO cannot not perform assemblies of paired-end reads with read depth of coverage of 20X (*n =* 42) and 10X (*n =* 42). (TSV 12262 kb)

**Additional file 3 **Standardized outcomes of the cgMLST workflows BIGSdb (*n* = 420), INNUENDO (*n* = 336), GENPAT (*n* = 420), SeqSphere (n = 420), BioNumerics (*n =* 420) and MentaLiST (*n =* 420) applied to downsampled paired-end reads from 3 reference genomes of *Listeria monocytogenes* (i.e. ATCC19114, ATCC19115 and ATCCBAA679) with associated de novo assembly parameters assessed with Quast (version 5.0.2) and MultiQC (version 1.9). The targeted read depth (Dr: 10X, 20X, 30X, 40X, 50X, 60X, 70X, 80X, 90X and 100X) were prepared according to kmer depth (Dk): 8X, 15X, 23X, 30X, 38X, 45X, 52X, 60X, 67X, 75X) setting of BBNorm (read length *R =* 150 and kmer size K = 30). Because of internal firewall, the INNUca assembler integrated into the cgMLST workflow INNUENDO cannot not perform assemblies of paired-end reads with read depth of coverage of 20X (*n =* 42) and 10X (*n =* 42). (TSV 1207 kb)

**Additional file 4 **Principals component analyses (PCAs) of the numerical parameters C1000, C10000, DR, GC, IAAR, IAAS, ID100, L50, LA50, LA, LMA, MA, N50, NA50, DEPTH, BREADTH, SQEM, SQLM, TL1000, TL10000, UACP and UAMC (defined in the section abbreviations) according to the categorical parameters “reference genome” (A), “successive platings” (B), “DNA extraction replicate” (C), “sequencing replicate” (D), “targeted depth” (E), “cgMLST workflows” (F), including assembly-based cgMLST workflows BIGSdb (*n* = 420), INNUENDO (*n =* 336), GENPAT (*n =* 420), SeqSphere (*n =* 420) and BioNumerics (*n =* 420) applied to downsampled paired-end reads from 3 reference genomes of *Listeria monocytogenes* (i.e. ATCC19114, ATCC19115 and ATCCBAA679). The PCA parameters C0-C1000-C5000-C10000-C25000-C50000, GC-TL0-TL1000-TL5000-TL10000-TL25000-TL50000-TL-TAL-MACL, N50-NG50-N75-NG75-SQEM-NA50-NGA50-NA75-NGA75-LA, L50-LG50-L75-LG75, LA50-LGA50-LA75-LGA75, DEPTH-GF, LMA-UAL-MM100-SQLM, DR-N100-UAC and MA-MAC were overlapped and are consequently not presented together.

**Additional file 5 **Coefficients (Coef.) of the generalized linear models (GLMs with Poisson distribution and without overdispersion) comparing the parameters “identical alleles against reference circular genomes” (IAAR) with the parameters of interest “reference genome” (REFERENCE), “successive platings” (PLATING) (B), “DNA extraction replicate” (DNA), “sequencing replicate” (SEQUENCING), “read depth” (DEPTH), “read breadth” (BREADTH), assembly parameters (defined in the section abbreviations: C0, C1000, C10000, C25000, C5000, C50000, DR, GC, GF, ID100, L50, L75, LA, LA50, LA75, LC, LG50, LG75, LGA50, LGA75, LMA, MA, MAC, MACL, MM100, N100, N50, N75, NA50, NA75, NG50, NG75, NGA50, NGA75, SQEM, SQLM, TAL, TL, TL0, TL1000, TL10000, TL25000, TL5000, TL50000, UAC, UACP, UAL, UAMC), cgMLST workflows (WORKFLOW) together (A) and cgMLST workflows independently including BIGSdb (B: *n* = 420), INNUENDO (C: *n =* 336), GENPAT (D: *n =* 420), SeqSphere (E: *n =* 420) and BioNumerics (F: *n =* 420), applied to downsampled paired-end reads from 3 reference genomes of *Listeria monocytogenes* (i.e. ATCC19114, ATCC19115 and ATCCBAA679). Few parameters are not defined because of singularities.

**Additional file 6 **Box-plots representing the impact of downsampled paired-end reads (i.e. 2x150bp) of reference genomes of *Listeria monocytogenes* (i.e. ATCC19114, ATCC19115 and ATCCBAA679), on cgMLST outcomes (BIGSdb: *n =* 420, INNUENDO: *n =* 336, GENPAT: *n =* 420, SeqSphere: *n =* 420, BioNumerics: *n =* 420 and MentaLiST: *n =* 420), including identified alleles against schema (A, B, C, D) or identical alleles against reference circular genomes at extended (E, F, G, H) or restricted (I, J, K, L) scales, according to reference genomes (A, E, I), successive platings (B, F, J), DNA extraction replicate (C, G, K) and sequencing replicate (C, H, L). The targeted read depth (Dr: 10X, 20X, 30X, 40X, 50X, 60X, 70X, 80X, 90X and 100X) were prepared according to kmer depth (Dk): 8X, 15X, 23X, 30X, 38X, 45X, 52X, 60X, 67X, 75X) setting of BBNorm (read length *R =* 150 and kmer size K = 30). Because of internal firewall, the INNUca assembler integrated into the cgMLST workflow INNUENDO cannot not perform assemblies of paired-end reads with read depth of coverage of 20X (*n =* 42) and 10X (*n =* 42).

**Additional file 7 **Box-plots representing the impact of downsampled paired-end reads (i.e. 2x150bp) of *Listeria monocytogenes* on unidentified alleles against schema at extended (A) or restricted (B) scales, according to reference genomes (i.e. ATCC19114, ATCC19115 and ATCCBAA679) and cgMLST workflows including BIGSdb (*n =* 420), INNUENDO (*n =* 336), GENPAT (*n =* 420), SeqSphere (*n =* 420), BioNumerics (*n =* 420) and MentaLiST (*n =* 420). The targeted read depth (Dr: 10X, 20X, 30X, 40X, 50X, 60X, 70X, 80X, 90X and 100X) were prepared according to kmer depth (Dk): 8X, 15X, 23X, 30X, 38X, 45X, 52X, 60X, 67X, 75X) setting of BBNorm (read length *R =* 150 and kmer size K = 30). Because of internal firewall, the INNUca assembler integrated into the cgMLST workflow INNUENDO cannot not perform assemblies of paired-end reads with read depth of coverage of 20X (*n =* 42) and 10X (*n =* 42).

**Additional file 8 **Minimum spanning trees (MSTs) representing the impact on clustering of cgMLST workflows BIGSdb (A: *n =* 423), INNUENDO (B: *n* = 339), GENPAT (C: *n =* 423), SeqSphere (D: *n =* 423), BioNumerics (E: *n =* 423) and MentaLiST (F: *n =* 423), of *Listeria monocytogenes* reference genomes (i.e. ATCC19114, ATCC19115 and ATCCBAA679 on the left of each workflow) and targeted depth of coverage (i.e. on the right of each workflow) from downsampled paired-end reads (i.e. 2x150bp). The MSTs were built with BioNumerics ignoring missing data. The MST clusters of at least two genomes, one node and allele differences ≤7, were highlighted in grey. The targeted read depth (Dr: 10X, 20X, 30X, 40X, 50X, 60X, 70X, 80X, 90X and 100X) were prepared according to kmer depth (Dk): 8X, 15X, 23X, 30X, 38X, 45X, 52X, 60X, 67X, 75X) setting of BBNorm (read length *R =* 150 and kmer size K = 30). Because of internal firewall, the INNUca assembler integrated into the cgMLST workflow INNUENDO cannot not perform assemblies of paired-end reads with read depth of coverage of 20X (*n =* 42) and 10X (*n =* 42).

**Additional file 9 **Box-plots representing the impact of downsampled paired-end reads (i.e. 2x150bp), at extended (A and B) or restricted (C and D) scales of identical alleles against reference circular genomes, spiting (A and C) or merging (B and D) reference genomes of *Listeria monocytogenes* (i.e. ATCC19114, ATCC19115 and ATCCBAA679), on cgMLST outcomes from the assembly-based workflow alone (BioNumericsAB: *n =* 420), or in combination with the assembly-free workflow implemented in BioNumerics (version 7.6.2) (BioNumericsAF: *n =* 420).

## Data Availability

The paired-end reads are available in the European Nucleotide Archive (ENA) under the BioProject PRJEB45560 (https://www.ebi.ac.uk/ena/browser/view/PRJEB45560).

## Abbreviations

ALM: Alleles larger than mode
ASM: Alleles smaller than mode
ATCC: American Type Culture Collection Global Bioresource Center
BIGSdb-*Lm*: Bacterial Isolate Genome Sequence database (BIGSdb) dedicated to *L. monocytogenes*
BREADTH: Read breadth of coverage
C0: Contigs > 0 bp
C1000: Contigs > 1 000 bp
C5000: Contigs > 5 000 bp
C10000: Contigs > 10 000 bp
C25000: Contigs > 25 000 bp
C50000: Contigs > 50 000 bp
cgMLST: Core genome multi-locus sequence typing
CRBIP: Biological Resources Center of the Institut Pasteur
DEPTH: Read depth of coverage
DNA: DNA extraction replicate
DR: Duplication ratio
DrDk: Targeted read (Dr) and kmer (Dk) depth
E-size: Number of genes completely contained within assembled contigs
EXC: Exact match with known alleles
GC: GC%
GCP: Genome coverage profile
GENPAT: Whole Genome Sequencing of microbial pathogens: data-base and bioinformatics analysis
GF: Genome fraction
GLM: Generalized linear model
IAAR: Identical alleles against reference circular genomes
IAAS: Identified alleles against schema
ID100: Indels per 100 kb
INF: New inferred allele
INNUENDO: Cross-sectoral platform for the integration of genomics in the surveillance of food-borne pathogens
IT: Information technology
K: Kmer size
L50: Smallest number of contigs whose length sum makes up 50% of the total genome length
L75: Smallest number of contigs whose length sum makes up 75% of the total genome length
LA: Largest alignment
LA50: Smallest number of aligned blocks whose length sum makes up 50% of the total genome length
LA75: Smallest number of aligned blocks whose length sum makes up 75% of the total genome length
LC: Largest contig
LG50: Smallest number of contigs whose length sum makes up 50% of the total reference genome length
LG75: Smallest number of contigs whose length sum makes up 75% of the total reference genome length
LGA50: Smallest number of aligned blocks whose length sum makes up 50% of the total reference genome length
LGA75: Smallest number of aligned blocks whose length sum makes up 75% of the total reference genome length
LMA: Local misassemblies
LNF: Locus not found
MA: Misassemblies
MAC: Misassembled contigs
MACL: Misassembled contigs length
MIAAR: Misidentified alleles against reference
MLST: Multi-locus sequence typing
MM100: Mismatches per 100 kb
MST: Minimum spanning tree
N: Any base
N100: N per 100 kb
N50: Sequence length of the shortest contig at 50% of the total genome length
N75: Sequence length of the shortest contig at 75% of the total genome length
NA50: Sequence length of the shortest aligned blocks at 50% of the total genome length
NA75: Sequence length of the shortest aligned blocks at 75% of the total genome length
NG50: Sequence length of the shortest contig at 50% of the total reference genome length
NG75: Sequence length of the shortest contig at 75% of the total reference genome length
NGA50: Sequence length of the shortest aligned blocks at 50% of the total reference genome length
NGA75: Sequence length of the shortest aligned blocks at 75% of the total reference genome length
NIPH: Non-informative paralogous hits
ORFs: Open reading frames
PCA: Principal component analysis
PLATING: Successive platings
PLOT: Possible locus on the tip of contigs
R: Read length
REFERENCE: Tested reference genomes s
SEQUENCING: Sequencing replicate
SNV: Single nucleotide variant
SQEM: Scaffold gap extensive misassembly
SQLM: Scaffold gap local misassembly
ST: Sequence type
TAL: Total aligned length
TL: Total length
TL0: Total length>0bp
TL1000: Total length > 1000 bp
TL5000: Total length > 5 000 bp
TL10000: Total length > 10 000 bp
TL25000: Total length > 25 000 bp
TL50000: Total length > 50 000 bp
UAC: Unaligned contigs
UACP: Unaligned contigs partial
UAL: Unaligned length
UAMC: Unaligned and misassembly contigs
UIAAS: Unidentified alleles against schema
wgMLST: Whole genome MLST
WGS: Whole genome sequencing
