# Supplementary figures and images for: In vitro and in silico parameters for precise cgMLST typing of Listeria monocytogenes
Source: BMC Genomics. 2022 Mar 26;23:235. doi: 10.1186/s12864-022-08437-4 (PMC8961897; doi:10.1186/s12864-022-08437-4)

**A**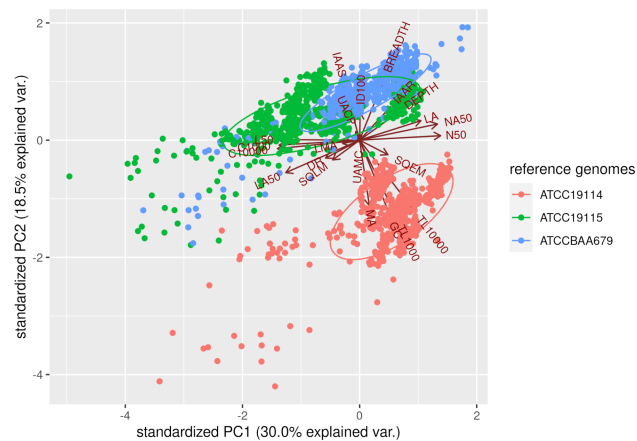**B**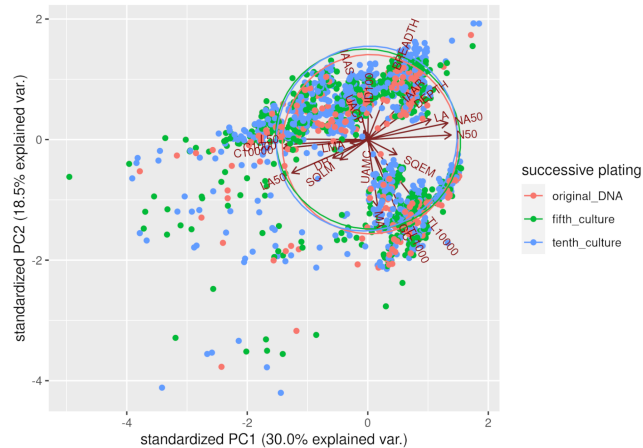**C**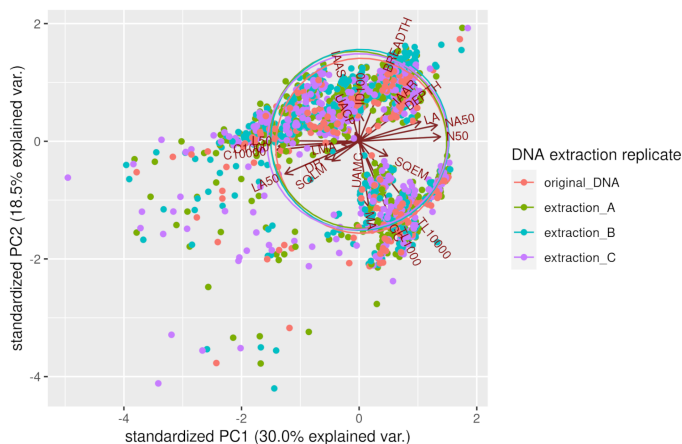**D**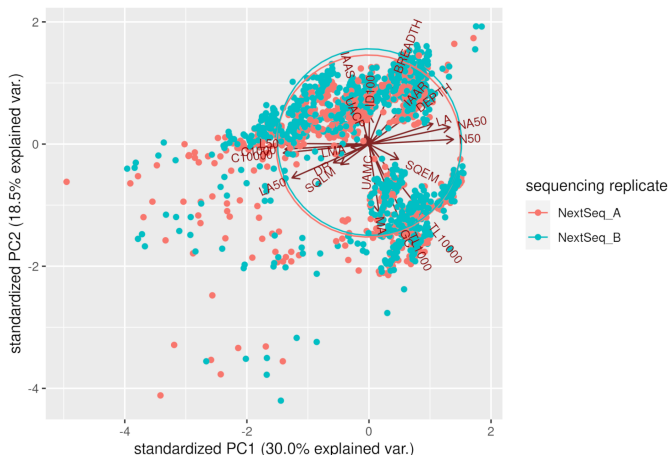**E**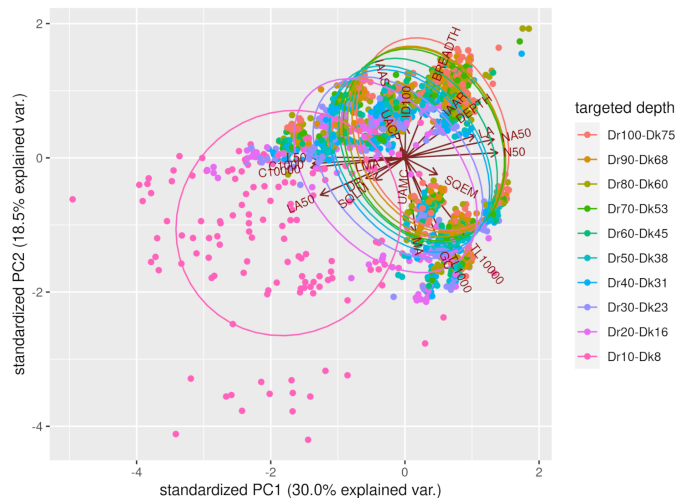**F**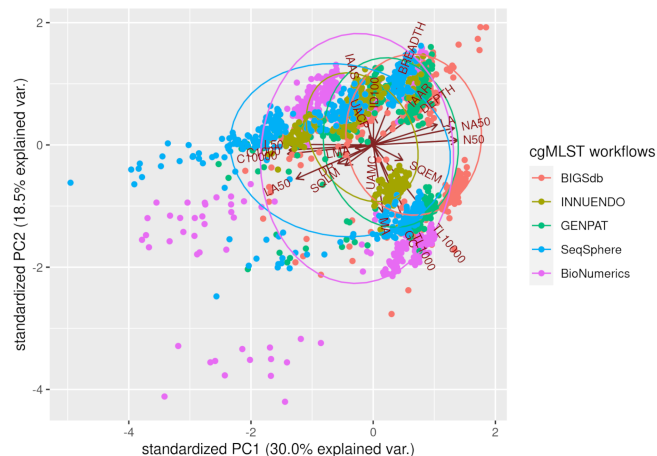

Supplement: Supplementary file 4 — Additional file 4 Principals component analyses (PCAs) of the numerical parameters C1000, C10000, DR, GC, IAAR, IAAS, ID100, L50, LA50, LA, LMA, MA, N50, NA50, DEPTH, BREADTH, SQEM, SQLM, TL1000, TL10000, UACP and UAMC (defined in the section abbreviations) according to the categorical parameters “reference genome” (A), “successive platings” (B), “DNA extraction replicate” (C), “sequencing replicate” (D), “targeted depth” (E), “cgMLST workflows” (F), including assembly-based cgMLST workflows BIGSdb (n = 420), INNUENDO (n = 336), GENPAT (n = 420), SeqSphere (n = 420) and BioNumerics (n = 420) applied to downsampled paired-end reads from 3 reference genomes of Listeria monocytogenes (i.e. ATCC19114, ATCC19115 and ATCCBAA679). The PCA parameters C0-C1000-C5000-C10000-C25000-C50000, GC-TL0-TL1000-TL5000-TL10000-TL25000-TL50000-TL-TAL-MACL, N50-NG50-N75-NG75-SQEM-NA50-NGA50-NA75-NGA75-LA, L50-LG50-L75-LG75, LA50-LGA50-LA75-LGA75, DEPTH-GF, LMA-UAL-MM100-SQLM, DR-N100-UAC and MA-MAC were overlapped and are consequently not presented together. [file 12864_2022_8437_MOESM4_ESM.pdf]

**A**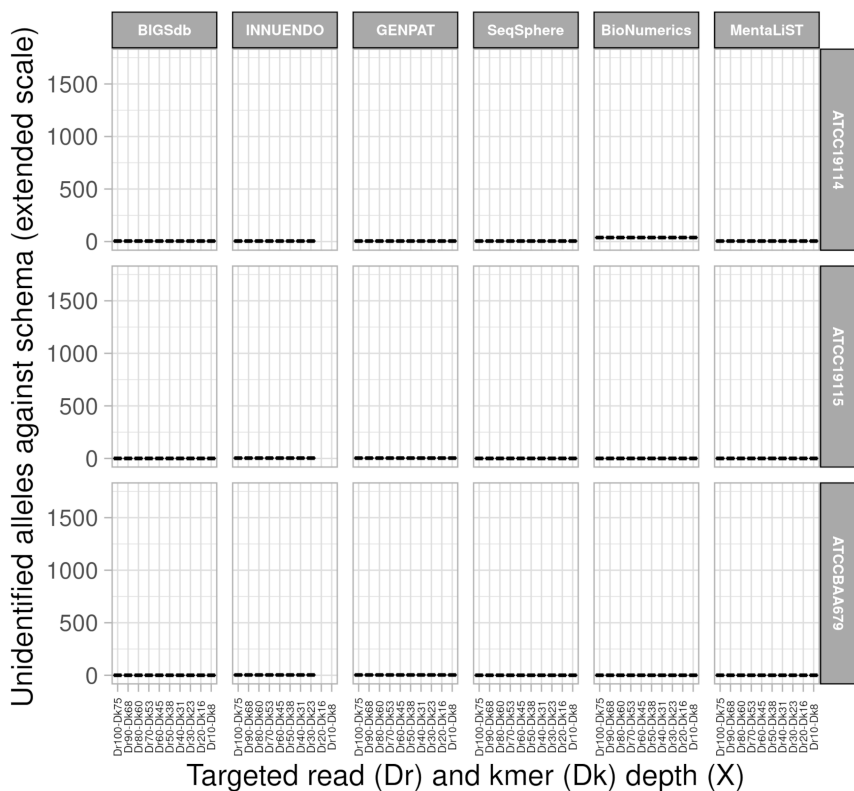**B**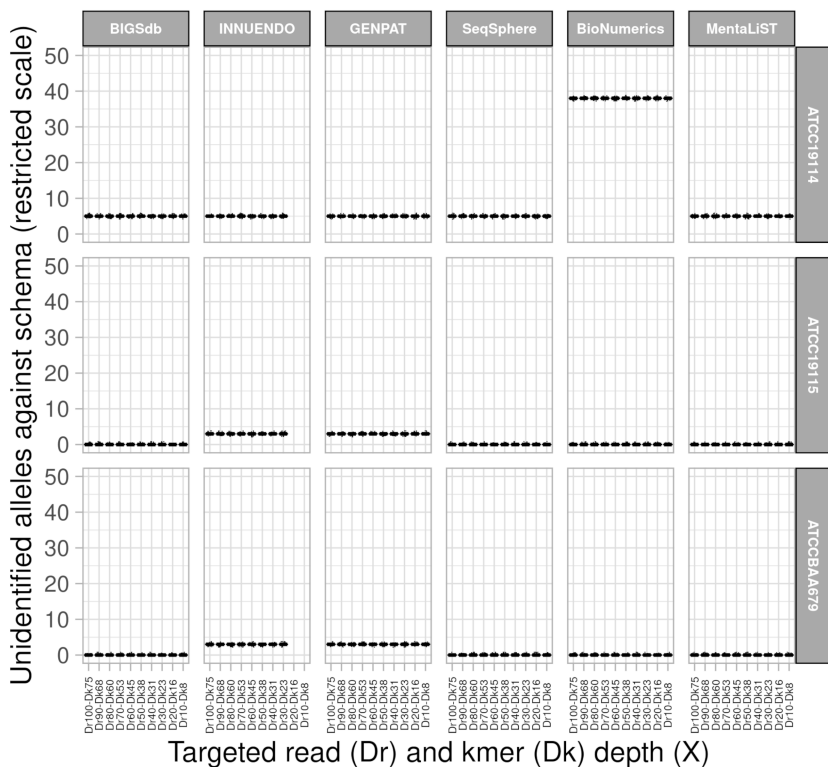

Supplement: Supplementary file 7 — Additional file 7 Box-plots representing the impact of downsampled paired-end reads (i.e. 2x150bp) of Listeria monocytogenes on unidentified alleles against schema at extended (A) or restricted (B) scales, according to reference genomes (i.e. ATCC19114, ATCC19115 and ATCCBAA679) and cgMLST workflows including BIGSdb (n = 420), INNUENDO (n = 336), GENPAT (n = 420), SeqSphere (n = 420), BioNumerics (n = 420) and MentaLiST (n = 420). The targeted read depth (Dr: 10X, 20X, 30X, 40X, 50X, 60X, 70X, 80X, 90X and 100X) were prepared according to kmer depth (Dk): 8X, 15X, 23X, 30X, 38X, 45X, 52X, 60X, 67X, 75X) setting of BBNorm (read length R = 150 and kmer size K = 30). Because of internal firewall, the INNUca assembler integrated into the cgMLST workflow INNUENDO cannot not perform assemblies of paired-end reads with read depth of coverage of 20X (n = 42) and 10X (n = 42). [file 12864_2022_8437_MOESM7_ESM.pdf]
