# Supplementary material for: In vitro and in silico parameters for precise cgMLST typing of Listeria monocytogenes
Source: BMC Genomics. 2022 Mar 26;23:235. doi: 10.1186/s12864-022-08437-4 (PMC8961897; doi:10.1186/s12864-022-08437-4)
Supplement: Supplementary file 5 — Additional file 5 Coefficients (Coef.) of the generalized linear models (GLMs with Poisson distribution and without overdispersion) comparing the parameters “identical alleles against reference circular genomes” (IAAR) with the parameters of interest “reference genome” (REFERENCE), “successive platings” (PLATING) (B), “DNA extraction replicate” (DNA), “sequencing replicate” (SEQUENCING), “read depth” (DEPTH), “read breadth” (BREADTH), assembly parameters (defined in the section abbreviations: C0, C1000, C10000, C25000, C5000, C50000, DR, GC, GF, ID100, L50, L75, LA, LA50, LA75, LC, LG50, LG75, LGA50, LGA75, LMA, MA, MAC, MACL, MM100, N100, N50, N75, NA50, NA75, NG50, NG75, NGA50, NGA75, SQEM, SQLM, TAL, TL, TL0, TL1000, TL10000, TL25000, TL5000, TL50000, UAC, UACP, UAL, UAMC), cgMLST workflows (WORKFLOW) together (A) and cgMLST workflows independently including BIGSdb (B: n = 420), INNUENDO (C: n = 336), GENPAT (D: n = 420), SeqSphere (E: n = 420) and BioNumerics (F: n = 420), applied to downsampled paired-end reads from 3 reference genomes of Listeria monocytogenes (i.e. ATCC19114, ATCC19115 and ATCCBAA679). Few parameters are not defined because of singularities. [file 12864_2022_8437_MOESM5_ESM.pdf]

A

Parameters	Coef. estimate	Coef. standard error	Coef. t value	Coef. P-value (>|t|)
N100	-3.3E-04	1.8E-05	-1.8E+01	2.0E-16
IAAS	5.8E-04	1.3E-04	4.6E+00	3.7E-06
UAL	-6.5E-06	2.4E-06	-2.7E+00	7.3E-03
TL0	6.1E-06	2.3E-06	2.7E+00	7.5E-03
TAL	-3.3E-06	1.8E-06	-1.8E+00	6.8E-02
C0	-2.8E-04	2.6E-04	-1.1E+00	2.8E-01
UAC	7.0E-04	6.6E-04	1.1E+00	2.9E-01
UACP	4.6E-03	4.4E-03	1.0E+00	3.0E-01
WORKFLOW: GENPAT	-2.7E-03	2.7E-03	-9.8E-01	3.3E-01
TL1000	-1.2E-06	1.3E-06	-9.5E-01	3.4E-01
C1000	1.1E-03	1.4E-03	7.9E-01	4.3E-01
REFERENCE: ATCCBAA679	3.8E-02	5.2E-02	7.3E-01	4.6E-01
REFERENCE: ATCC19115	3.1E-02	4.4E-02	7.2E-01	4.7E-01
MAC	6.8E-03	1.0E-02	6.5E-01	5.2E-01
WORKFLOW: INNUENDO	-2.0E-03	3.5E-03	-5.9E-01	5.6E-01
MA	-5.4E-03	9.2E-03	-5.9E-01	5.6E-01
GC	-7.7E-02	1.3E-01	-5.9E-01	5.6E-01
NG50	-5.4E-08	9.3E-08	-5.8E-01	5.6E-01
LG50	-6.9E-03	1.3E-02	-5.4E-01	5.9E-01
ID100	3.2E-03	6.2E-03	5.1E-01	6.1E-01
WORKFLOW: SeqSphere	-3.4E-03	6.9E-03	-4.9E-01	6.2E-01
N50	3.6E-08	7.8E-08	4.6E-01	6.4E-01
L50	5.6E-03	1.2E-02	4.5E-01	6.5E-01
N75	-8.4E-08	1.9E-07	-4.4E-01	6.6E-01
NG75	7.8E-08	1.9E-07	4.1E-01	6.8E-01
TL50000	-4.7E-08	1.3E-07	-3.6E-01	7.2E-01
LGA50	3.9E-03	1.3E-02	3.0E-01	7.6E-01
LA	1.6E-08	5.6E-08	2.9E-01	7.7E-01
BREADTH	4.3E-03	1.5E-02	2.9E-01	7.7E-01
L75	-2.9E-03	1.0E-02	-2.8E-01	7.8E-01
LG75	2.6E-03	9.5E-03	2.7E-01	7.9E-01
LC	-1.4E-08	5.6E-08	-2.6E-01	8.0E-01
GF	-8.6E-03	3.3E-02	-2.6E-01	8.0E-01
C50000	1.2E-03	4.9E-03	2.5E-01	8.0E-01
LA50	-3.2E-03	1.3E-02	-2.5E-01	8.0E-01
MM100	-2.4E-04	1.2E-03	-2.0E-01	8.4E-01
LGA75	-1.4E-03	7.6E-03	-1.8E-01	8.5E-01
NGA50	2.1E-08	1.1E-07	1.8E-01	8.6E-01
SEQUENCING: NextSeq_B	-2.1E-04	1.2E-03	-1.7E-01	8.6E-01
C5000	-9.3E-04	6.0E-03	-1.6E-01	8.8E-01
UAMC	-2.6E-03	1.7E-02	-1.5E-01	8.8E-01
LA75	1.3E-03	8.6E-03	1.5E-01	8.8E-01
MACL	-1.3E-09	9.5E-09	-1.4E-01	8.9E-01
NA75	1.5E-08	1.1E-07	1.4E-01	8.9E-01
DEPTH	-4.4E-06	3.5E-05	-1.2E-01	9.0E-01

NGA75	-1.1E-08	1.0E-07	-1.1E-01	9.1E-01
DR	-3.0E-01	3.1E+00	-9.9E-02	9.2E-01
C10000	-7.6E-04	7.8E-03	-9.8E-02	9.2E-01
DNA: extraction_A	1.4E-04	1.5E-03	9.8E-02	9.2E-01
TL5000	1.1E-07	1.2E-06	9.6E-02	9.2E-01
C25000	-6.1E-04	8.0E-03	-7.6E-02	9.4E-01
NA50	-6.9E-09	1.0E-07	-6.7E-02	9.5E-01
PLATING: tenth_culture	9.5E-05	2.0E-03	4.8E-02	9.6E-01
TL10000	-3.4E-08	9.8E-07	-3.5E-02	9.7E-01
PLATING: fifth_culture	6.2E-05	1.9E-03	3.2E-02	9.7E-01
LMA	-2.2E-05	7.3E-04	-3.0E-02	9.8E-01
SQLM	-1.8E-05	7.7E-04	-2.4E-02	9.8E-01
TL25000	6.2E-09	3.7E-07	1.7E-02	9.9E-01
SQEM	-6.4E-05	3.8E-03	-1.7E-02	9.9E-01
WORKFLOW: BioNumerics	-5.5E-05	3.7E-03	-1.5E-02	9.9E-01
DNA: extraction_B	1.2E-05	1.4E-03	8.2E-03	9.9E-01
**Model intercept**	**5.3E+00**	**6.0E+00**	**8.9E-01**	**3.7E-01**

B

Parameters	Coef. estimate	Coef. standard error	Coef. t value	Coef. P-value (>|t|)
C25000	4.0E-03	3.7E-02	1.1E-01	9.1E-01
TL25000	-2.1E-07	2.0E-06	-1.1E-01	9.1E-01
C10000	-3.3E-03	3.6E-02	-9.1E-02	9.3E-01
REFERENCE: ATCC19115	1.2E-02	1.4E-01	9.0E-02	9.3E-01
LMA	-1.4E-04	1.7E-03	-8.7E-02	9.3E-01
LGA75	-1.6E-03	1.9E-02	-8.3E-02	9.3E-01
REFERENCE: ATCCBAA679	1.3E-02	1.6E-01	7.8E-02	9.4E-01
MA	-1.2E-03	1.6E-02	-7.7E-02	9.4E-01
DR	-7.3E-01	1.2E+01	-6.0E-02	9.5E-01
GF	-7.3E-03	1.2E-01	-5.9E-02	9.5E-01
ID100	-7.3E-04	1.6E-02	-4.7E-02	9.6E-01
LA75	1.5E-03	3.2E-02	4.5E-02	9.6E-01
TL10000	1.5E-07	3.4E-06	4.5E-02	9.6E-01
LG75	1.1E-03	2.4E-02	4.5E-02	9.6E-01
C1000	-2.2E-04	5.8E-03	-3.9E-02	9.7E-01
MACL	8.3E-10	2.2E-08	3.8E-02	9.7E-01
C50000	-5.3E-04	1.6E-02	-3.4E-02	9.7E-01
PLATING: tenth_culture	1.0E-04	4.5E-03	2.2E-02	9.8E-01
GC	7.8E-03	3.6E-01	2.2E-02	9.8E-01
L75	-7.3E-04	3.4E-02	-2.2E-02	9.8E-01
TL50000	8.8E-09	4.1E-07	2.2E-02	9.8E-01
C0	8.4E-05	4.1E-03	2.0E-02	9.8E-01
MM100	5.3E-05	2.6E-03	2.0E-02	9.8E-01
NA50	7.7E-09	4.0E-07	1.9E-02	9.8E-01
UAL	-2.3E-07	1.3E-05	-1.8E-02	9.9E-01
NA75	8.3E-09	4.7E-07	1.8E-02	9.9E-01
N100	-1.0E-05	6.0E-04	-1.7E-02	9.9E-01
C5000	-2.9E-04	1.7E-02	-1.7E-02	9.9E-01

NGA75	-6.8E-09	4.2E-07	-1.6E-02	9.9E-01
L50	-8.7E-04	5.5E-02	-1.6E-02	9.9E-01
DNA: extraction_A	-5.2E-05	3.4E-03	-1.5E-02	9.9E-01
N50	-9.6E-09	6.3E-07	-1.5E-02	9.9E-01
DEPTH	1.2E-06	8.1E-05	1.5E-02	9.9E-01
NGA50	-5.2E-09	3.7E-07	-1.4E-02	9.9E-01
TL0	1.5E-07	1.1E-05	1.4E-02	9.9E-01
LA50	4.8E-04	3.7E-02	1.3E-02	9.9E-01
MAC	3.0E-04	2.3E-02	1.3E-02	9.9E-01
LG50	6.7E-04	5.1E-02	1.3E-02	9.9E-01
LC	1.5E-09	1.3E-07	1.2E-02	9.9E-01
SQEM	-7.2E-05	6.2E-03	-1.2E-02	9.9E-01
TL1000	9.2E-08	8.0E-06	1.1E-02	9.9E-01
TAL	3.9E-08	3.8E-06	1.0E-02	9.9E-01
UAC	1.2E-04	1.2E-02	9.7E-03	9.9E-01
NG50	5.9E-09	6.1E-07	9.5E-03	9.9E-01
UACP	1.6E-04	2.0E-02	7.7E-03	9.9E-01
SEQUENCING: NextSeq_B	-2.1E-05	2.8E-03	-7.6E-03	9.9E-01
SQLM	1.4E-05	2.3E-03	6.2E-03	1.0E+00
LGA50	-1.9E-04	3.1E-02	-6.1E-03	1.0E+00
BREADTH	2.1E-04	3.5E-02	5.9E-03	1.0E+00
DNA; extraction_B	-1.2E-05	3.3E-03	-3.7E-03	1.0E+00
NG75	1.4E-09	3.8E-07	3.6E-03	1.0E+00
LA	-3.6E-10	1.2E-07	-2.9E-03	1.0E+00
TL5000	8.3E-09	3.4E-06	2.4E-03	1.0E+00
N75	-5.7E-10	3.6E-07	-1.6E-03	1.0E+00
PLATING: fifth_culture	6.4E-06	4.4E-03	1.4E-03	1.0E+00
**Model intercept**	**7.9E+00**	**1.8E+01**	**4.5E-01**	**6.5E-01**

C

Parameters	Coef. estimate	Coef. standard error	Coef. t value	Coef. P-value (>|t|)
MM100	-1.2E-04	5.6E-03	-2.1E-02	9.8E-01
ID100	3.5E-04	1.8E-02	1.9E-02	9.8E-01
PLATING: tenth_culture	9.1E-05	5.2E-03	1.7E-02	9.9E-01
PLATING: fifth_culture	5.5E-05	5.1E-03	1.1E-02	9.9E-01
C0	-2.8E-05	2.7E-03	-1.0E-02	9.9E-01
TL1000	-5.9E-08	5.9E-06	-9.9E-03	9.9E-01
DNA: extraction_B	-3.6E-05	3.9E-03	-9.4E-03	9.9E-01
C1000	3.0E-05	4.6E-03	6.7E-03	9.9E-01
LMA	1.9E-05	3.4E-03	5.7E-03	1.0E+00
C50000	-1.2E-04	2.1E-02	-5.6E-03	1.0E+00
GC	2.0E-03	3.7E-01	5.5E-03	1.0E+00
DEPTH	-5.1E-07	9.5E-05	-5.4E-03	1.0E+00
TL50000	2.5E-09	5.9E-07	4.2E-03	1.0E+00
REFERENCE: ATCC19115	1.7E-03	4.1E-01	4.1E-03	1.0E+00
SEQUENCING: NextSeq_B	1.2E-05	3.3E-03	3.7E-03	1.0E+00
C25000	1.3E-04	3.7E-02	3.7E-03	1.0E+00
REFERENCE: ATCCBAA679	1.7E-03	5.0E-01	3.4E-03	1.0E+00

L50	-4.8E-01	1.4E+02	-3.3E-03	1.0E+00
LA50	4.8E-01	1.4E+02	3.3E-03	1.0E+00
L75	2.2E-01	6.6E+01	3.3E-03	1.0E+00
LA75	-2.2E-01	6.6E+01	-3.3E-03	1.0E+00
MACL	-4.7E-07	1.4E-04	-3.3E-03	1.0E+00
MA	1.9E-04	7.0E-02	2.7E-03	1.0E+00
UAC	5.8E-05	2.3E-02	2.5E-03	1.0E+00
C5000	3.8E-05	1.6E-02	2.3E-03	1.0E+00
TAL	9.9E-08	4.7E-05	2.1E-03	1.0E+00
LG75	-6.6E-05	3.2E-02	-2.0E-03	1.0E+00
LA	1.9E-07	9.2E-05	2.0E-03	1.0E+00
LC	-1.9E-07	9.2E-05	-2.0E-03	1.0E+00
DNA: extraction_A	6.8E-06	4.0E-03	1.7E-03	1.0E+00
GF	7.2E-04	4.3E-01	1.7E-03	1.0E+00
TL10000	5.3E-09	3.3E-06	1.6E-03	1.0E+00
C10000	-4.9E-05	3.5E-02	-1.4E-03	1.0E+00
BREADTH	-4.0E-05	3.6E-02	-1.1E-03	1.0E+00
TL0	-4.3E-08	4.5E-05	-9.5E-04	1.0E+00
TL5000	2.3E-09	3.4E-06	6.7E-04	1.0E+00
LG50	-2.9E-05	4.9E-02	-5.9E-04	1.0E+00
TL25000	-1.2E-09	2.1E-06	-5.4E-04	1.0E+00
UAL	-1.2E-08	5.0E-05	-2.3E-04	1.0E+00
N75	1.1E-06	6.6E-03	1.7E-04	1.0E+00
NA75	-1.1E-06	6.6E-03	-1.7E-04	1.0E+00
NA50	1.8E-07	1.6E-03	1.1E-04	1.0E+00
NGA75	-7.2E-07	6.6E-03	-1.1E-04	1.0E+00
NG75	7.2E-07	6.6E-03	1.1E-04	1.0E+00
N50	-1.8E-07	1.6E-03	-1.1E-04	1.0E+00
NGA50	-1.7E-07	1.6E-03	-1.0E-04	1.0E+00
NG50	1.7E-07	1.6E-03	1.0E-04	1.0E+00
**Model intercept**	**7.3E+00**	**1.4E+01**	**5.2E-01**	**6.0E-01**

D

Parameters	Coef. estimate	Coef. standard error	Coef. t value	Coef. P-value (>|t|)
LGA50	3.0E-03	4.1E-02	7.2E-02	9.4E-01
NGA50	3.8E-08	7.7E-07	4.9E-02	9.6E-01
REFERENCE: ATCC19115	9.8E-03	2.2E-01	4.5E-02	9.6E-01
REFERENCE: ATCCBAA679	1.2E-02	2.7E-01	4.4E-02	9.7E-01
NA50	-3.4E-08	8.0E-07	-4.3E-02	9.7E-01
C1000	-2.1E-04	5.0E-03	-4.2E-02	9.7E-01
LA	1.9E-08	5.0E-07	3.7E-02	9.7E-01
LC	-1.8E-08	5.0E-07	-3.7E-02	9.7E-01
MAC	-1.0E-03	3.3E-02	-3.1E-02	9.8E-01
TL50000	-1.6E-08	5.4E-07	-3.0E-02	9.8E-01
ID100	-4.8E-04	1.6E-02	-2.9E-02	9.8E-01
PLATING: tenth_culture	1.3E-04	4.8E-03	2.8E-02	9.8E-01
TL0	3.1E-07	1.1E-05	2.7E-02	9.8E-01
DNA: extraction_B	-9.0E-05	3.4E-03	-2.7E-02	9.8E-01

LG50	-2.6E-03	9.6E-02	-2.7E-02	9.8E-01
NG50	-4.1E-08	1.6E-06	-2.6E-02	9.8E-01
GF	-5.5E-03	2.2E-01	-2.5E-02	9.8E-01
MM100	-7.3E-05	3.1E-03	-2.4E-02	9.8E-01
N50	3.5E-08	1.6E-06	2.2E-02	9.8E-01
TL1000	9.0E-08	4.6E-06	2.0E-02	9.8E-01
UAC	-8.5E-05	4.9E-03	-1.7E-02	9.9E-01
MA	5.1E-04	3.0E-02	1.7E-02	9.9E-01
C10000	-4.9E-04	3.1E-02	-1.6E-02	9.9E-01
NG75	-1.1E-07	6.8E-06	-1.6E-02	9.9E-01
LA75	1.6E-03	1.0E-01	1.6E-02	9.9E-01
N75	9.4E-08	6.8E-06	1.4E-02	9.9E-01
UAL	-1.8E-07	1.3E-05	-1.3E-02	9.9E-01
TL10000	-4.8E-08	4.0E-06	-1.2E-02	9.9E-01
BREADTH	3.9E-04	3.5E-02	1.1E-02	9.9E-01
TL25000	-1.6E-08	1.5E-06	-1.1E-02	9.9E-01
N100	-9.3E-06	8.8E-04	-1.0E-02	9.9E-01
DR	2.9E-01	2.8E+01	1.0E-02	9.9E-01
NGA75	6.9E-08	6.7E-06	1.0E-02	9.9E-01
SQLM	-3.4E-05	3.4E-03	-9.9E-03	9.9E-01
C50000	2.0E-04	2.1E-02	9.8E-03	9.9E-01
PLATING: fifth_culture	4.4E-05	4.6E-03	9.6E-03	9.9E-01
LA50	-5.2E-04	5.9E-02	-8.8E-03	9.9E-01
NA75	-5.8E-08	6.7E-06	-8.7E-03	9.9E-01
LMA	-1.7E-05	2.1E-03	-8.3E-03	9.9E-01
DEPTH	-6.3E-07	8.2E-05	-7.7E-03	9.9E-01
LGA75	-5.5E-04	7.2E-02	-7.6E-03	9.9E-01
C0	1.3E-05	1.8E-03	7.3E-03	9.9E-01
SEQUENCING: NextSeq_B	-2.2E-05	3.1E-03	-7.1E-03	9.9E-01
L75	-6.9E-04	1.1E-01	-6.6E-03	9.9E-01
DNA: extraction_A	-1.6E-05	3.5E-03	-4.5E-03	1.0E+00
LG75	-3.3E-04	8.0E-02	-4.1E-03	1.0E+00
C25000	-1.1E-04	3.1E-02	-3.7E-03	1.0E+00
TL5000	1.7E-08	4.8E-06	3.4E-03	1.0E+00
TAL	-2.3E-08	7.3E-06	-3.2E-03	1.0E+00
L50	3.1E-04	1.2E-01	2.7E-03	1.0E+00
C5000	-6.4E-05	2.5E-02	-2.5E-03	1.0E+00
SQEM	3.1E-05	1.3E-02	2.3E-03	1.0E+00
GC	-4.2E-04	3.5E-01	-1.2E-03	1.0E+00
UACP	-1.0E-05	1.9E-02	-5.5E-04	1.0E+00
MACL	-4.6E-12	4.2E-08	-1.1E-04	1.0E+00
**Model intercept**	**6.8E+00**	**3.0E+01**	**2.3E-01**	**8.2E-01**

E

Parameters	Coef. estimate	Coef. standard error	Coef. t value	Coef. P-value (>|t|)
LA75	-1.4E-03	1.6E-02	-8.5E-02	9.3E-01
L75	1.7E-03	2.1E-02	7.7E-02	9.4E-01
LG75	-1.4E-03	2.2E-02	-6.3E-02	9.5E-01
NA75	-1.1E-08	1.9E-07	-6.1E-02	9.5E-01
C5000	7.5E-04	1.2E-02	6.1E-02	9.5E-01
TL5000	-1.4E-07	2.3E-06	-6.0E-02	9.5E-01
REFERENCE: ATCC19115	1.2E-02	2.1E-01	5.8E-02	9.5E-01
NGA75	1.0E-08	1.8E-07	5.8E-02	9.5E-01
LGA75	9.1E-04	1.6E-02	5.7E-02	9.5E-01
NG75	-2.6E-08	5.3E-07	-5.0E-02	9.6E-01
N75	2.6E-08	5.3E-07	4.9E-02	9.6E-01
REFERENCE: ATCCBAA679	1.3E-02	2.6E-01	4.9E-02	9.6E-01
C1000	-1.7E-04	3.5E-03	-4.7E-02	9.6E-01
TL10000	8.7E-08	1.9E-06	4.6E-02	9.6E-01
MA	-4.4E-04	1.0E-02	-4.2E-02	9.7E-01
LMA	-8.1E-05	2.0E-03	-4.1E-02	9.7E-01
C10000	-5.9E-04	1.5E-02	-4.1E-02	9.7E-01
NG50	1.8E-08	5.0E-07	3.6E-02	9.7E-01
NGA50	-1.7E-08	5.1E-07	-3.3E-02	9.7E-01
LG50	1.8E-03	5.9E-02	3.1E-02	9.8E-01
UAC	-3.2E-05	1.1E-03	-2.8E-02	9.8E-01
SEQUENCING: NextSeq_B	-8.2E-05	2.9E-03	-2.8E-02	9.8E-01
L50	-1.5E-03	5.5E-02	-2.7E-02	9.8E-01
PLATING: fifth_culture	1.2E-04	4.4E-03	2.7E-02	9.8E-01
PLATING: tenth_culture	1.1E-04	4.6E-03	2.4E-02	9.8E-01
MACL	1.0E-09	4.5E-08	2.3E-02	9.8E-01
BREADTH	7.9E-04	3.5E-02	2.3E-02	9.8E-01
LA50	1.3E-03	5.6E-02	2.3E-02	9.8E-01
LA	6.0E-09	2.7E-07	2.2E-02	9.8E-01
LGA50	-1.3E-03	6.1E-02	-2.2E-02	9.8E-01
N50	-8.6E-09	4.2E-07	-2.1E-02	9.8E-01
ID100	-3.3E-04	1.6E-02	-2.0E-02	9.8E-01
LC	-5.1E-09	2.7E-07	-1.9E-02	9.8E-01
TL0	1.8E-07	1.1E-05	1.7E-02	9.9E-01
NA50	7.1E-09	4.4E-07	1.6E-02	9.9E-01
UACP	-2.4E-04	1.6E-02	-1.5E-02	9.9E-01
DR	-5.2E-02	4.2E+00	-1.2E-02	9.9E-01
TL25000	8.2E-09	7.0E-07	1.2E-02	9.9E-01
TAL	1.1E-07	9.6E-06	1.1E-02	9.9E-01
UAL	-1.1E-07	1.0E-05	-1.1E-02	9.9E-01
TL1000	3.4E-08	3.4E-06	1.0E-02	9.9E-01
N100	-1.6E-03	1.8E-01	-9.1E-03	9.9E-01
DNA: extraction_A	-2.9E-05	3.5E-03	-8.4E-03	9.9E-01
DEPTH	5.6E-07	8.6E-05	6.5E-03	9.9E-01
TL50000	-1.8E-09	2.8E-07	-6.4E-03	9.9E-01

GF	-1.3E-03	2.2E-01	-6.0E-03	1.0E+00
C50000	-6.8E-05	1.1E-02	-6.0E-03	1.0E+00
MM100	1.7E-05	3.4E-03	5.1E-03	1.0E+00
C25000	-7.7E-05	1.7E-02	-4.6E-03	1.0E+00
GC	1.4E-03	3.2E-01	4.3E-03	1.0E+00
DNA: extraction_B	-6.5E-06	3.3E-03	-2.0E-03	1.0E+00
C0	1.1E-06	7.0E-04	1.6E-03	1.0E+00
**Model intercept**	**6.7E+00**	**1.6E+01**	**4.1E-01**	**6.8E-01**

F

Parameters	Coef. estimate	Coef. standard error	Coef. t value	Coef. P-value (>|t|)
N100	-3.4E-04	3.4E-05	-1.0E+01	2.0E-16
LC	-2.6E-07	2.7E-07	-9.8E-01	3.3E-01
LA	2.7E-07	2.7E-07	9.8E-01	3.3E-01
NG50	-2.1E-07	2.3E-07	-9.3E-01	3.5E-01
MACL	3.1E-08	3.8E-08	8.1E-01	4.2E-01
L50	2.3E-02	3.0E-02	7.8E-01	4.4E-01
N50	1.3E-07	1.7E-07	7.5E-01	4.5E-01
LG50	-2.1E-02	3.0E-02	-7.1E-01	4.8E-01
SQEM	1.5E-02	3.5E-02	4.4E-01	6.6E-01
TL0	4.6E-06	1.1E-05	4.4E-01	6.6E-01
C1000	2.6E-03	6.3E-03	4.0E-01	6.9E-01
TL25000	-4.3E-07	1.2E-06	-3.7E-01	7.1E-01
REFERENCE: ATCC19115	7.3E-02	2.0E-01	3.6E-01	7.2E-01
BREADTH	1.2E-02	3.5E-02	3.5E-01	7.3E-01
SQLM	5.5E-04	1.6E-03	3.4E-01	7.3E-01
REFERENCE: ATCCBAA679	8.1E-02	2.4E-01	3.3E-01	7.4E-01
C10000	-9.0E-03	2.8E-02	-3.3E-01	7.4E-01
LA50	-1.2E-02	3.9E-02	-3.1E-01	7.6E-01
SEQUENCING: NextSeq_B	-8.4E-04	2.9E-03	-2.9E-01	7.8E-01
LA75	4.7E-03	1.8E-02	2.7E-01	7.9E-01
L75	-8.7E-03	3.4E-02	-2.6E-01	8.0E-01
LGA75	-4.1E-03	1.7E-02	-2.4E-01	8.1E-01
UAL	-2.8E-06	1.2E-05	-2.3E-01	8.2E-01
C25000	5.9E-03	2.6E-02	2.3E-01	8.2E-01
TAL	-1.7E-06	7.9E-06	-2.2E-01	8.3E-01
NGA50	8.3E-08	3.9E-07	2.1E-01	8.3E-01
TL10000	7.0E-07	3.4E-06	2.0E-01	8.4E-01
UAC	8.7E-04	4.4E-03	2.0E-01	8.4E-01
NGA75	-4.4E-08	2.4E-07	-1.8E-01	8.5E-01
LGA50	6.8E-03	4.0E-02	1.7E-01	8.6E-01
C50000	3.4E-03	2.1E-02	1.6E-01	8.7E-01
LG75	5.4E-03	3.4E-02	1.6E-01	8.7E-01
UAMC	-2.9E-03	1.9E-02	-1.6E-01	8.8E-01
DR	1.3E+00	9.0E+00	1.5E-01	8.8E-01
ID100	2.4E-03	1.7E-02	1.5E-01	8.8E-01
TL1000	-1.3E-06	8.7E-06	-1.4E-01	8.8E-01
LMA	-2.3E-04	1.7E-03	-1.4E-01	8.9E-01

MA	-4.2E-03	3.7E-02	-1.1E-01	9.1E-01
PLATING: tenth_culture	5.2E-04	4.7E-03	1.1E-01	9.1E-01
TL50000	-6.5E-08	5.9E-07	-1.1E-01	9.1E-01
DEPTH	-1.0E-05	9.3E-05	-1.1E-01	9.1E-01
GF	-2.2E-02	2.2E-01	-9.9E-02	9.2E-01
C0	-3.9E-04	4.1E-03	-9.4E-02	9.3E-01
PLATING: fifth_culture	4.0E-04	4.5E-03	9.0E-02	9.3E-01
NG75	1.0E-07	1.3E-06	8.0E-02	9.4E-01
MAC	2.5E-03	3.9E-02	6.5E-02	9.5E-01
N75	-6.4E-08	1.2E-06	-5.2E-02	9.6E-01
NA75	1.3E-08	2.7E-07	4.7E-02	9.6E-01
GC	1.5E-02	3.7E-01	4.0E-02	9.7E-01
MM100	-9.0E-05	2.7E-03	-3.3E-02	9.7E-01
NA50	1.1E-08	3.4E-07	3.3E-02	9.7E-01
DNA: extraction_B	5.5E-05	3.5E-03	1.6E-02	9.9E-01
DNA: extraction_A	3.7E-05	3.4E-03	1.1E-02	9.9E-01
C5000	-2.0E-04	2.1E-02	-9.5E-03	9.9E-01
UACP	-1.7E-04	2.1E-02	-8.1E-03	9.9E-01
TL5000	1.6E-08	3.5E-06	4.6E-03	1.0E+00
**Model intercept**	**9.9E-01**	**1.8E+01**	**5.6E-02**	**9.6E-01**
