# Supplementary material for: In vitro and in silico parameters for precise cgMLST typing of Listeria monocytogenes
Source: BMC Genomics. 2022 Mar 26;23:235. doi: 10.1186/s12864-022-08437-4 (PMC8961897; doi:10.1186/s12864-022-08437-4)
Supplement: Supplementary file 6 — Additional file 6 Box-plots representing the impact of downsampled paired-end reads (i.e. 2x150bp) of reference genomes of Listeria monocytogenes (i.e. ATCC19114, ATCC19115 and ATCCBAA679), on cgMLST outcomes (BIGSdb: n = 420, INNUENDO: n = 336, GENPAT: n = 420, SeqSphere: n = 420, BioNumerics: n = 420 and MentaLiST: n = 420), including identified alleles against schema (A, B, C, D) or identical alleles against reference circular genomes at extended (E, F, G, H) or restricted (I, J, K, L) scales, according to reference genomes (A, E, I), successive platings (B, F, J), DNA extraction replicate (C, G, K) and sequencing replicate (C, H, L). The targeted read depth (Dr: 10X, 20X, 30X, 40X, 50X, 60X, 70X, 80X, 90X and 100X) were prepared according to kmer depth (Dk): 8X, 15X, 23X, 30X, 38X, 45X, 52X, 60X, 67X, 75X) setting of BBNorm (read length R = 150 and kmer size K = 30). Because of internal firewall, the INNUca assembler integrated into the cgMLST workflow INNUENDO cannot not perform assemblies of paired-end reads with read depth of coverage of 20X (n = 42) and 10X (n = 42). [file 12864_2022_8437_MOESM6_ESM.pdf]

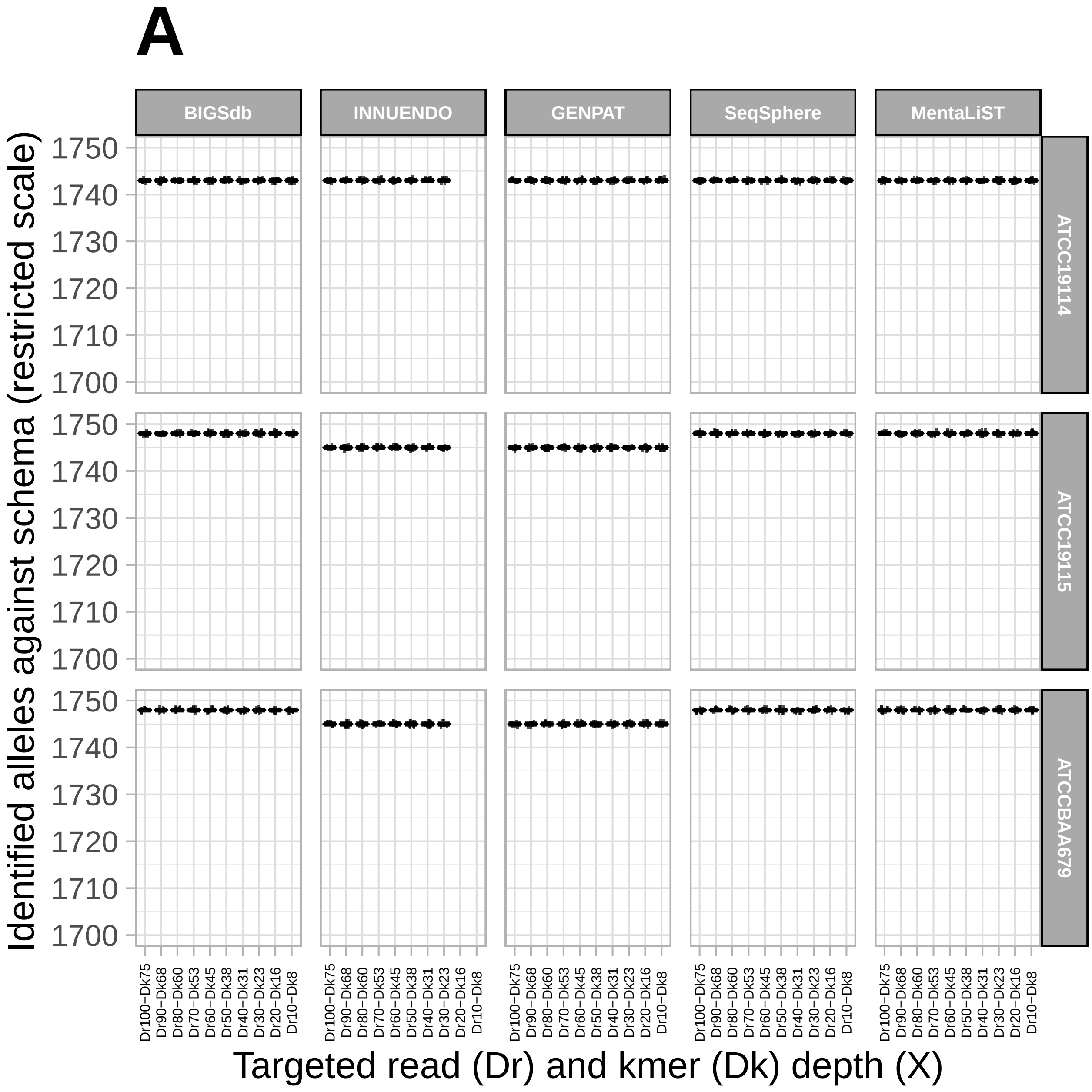
A
BIGSdb
INNUENDO
GENPAT
SeqSphere
MentaLiST
ATCC19114
ATCC19115
ATCCBAA679
Identified alleles against schema (restricted scale)
1750
1740
1730
1720
1710
1700
Dr100−Dk75
Dr90−Dk68
Dr80−Dk60
Dr70−Dk53
Dr60−Dk45
Dr50−Dk38
Dr40−Dk31
Dr30−Dk23
Dr20−Dk16
Dr10−Dk8
Targeted read (Dr) and kmer (Dk) depth (X)

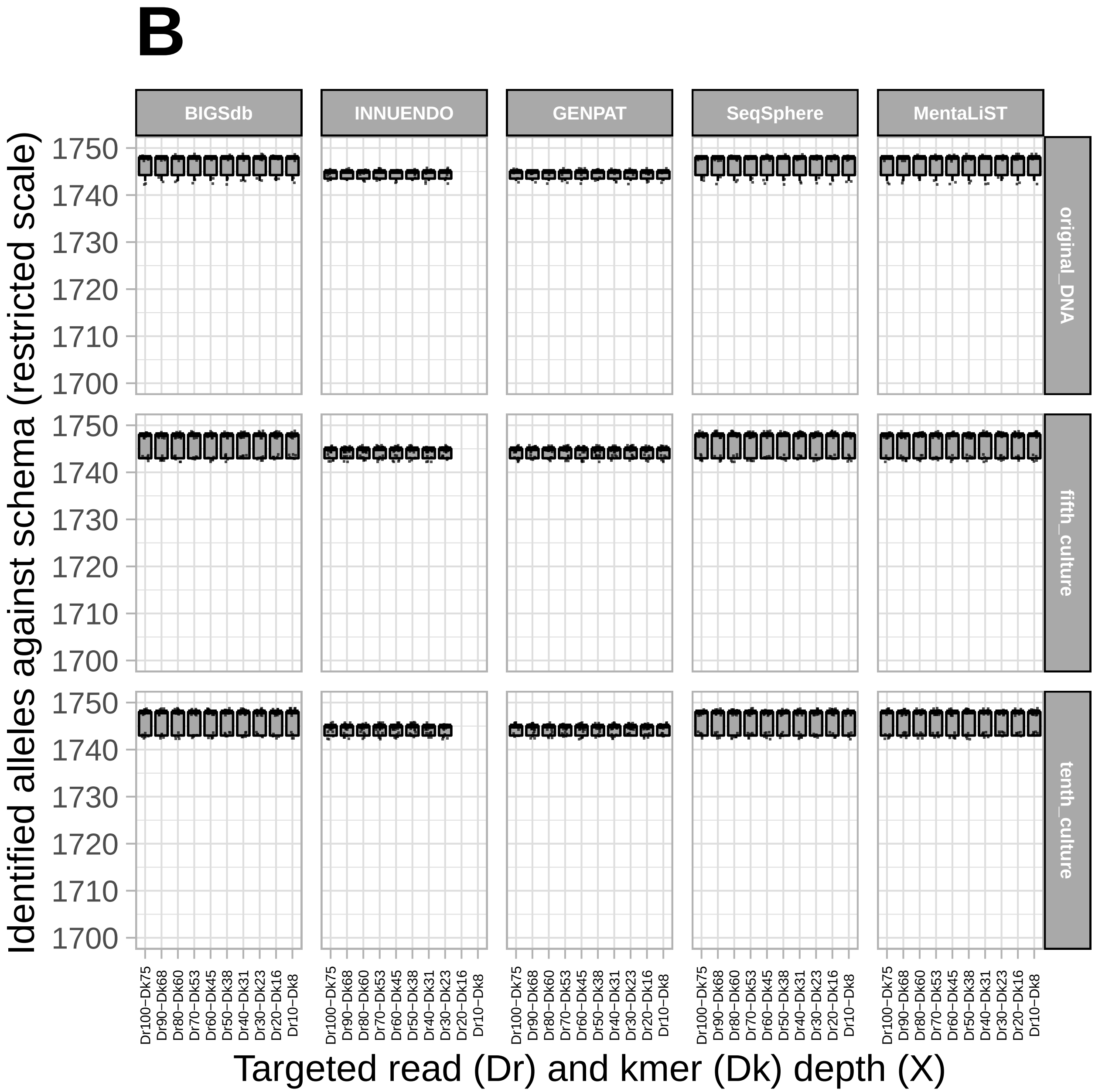
B
BIGSdb
INNUENDO
GENPAT
SeqSphere
MentaLiST
original_DNA
fifth_culture
tenth_culture
Identified alleles against schema (restricted scale)
1750
1740
1730
1720
1710
1700
Dr100−Dk75
Dr90−Dk68
Dr80−Dk60
Dr70−Dk53
Dr60−Dk45
Dr50−Dk38
Dr40−Dk31
Dr30−Dk23
Dr20−Dk16
Dr10−Dk8
Targeted read (Dr) and kmer (Dk) depth (X)

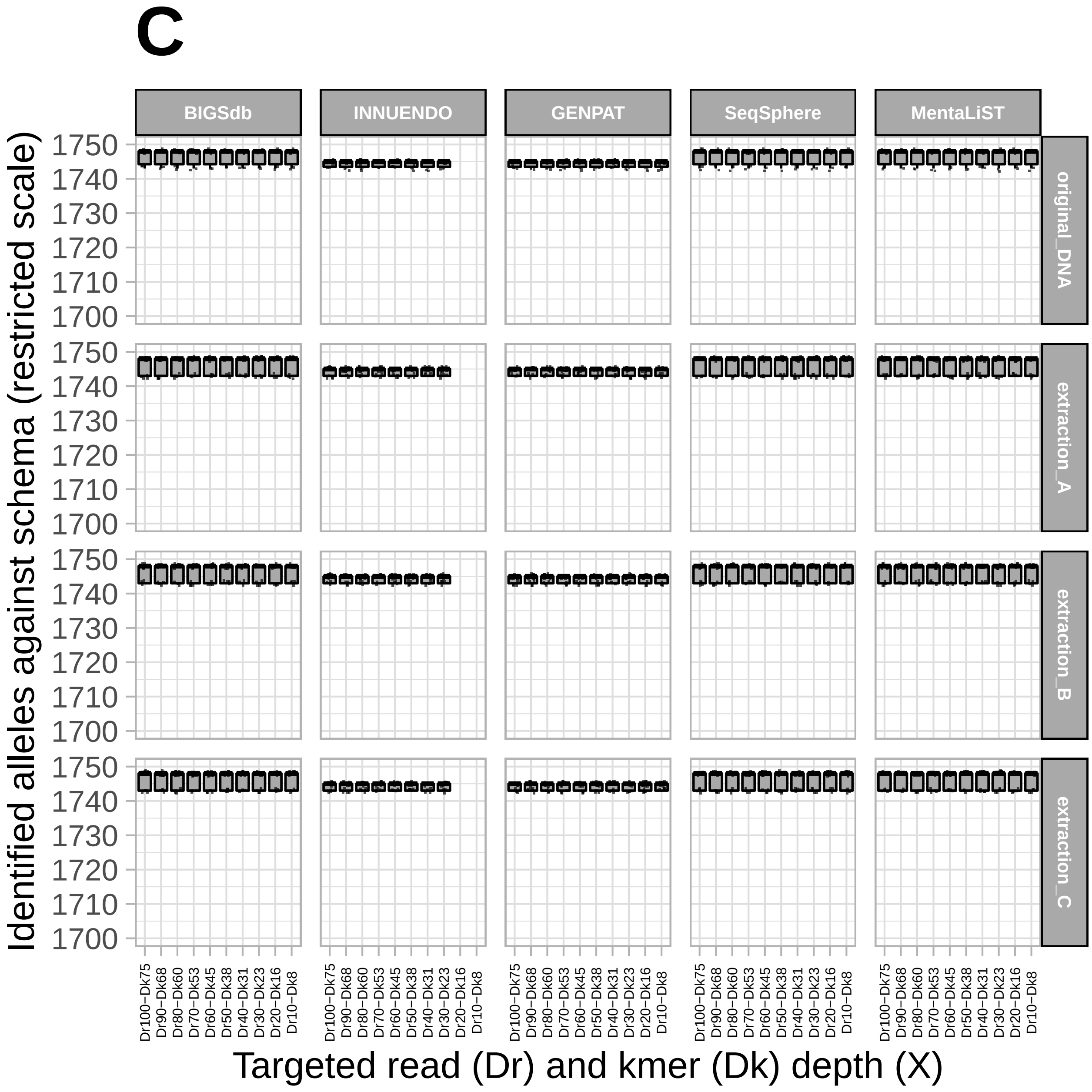
C
BIGSdb
INNUENDO
GENPAT
SeqSphere
MentaLiST
original_DNA
extraction_A
extraction_B
extraction_C
Identified alleles against schema (restricted scale)
1750
1740
1730
1720
1710
1700
Dr100−Dk75
Dr90−Dk68
Dr80−Dk60
Dr70−Dk53
Dr60−Dk45
Dr50−Dk38
Dr40−Dk31
Dr30−Dk23
Dr20−Dk16
Dr10−Dk8
Targeted read (Dr) and kmer (Dk) depth (X)

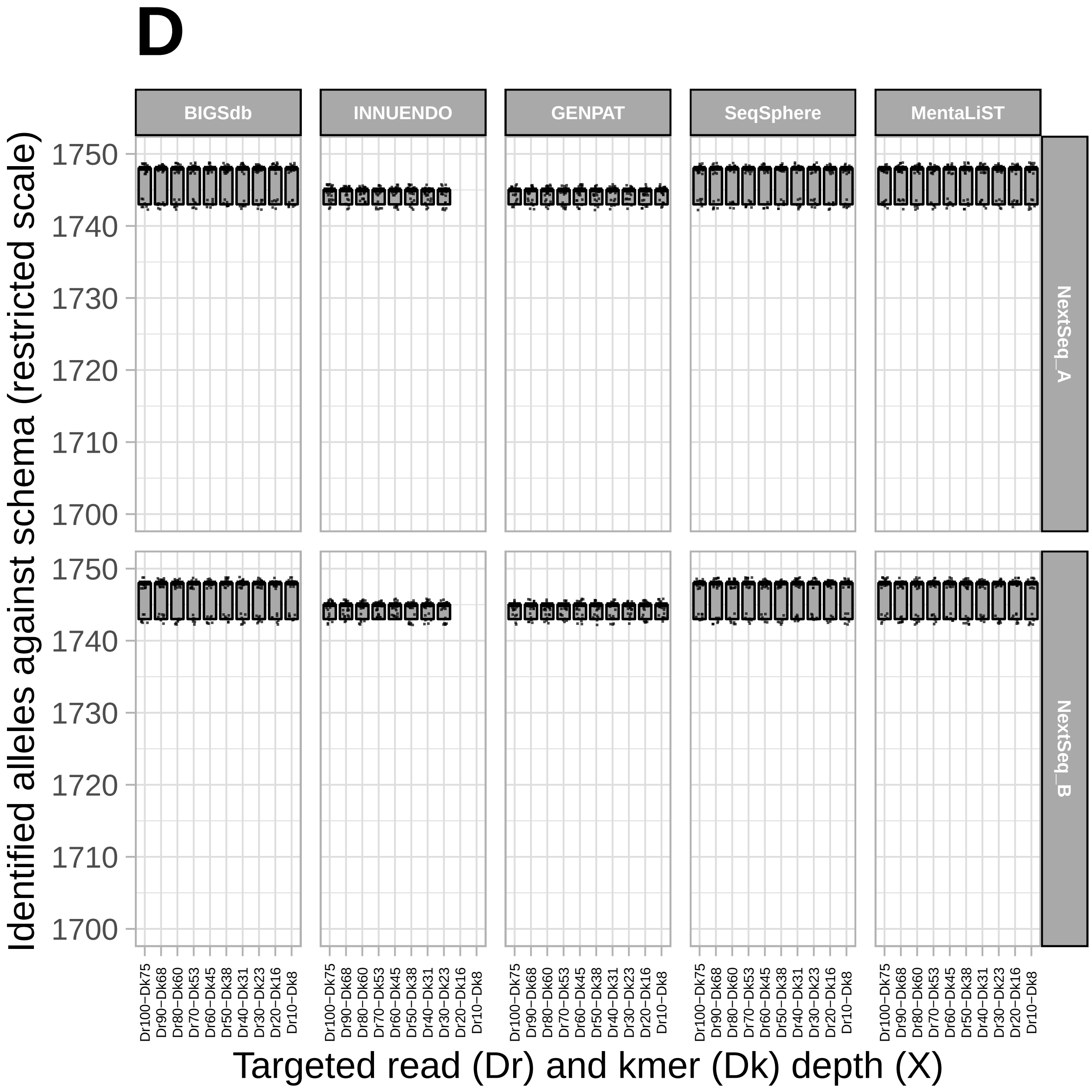
D
BIGSdb
INNUENDO
GENPAT
SeqSphere
MentaLiST
NextSeq_A
NextSeq_B
Identified alleles against schema (restricted scale)
1750
1740
1730
1720
1710
1700
Dr100−Dk75
Dr90−Dk68
Dr80−Dk60
Dr70−Dk53
Dr60−Dk45
Dr50−Dk38
Dr40−Dk31
Dr30−Dk23
Dr20−Dk16
Dr10−Dk8
Targeted read (Dr) and kmer (Dk) depth (X)

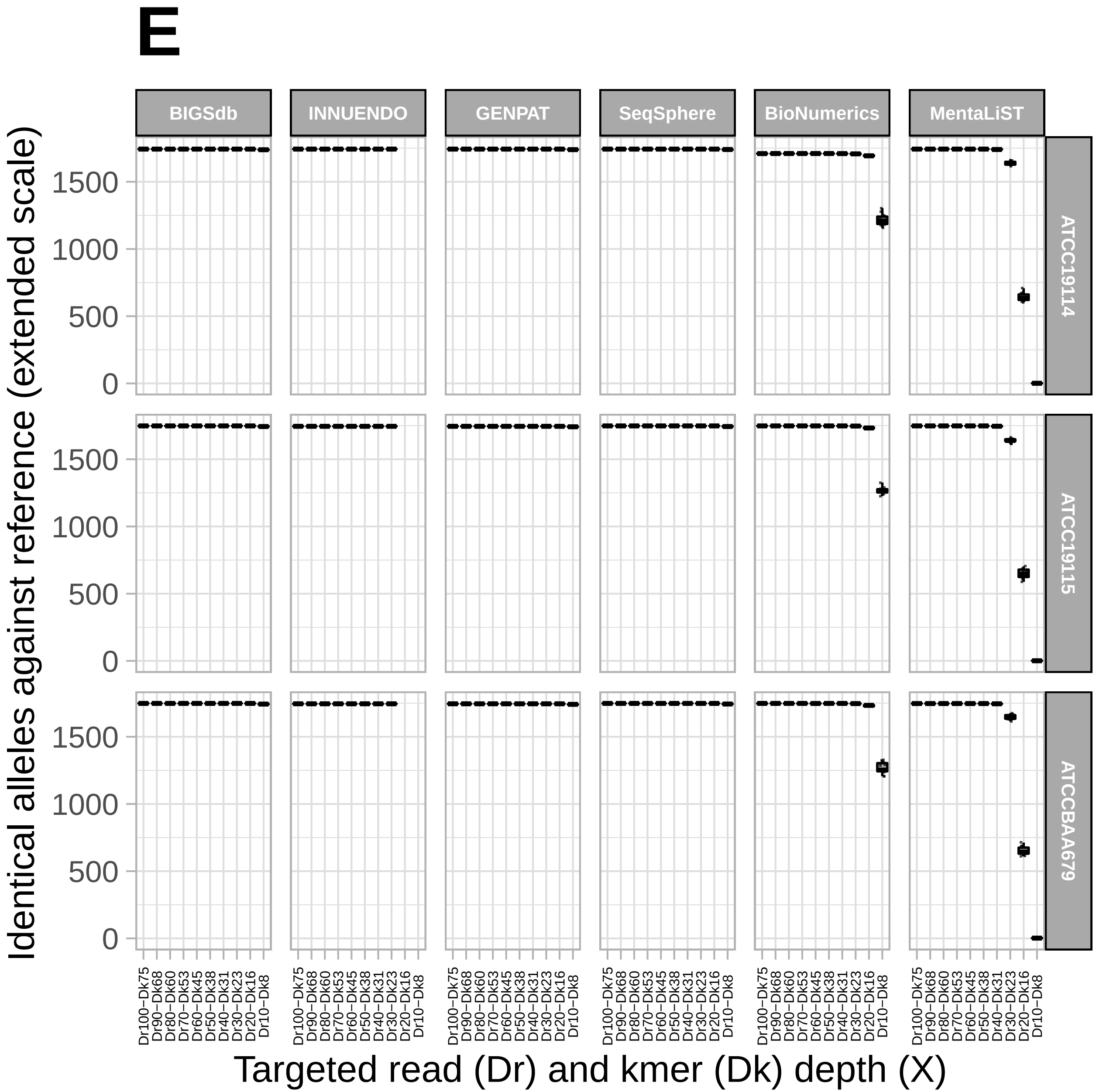
E
BIGSdb
INNUENDO
GENPAT
SeqSphere
BioNumerics
MentaLiST
ATCC19114
ATCC19115
ATCCBAA679
Identical alleles against reference (extended scale)
0
500
1000
1500
Dr100−Dk75
Dr90−Dk68
Dr80−Dk60
Dr70−Dk53
Dr60−Dk45
Dr50−Dk38
Dr40−Dk31
Dr30−Dk23
Dr20−Dk16
Dr10−Dk8
Targeted read (Dr) and kmer (Dk) depth (X)

F

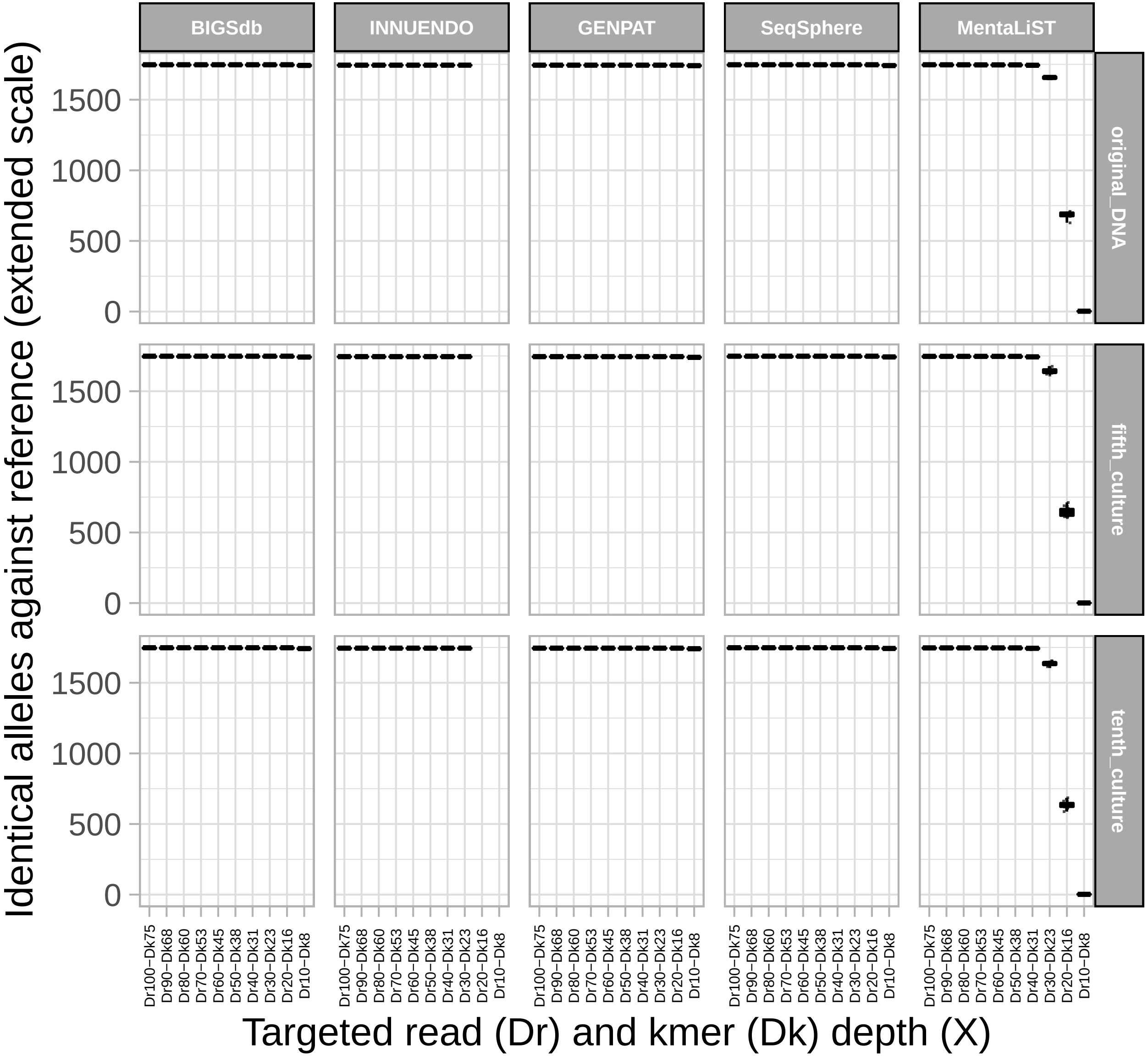
BIGSdb
INNUENDO
GENPAT
SeqSphere
MentaLiST
original_DNA
fifth_culture
tenth_culture
Identical alleles against reference (extended scale)
0
500
1000
1500
Dr100−Dk75
Dr90−Dk68
Dr80−Dk60
Dr70−Dk53
Dr60−Dk45
Dr50−Dk38
Dr40−Dk31
Dr30−Dk23
Dr20−Dk16
Dr10−Dk8
Targeted read (Dr) and kmer (Dk) depth (X)

G

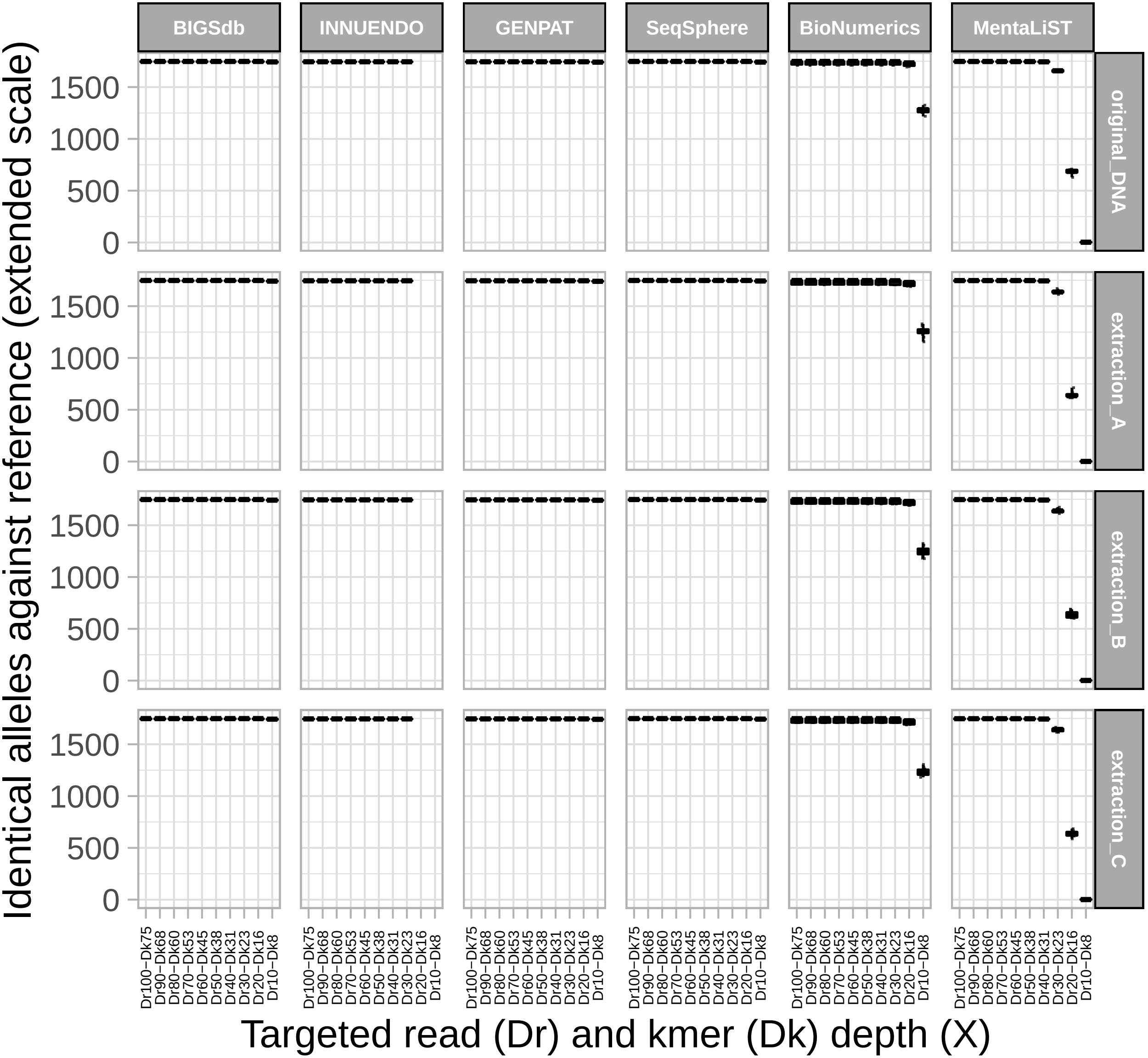

BIGSdb
INNUENDO
GENPAT
SeqSphere
BioNumerics
MentaLiST
original_DNA
extraction_A
extraction_B
extraction_C
Identical alleles against reference (extended scale)
0
500
1000
1500
Dr100−Dk75
Dr90−Dk68
Dr80−Dk60
Dr70−Dk53
Dr60−Dk45
Dr50−Dk38
Dr40−Dk31
Dr30−Dk23
Dr20−Dk16
Dr10−Dk8
Targeted read (Dr) and kmer (Dk) depth (X)

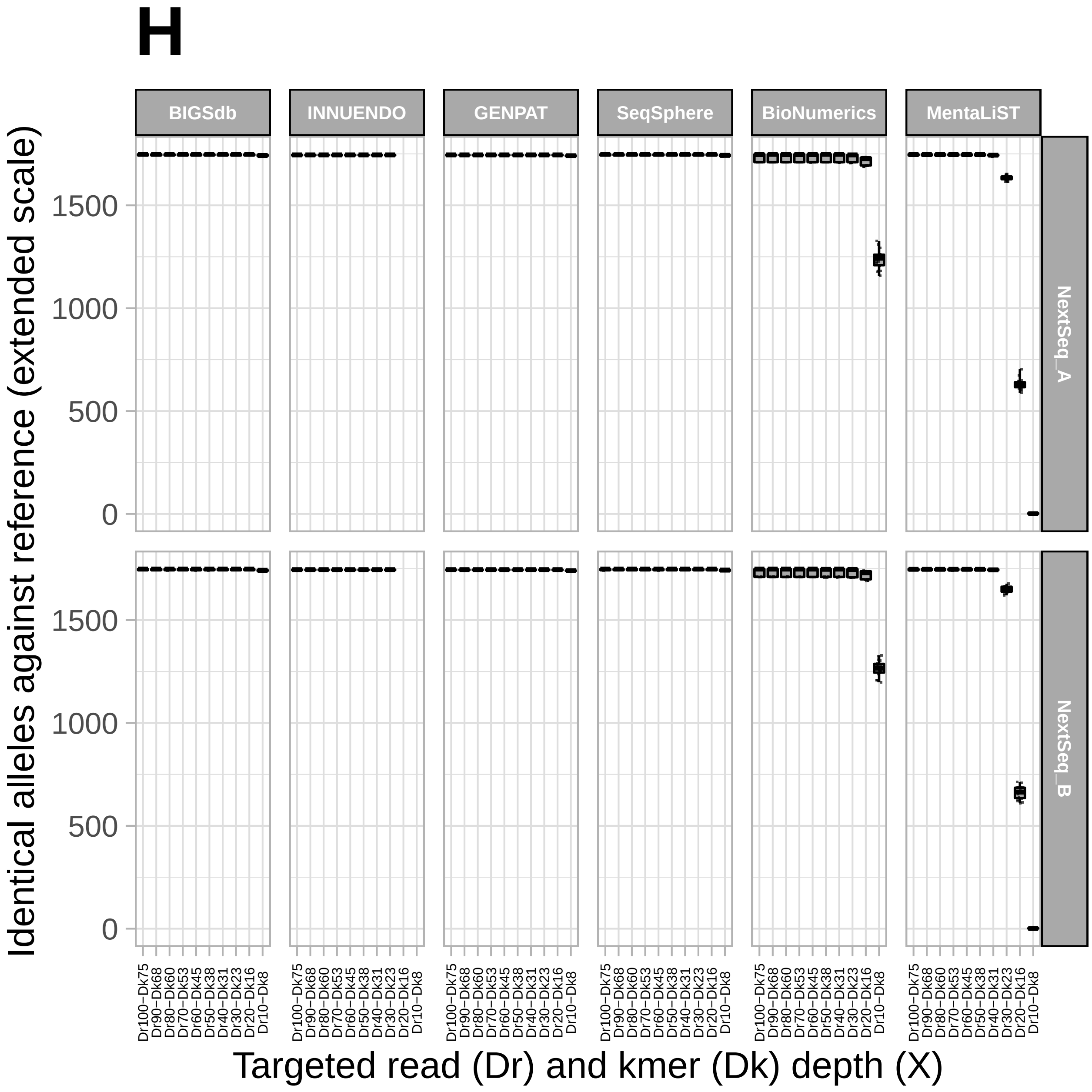

H
BIGSdb
INNUENDO
GENPAT
SeqSphere
BioNumerics
MentaLiST
NextSeq_A
NextSeq_B
Identical alleles against reference (extended scale)
0
500
1000
1500
Dr100−Dk75
Dr90−Dk68
Dr80−Dk60
Dr70−Dk53
Dr60−Dk45
Dr50−Dk38
Dr40−Dk31
Dr30−Dk23
Dr20−Dk16
Dr10−Dk8
Targeted read (Dr) and kmer (Dk) depth (X)

I

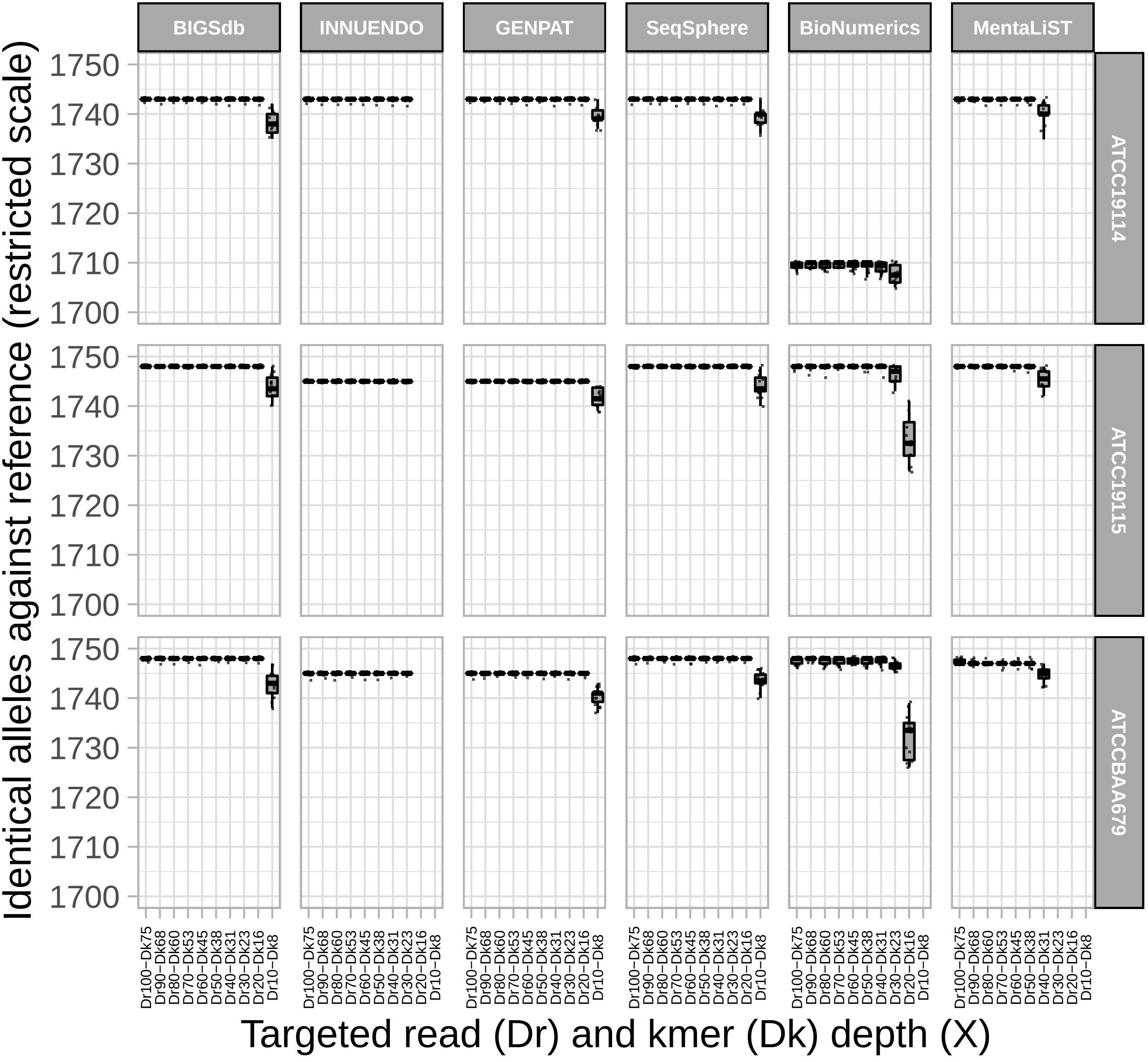

BIGSdb
INNUENDO
GENPAT
SeqSphere
BioNumerics
MentaLiST
ATCC19114
ATCC19115
ATCCBAA679
Identical alleles against reference (restricted scale)
1750
1740
1730
1720
1710
1700
Dr100−Dk75
Dr90−Dk68
Dr80−Dk60
Dr70−Dk53
Dr60−Dk45
Dr50−Dk38
Dr40−Dk31
Dr30−Dk23
Dr20−Dk16
Dr10−Dk8
Targeted read (Dr) and kmer (Dk) depth (X)

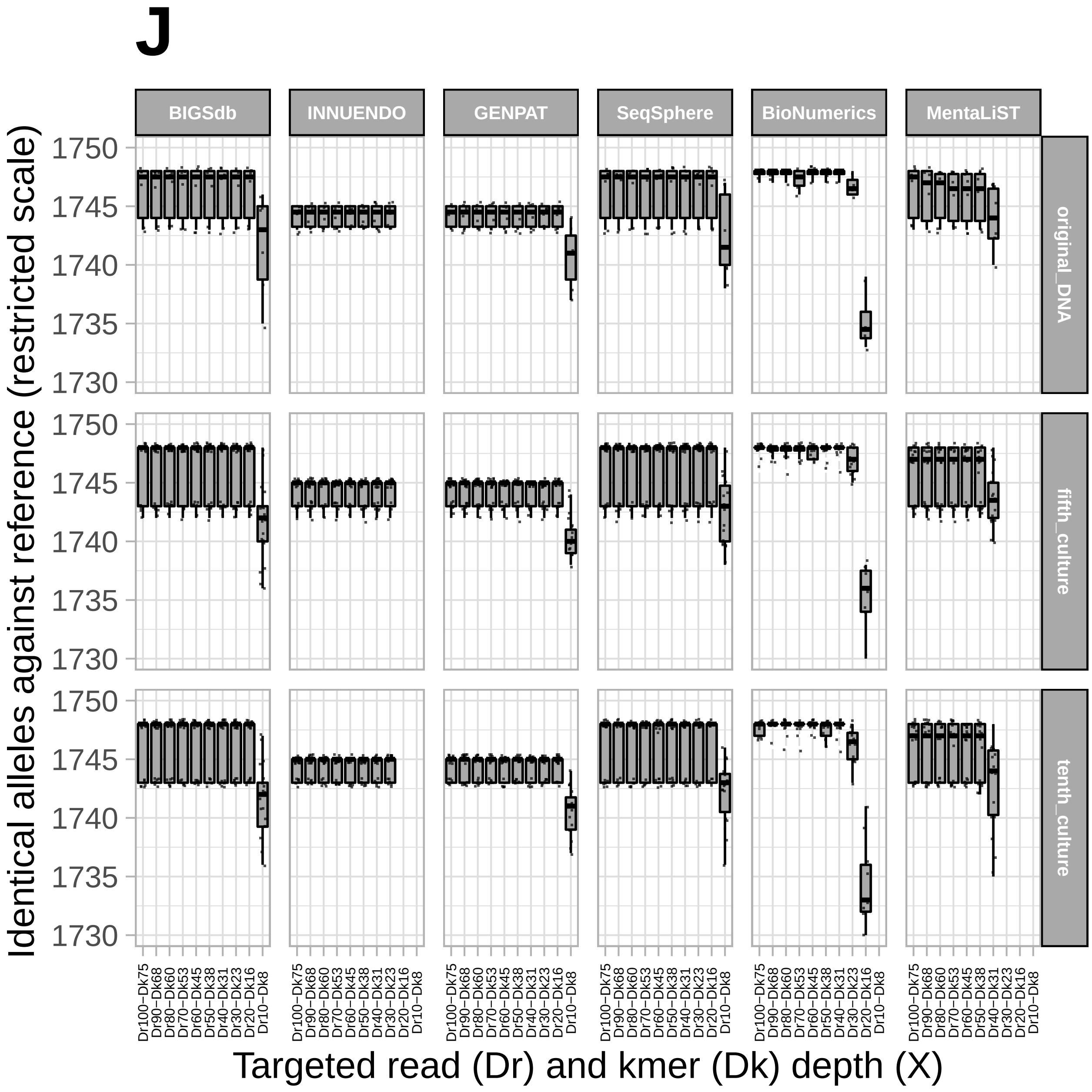
J
BIGSdb
INNUENDO
GENPAT
SeqSphere
BioNumerics
MentaLiST
original_DNA
fifth_culture
tenth_culture
Identical alleles against reference (restricted scale)
1750
1745
1740
1735
1730
Dr100−Dk75
Dr90−Dk68
Dr80−Dk60
Dr70−Dk53
Dr60−Dk45
Dr50−Dk38
Dr40−Dk31
Dr30−Dk23
Dr20−Dk16
Dr10−Dk8
Targeted read (Dr) and kmer (Dk) depth (X)

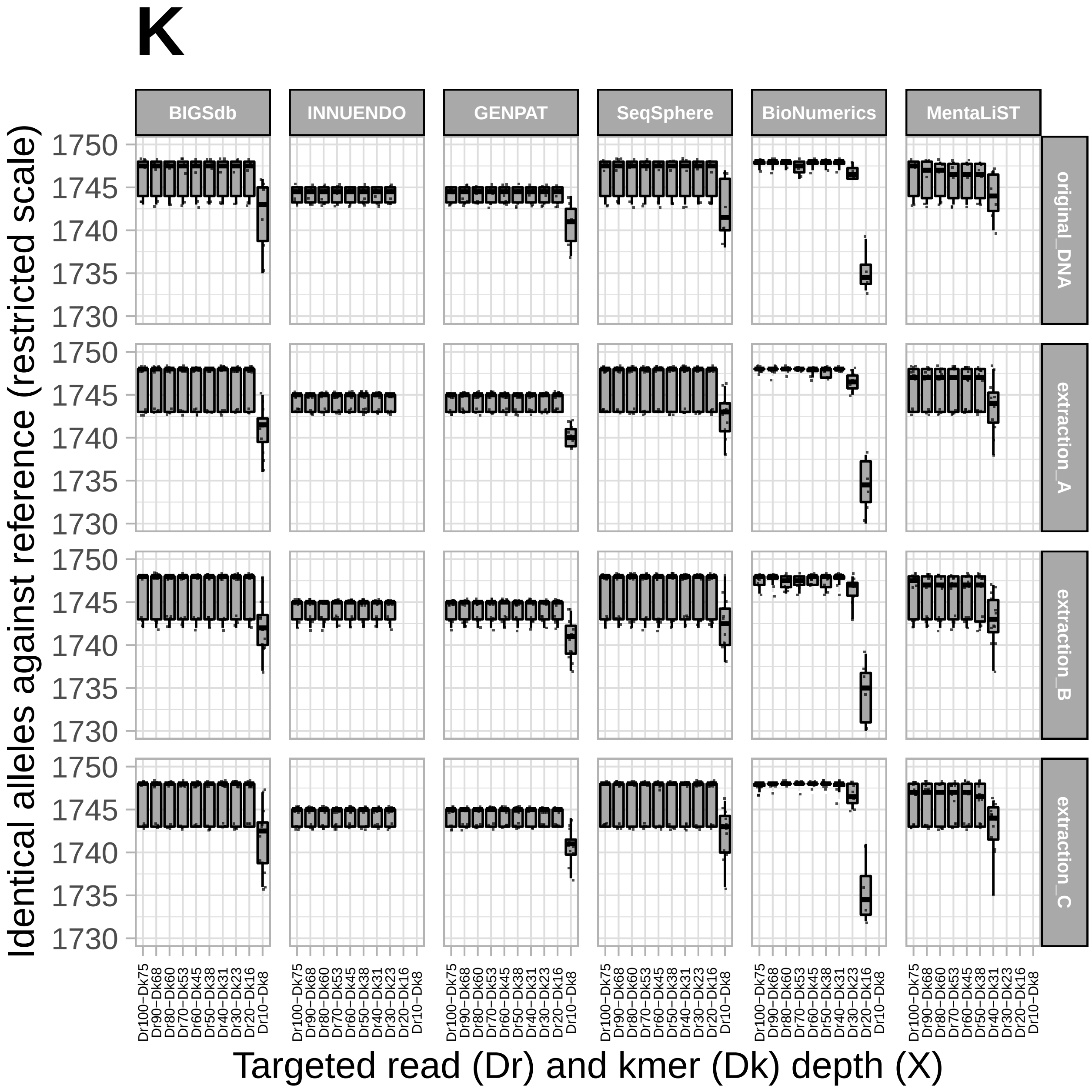
K
BIGSdb
INNUENDO
GENPAT
SeqSphere
BioNumerics
MentaLiST
original_DNA
extraction_A
extraction_B
extraction_C
1750
1745
1740
1735
1730
Identical alleles against reference (restricted scale)
Dr100−Dk75
Dr90−Dk68
Dr80−Dk60
Dr70−Dk53
Dr60−Dk45
Dr50−Dk38
Dr40−Dk31
Dr30−Dk23
Dr20−Dk16
Dr10−Dk8
Targeted read (Dr) and kmer (Dk) depth (X)

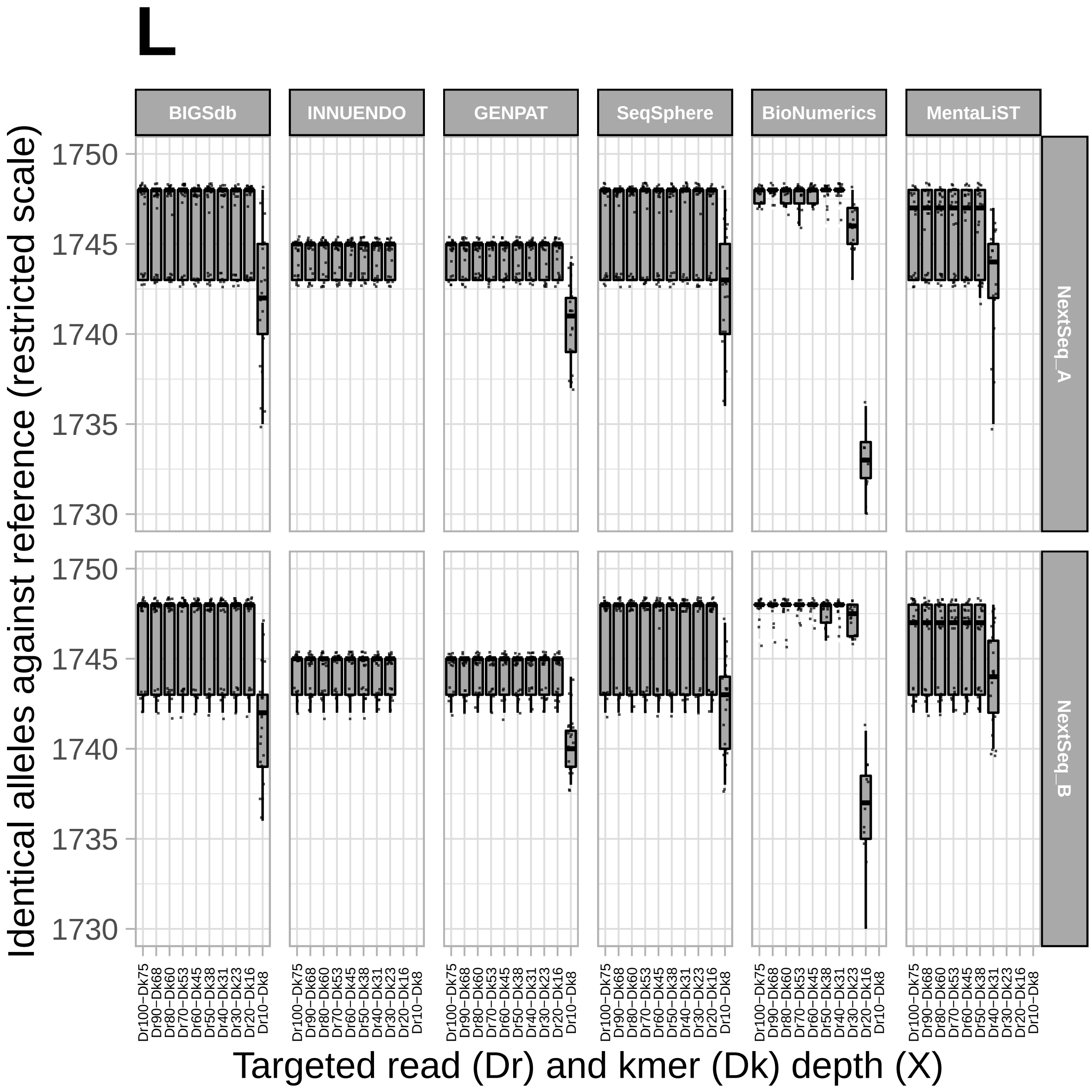

L
BIGSdb
INNUENDO
GENPAT
SeqSphere
BioNumerics
MentaLiST
NextSeq_A
NextSeq_B
Identical alleles against reference (restricted scale)
1750
1745
1740
1735
1730
Dr100−Dk75
Dr90−Dk68
Dr80−Dk60
Dr70−Dk53
Dr60−Dk45
Dr50−Dk38
Dr40−Dk31
Dr30−Dk23
Dr20−Dk16
Dr10−Dk8
Targeted read (Dr) and kmer (Dk) depth (X)
