# Supplementary material for: In vitro and in silico parameters for precise cgMLST typing of Listeria monocytogenes
Source: BMC Genomics. 2022 Mar 26;23:235. doi: 10.1186/s12864-022-08437-4 (PMC8961897; doi:10.1186/s12864-022-08437-4)
Supplement: Supplementary file 8 — Additional file 8 Minimum spanning trees (MSTs) representing the impact on clustering of cgMLST workflows BIGSdb (A: n = 423), INNUENDO (B: n = 339), GENPAT (C: n = 423), SeqSphere (D: n = 423), BioNumerics (E: n = 423) and MentaLiST (F: n = 423), of Listeria monocytogenes reference genomes (i.e. ATCC19114, ATCC19115 and ATCCBAA679 on the left of each workflow) and targeted depth of coverage (i.e. on the right of each workflow) from downsampled paired-end reads (i.e. 2x150bp). The MSTs were built with BioNumerics ignoring missing data. The MST clusters of at least two genomes, one node and allele differences ≤7, were highlighted in grey. The targeted read depth (Dr: 10X, 20X, 30X, 40X, 50X, 60X, 70X, 80X, 90X and 100X) were prepared according to kmer depth (Dk): 8X, 15X, 23X, 30X, 38X, 45X, 52X, 60X, 67X, 75X) setting of BBNorm (read length R = 150 and kmer size K = 30). Because of internal firewall, the INNUca assembler integrated into the cgMLST workflow INNUENDO cannot not perform assemblies of paired-end reads with read depth of coverage of 20X (n = 42) and 10X (n = 42). [file 12864_2022_8437_MOESM8_ESM.pdf]

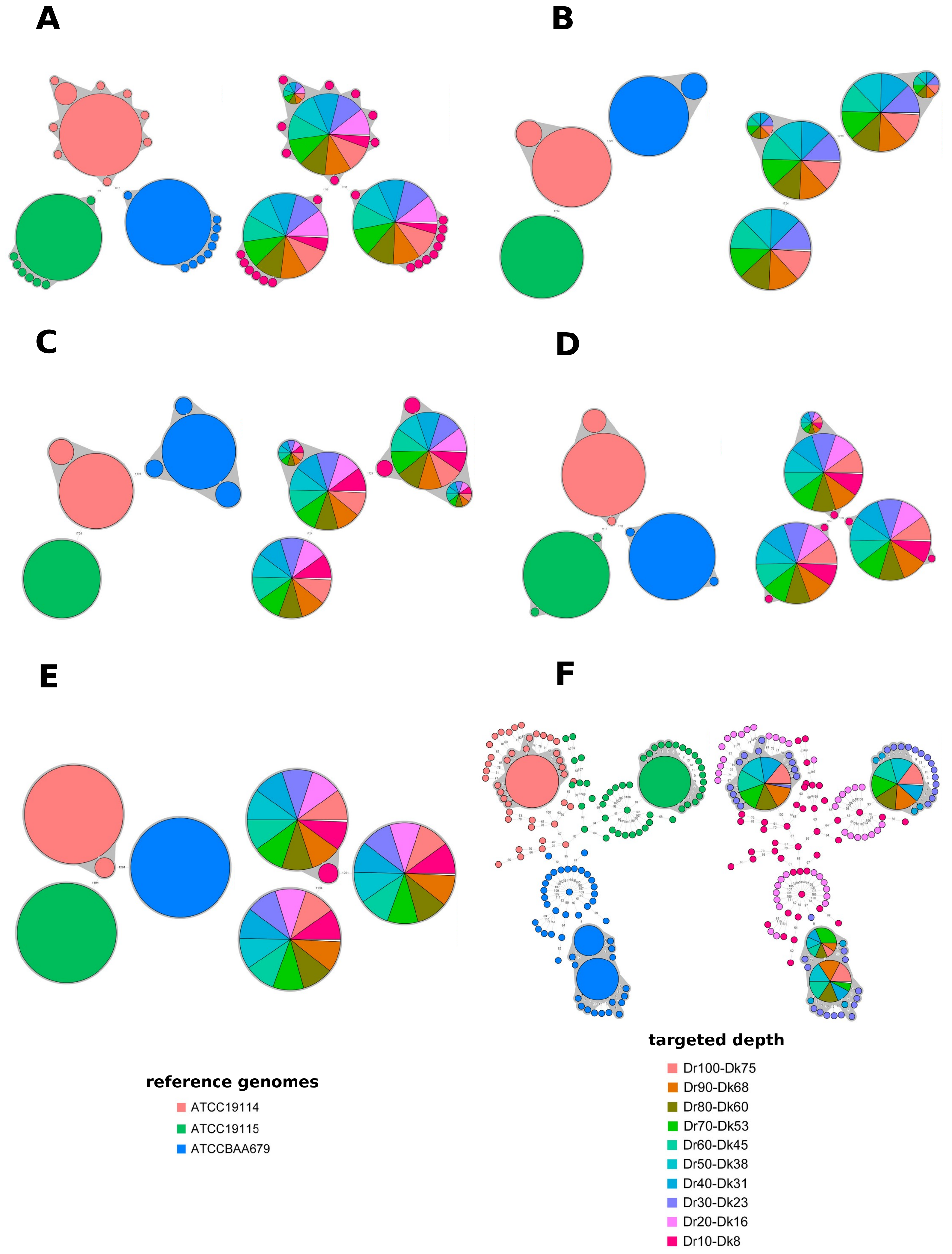
A
B
C
D
E
F
targeted depth
Dr100-Dk75
Dr90-Dk68
Dr80-Dk60
Dr70-Dk53
Dr60-Dk45
Dr50-Dk38
Dr40-Dk31
Dr30-Dk23
Dr20-Dk16
Dr10-Dk8
reference genomes
ATCC19114
ATCC19115
ATCCBAA679
