# Supplementary material for: In vitro and in silico parameters for precise cgMLST typing of Listeria monocytogenes
Source: BMC Genomics. 2022 Mar 26;23:235. doi: 10.1186/s12864-022-08437-4 (PMC8961897; doi:10.1186/s12864-022-08437-4)
Supplement: Supplementary file 9 — Additional file 9 Box-plots representing the impact of downsampled paired-end reads (i.e. 2x150bp), at extended (A and B) or restricted (C and D) scales of identical alleles against reference circular genomes, spiting (A and C) or merging (B and D) reference genomes of Listeria monocytogenes (i.e. ATCC19114, ATCC19115 and ATCCBAA679), on cgMLST outcomes from the assembly-based workflow alone (BioNumericsAB: n = 420), or in combination with the assembly-free workflow implemented in BioNumerics (version 7.6.2) (BioNumericsAF: n = 420). [file 12864_2022_8437_MOESM9_ESM.pdf]

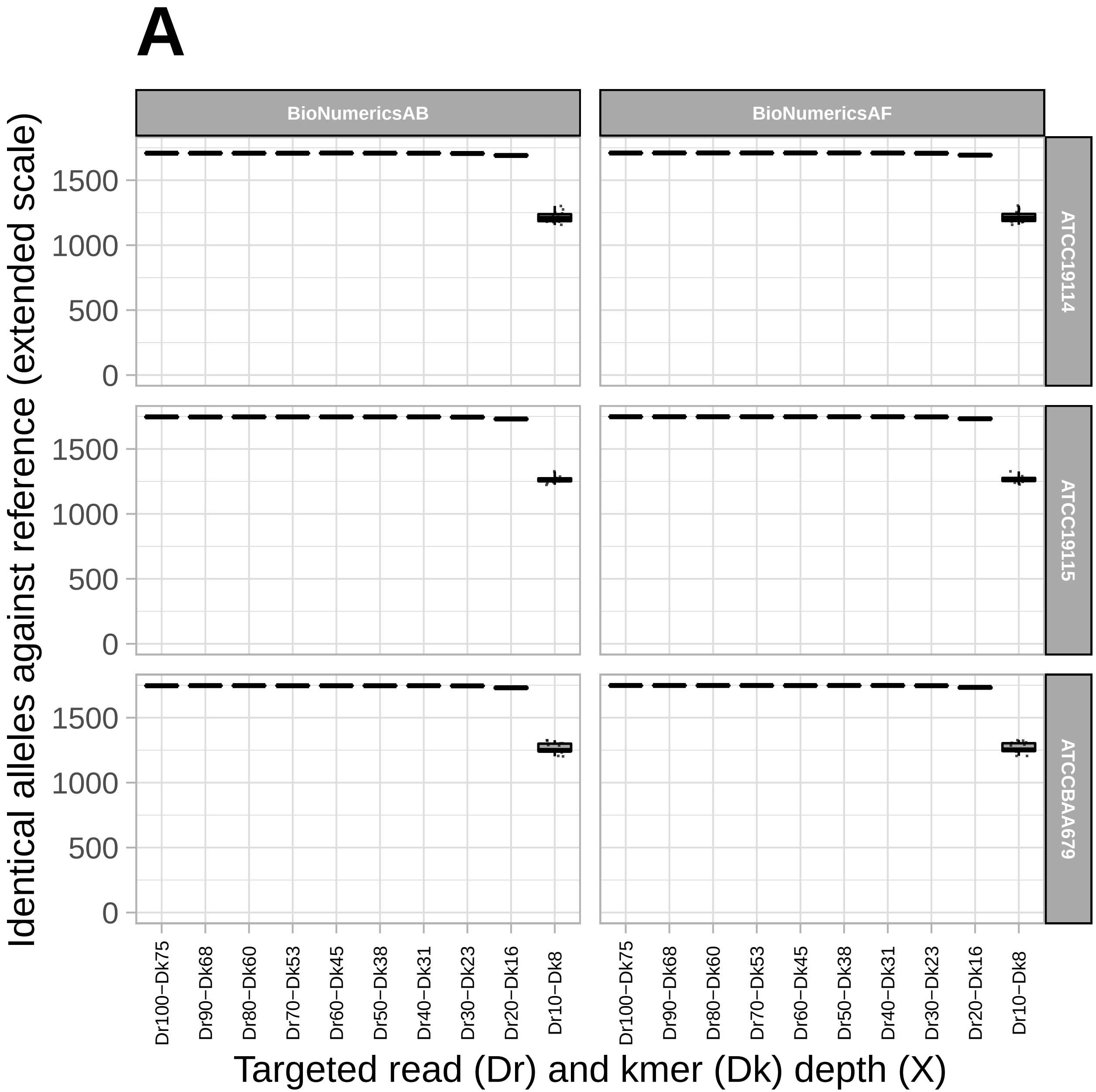
A
BioNumericsAB
BioNumericsAF
ATCC19114
ATCC19115
ATCCBAA679
Identical alleles against reference (extended scale)
0
500
1000
1500
Dr100−Dk75
Dr90−Dk68
Dr80−Dk60
Dr70−Dk53
Dr60−Dk45
Dr50−Dk38
Dr40−Dk31
Dr30−Dk23
Dr20−Dk16
Dr10−Dk8
Targeted read (Dr) and kmer (Dk) depth (X)

B

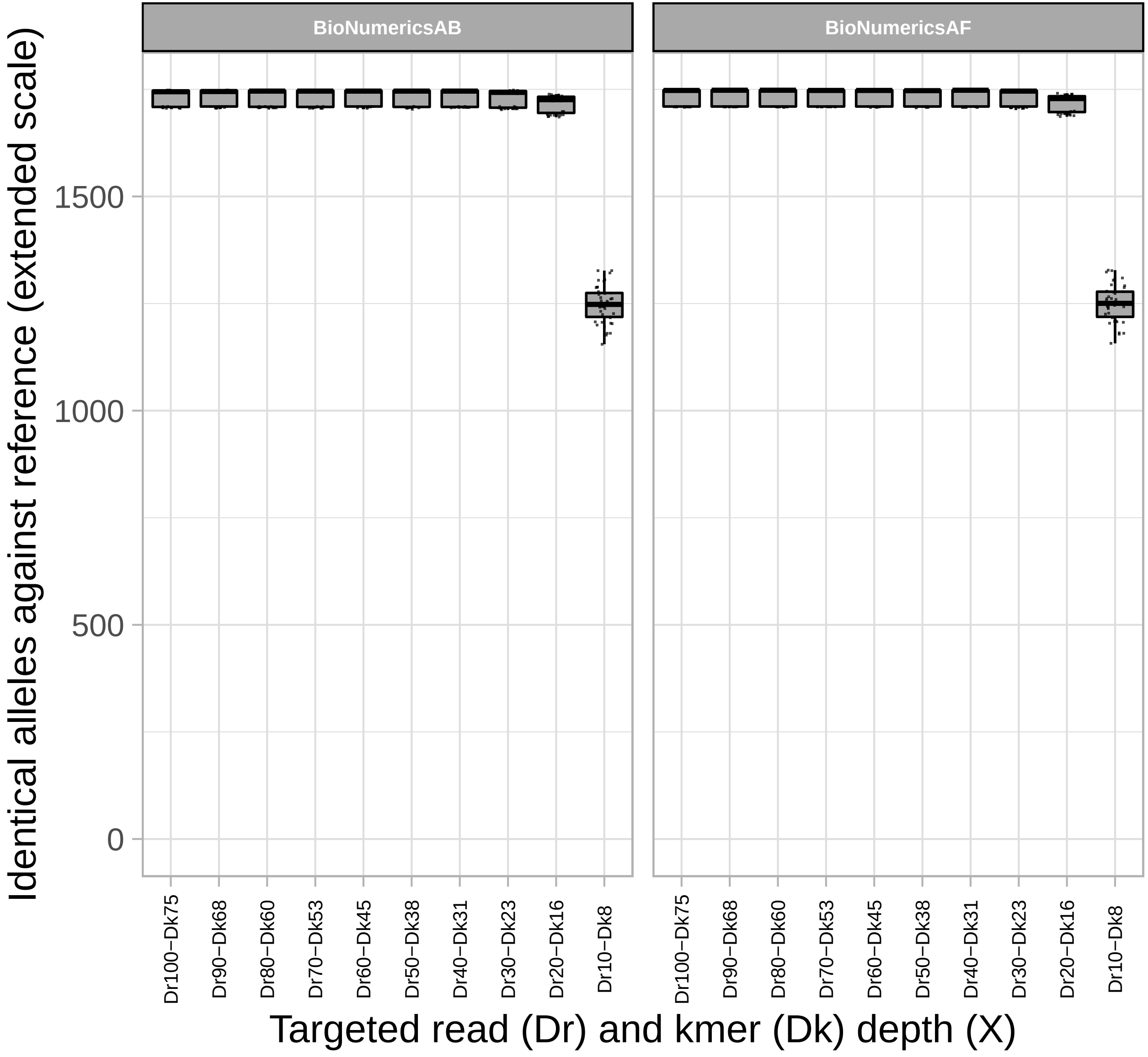
BioNumericsAB
BioNumericsAF
Identical alleles against reference (extended scale)
1500
1000
500
0
Dr100−Dk75
Dr90−Dk68
Dr80−Dk60
Dr70−Dk53
Dr60−Dk45
Dr50−Dk38
Dr40−Dk31
Dr30−Dk23
Dr20−Dk16
Dr10−Dk8
Targeted read (Dr) and kmer (Dk) depth (X)

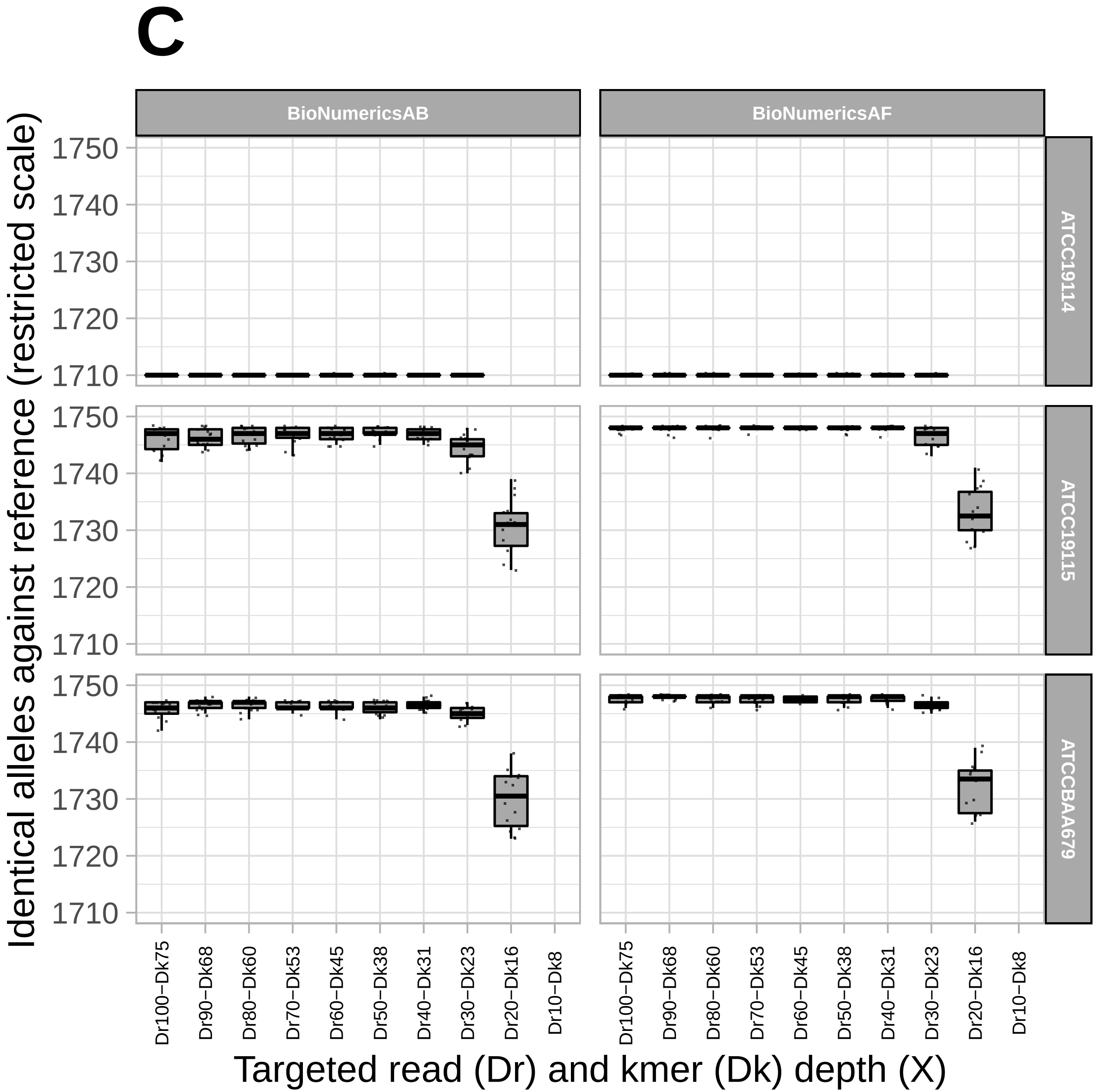

C
BioNumericsAB
BioNumericsAF
ATCC19114
ATCC19115
ATCCBAA679
Identical alleles against reference (restricted scale)
1750
1740
1730
1720
1710
Dr100−Dk75
Dr90−Dk68
Dr80−Dk60
Dr70−Dk53
Dr60−Dk45
Dr50−Dk38
Dr40−Dk31
Dr30−Dk23
Dr20−Dk16
Dr10−Dk8
Targeted read (Dr) and kmer (Dk) depth (X)

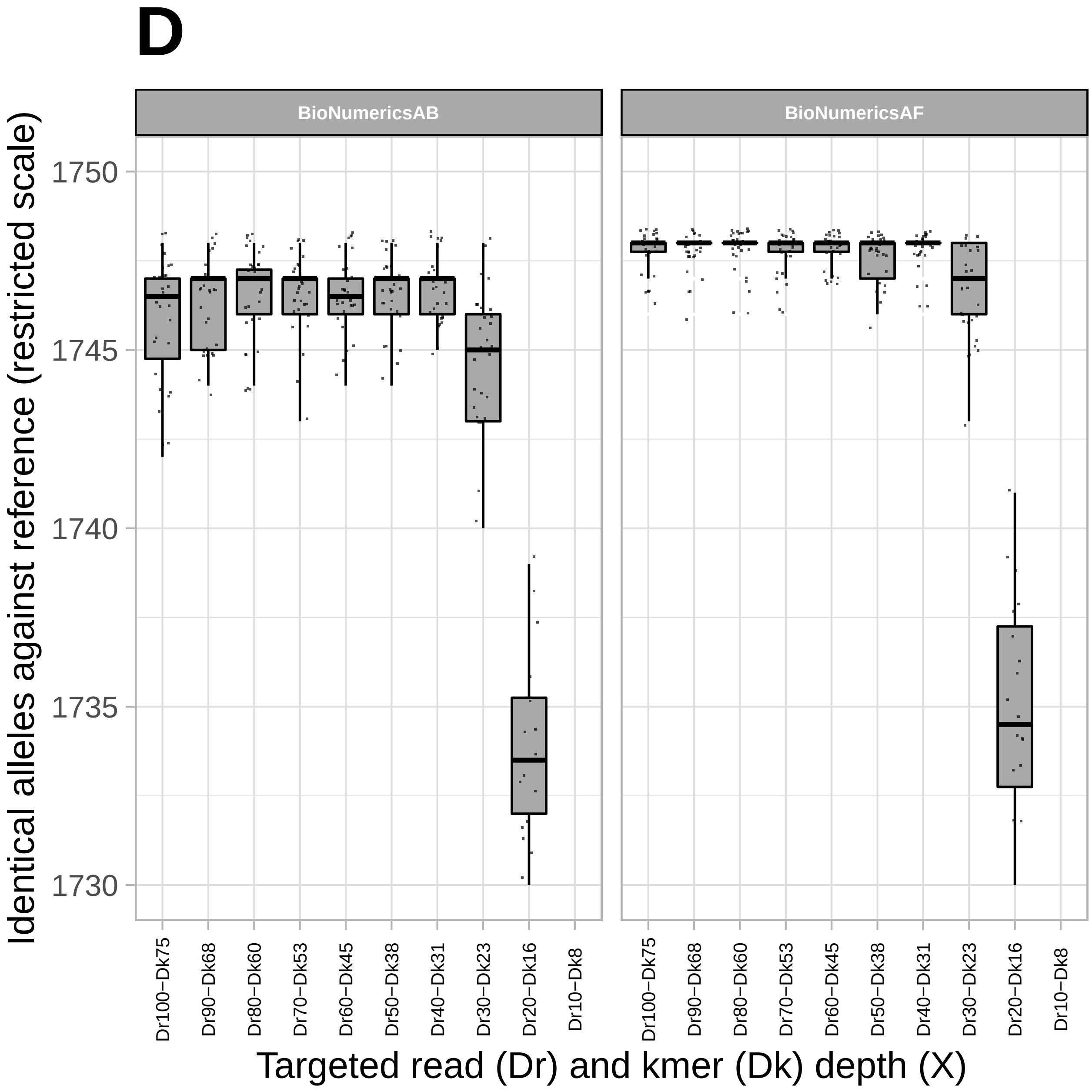
D
BioNumericsAB
BioNumericsAF
Identical alleles against reference (restricted scale)
1750
1745
1740
1735
1730
Dr100−Dk75
Dr90−Dk68
Dr80−Dk60
Dr70−Dk53
Dr60−Dk45
Dr50−Dk38
Dr40−Dk31
Dr30−Dk23
Dr20−Dk16
Dr10−Dk8
Targeted read (Dr) and kmer (Dk) depth (X)
